# Stabilizing Azaheptacenes

**DOI:** 10.1021/jacs.3c13629

**Published:** 2024-02-27

**Authors:** Wansheng Zong, Nikolai Hippchen, Nico Zeitter, Steffen Maier, Philipp Ludwig, Frank Rominger, Jan Freudenberg, Uwe H. F. Bunz

**Affiliations:** † Organisch-Chemisches Institut, Ruprecht-Karls-Universität Heidelberg, Im Neuenheimer Feld 270, 69120 Heidelberg, Germany

## Abstract

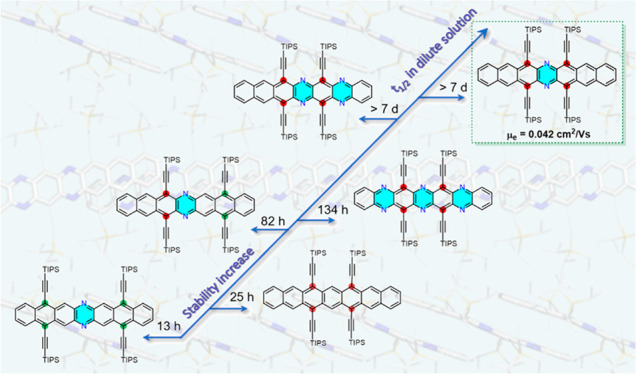

The symmetrical 7,16-diaza-6,8,15,17-tetrakis(triisopropylsilylethynyl)heptacene was obtained by a Pd-catalyzed reaction of a 2,3-diamino-1,4-diethynylanthracene with a 2,3-dibromo-1,4-diethynyl anthracene. Positioning the TIPS-ethynyl groups adjacent to the central ring suppresses dimerization via [4+4] cycloadditions and Diels–Alder reactions; the middle pyrazine ring renders this species stable to oxidation. A single crystal structure was obtained, and thin film transistors with μ_n_ = 0.042 cm^2^ V^–1^ s^–1^ were produced. Transposition of the alkynyl groups into the 5,8,15,18-positions with a quinoxaline unit in the center of the heptacene decreases the stability, as does the introduction of two more nitrogen atoms into the 5,18-positions. The hydrocarbon 6,8,15,17-tetrakis(triisopropylsilylethynyl)heptacene is reasonably stable with a half-life of 25 h in solution. Four correctly placed TIPS-ethynyl groups protect heptacene cores.

Pentacene, a benchmark p-channel semiconductor,^1,2^ is reasonably stable and processable by evaporation.^3,4^ Two TIPS-ethynyl groups at the central ring^5^ render it solution processable^6−8^ and add stability.^9^ Larger silyl groups (*t*Bu_3_Si, (Me_3_Si)Si) give reasonably stable hexacenes^10^ and the marginally stable but isolable heptacene **1**(11) (Figure 1). Isoelectronic azaacenes are obtained by fusing pyrazines onto the acene—double silylethynylation—generates stable azapentacenes^12−16^ and azahexacenes.^17−19^ In contrast to their hydrocarbon analogues,^20−22^ isolable azaheptacenes are unknown, despite Dutt’s claim in 1926.^23,24^**DAH3**^25^ (Figure 1), the only reported azaheptacene, is reactive in solution. Upon crystallization, a mixture of butterfly dimers forms by [4+4] cycloaddition of the rings adjacent to the pyrazine.

**Figure 1:**
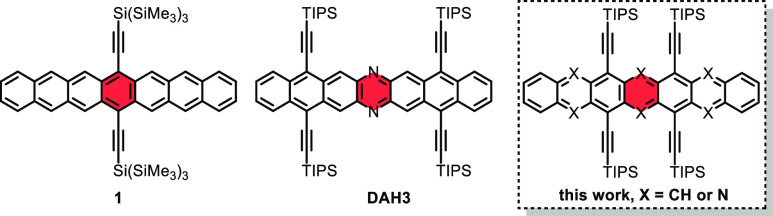
Known heptacene **1**, diazaheptacene **DAH3**, and the (aza)heptacenes reported herein.

Azaacenes are, as a rule, more stable than acenes: Pyrazine rings increase the oxidation potential^26,27^ and retard oxidation/*endo*-peroxide formation, a major decomposition path of large acenes under ambient conditions.^9,28^ They prevent [4+4] or [4+2] cycloadditions (with ethynyl substituents)^29,30^ at the pyrazine ring,^10,17^ but how many pyrazines are needed and is there an optimum number? Can too many pyrazines decrease the azaacenes’ stability with spontaneous reduction to their dihydro species?^17,31,32^

Transposition of the silylethynyl groups toward the center results in **DAH1**, stable in solution and as a solid. The comparison of **DAH1** to **Hep** highlights the nitrogens’ role in blocking degradation via oxidation. **DAH1**, **DAH2**, and **DAH3**(25) differ in the TIPS-ethynyls’ placement, and for **Hep**, **DAH1**, **TAH**, and **HAH** the nitrogen content in heptacenes with a fixed TIPS-ethynylation pattern (6, 8, 15, 17 positions) was investigated.

The syntheses of the five target (aza)acenes (Scheme 1) entails Pd-catalyzed Buchwald–Hartwig coupling^33,34^ of **2a**(35) to **3a** into **DAH1-H**_**2**_ (63%). Oxidation with MnO_2_ furnished **DAH1** (72%) and byproduct **S1** (10%, see SI, Figures S24, S36, and S37) after rearrangement of **DAH1-H**_**2**_. Combining **2a** with **3b**(25) leads to the non-centrosymmetric **DAH2-H**_**2**_. It is oxidized to **DAH2** by MnO_2_ (85%). The synthesis of **TAH-H**_**2**_ and **HAH-H**_**2**_ exploits *ortho*-quinone **4**, obtained from dibromodiiodoveratrole by demethylation and oxidation; **4** dimerizes spontaneously^36,37^ and was immediately combined with **2a** or **2b**(35) into **5a,b**. **5a** reacted with 5 equiv of TIPS-acetylene under Sonogashira^38^ conditions (50 °C) into multiply ethynylated *N,N*′-dihydro-intermediates, with **6a** isolated in 39% yield. Surprisingly, **5b** selectively transforms into **6b** with 10 equiv of TIPS-acetylene at room temperature in 75% yield. **6a,b** were Buchwald–Hartwig coupled^33,34^ to *ortho*-phenylenediamine to give **TAH-H**_**2**_ and **HAH-H**_**2**_. It is surprising that the Sonogashira/Buchwald–Hartwig coupling worked so well, considering halide selectivity and steric hindrance. MnO_2_ converted **TAH-H**_**2**_ into **TAH** in 5 min, while **HAH-H**_**2**_ was, even after 5 h, only partially oxidized into **HAH**. The azaheptacenes were isolated by column chromatography on silica. **DAH1**, **DAH2**, **TAH**, and **HAH** are microcrystalline solids, characterized by NMR, UV–vis, and IR spectroscopy, mass spectrometry, cyclic voltammetry, UPLC, and elemental as well as single-crystal structure analysis. **Hep** was synthesized according to the literature (see SI).^39^

**Scheme 1:**
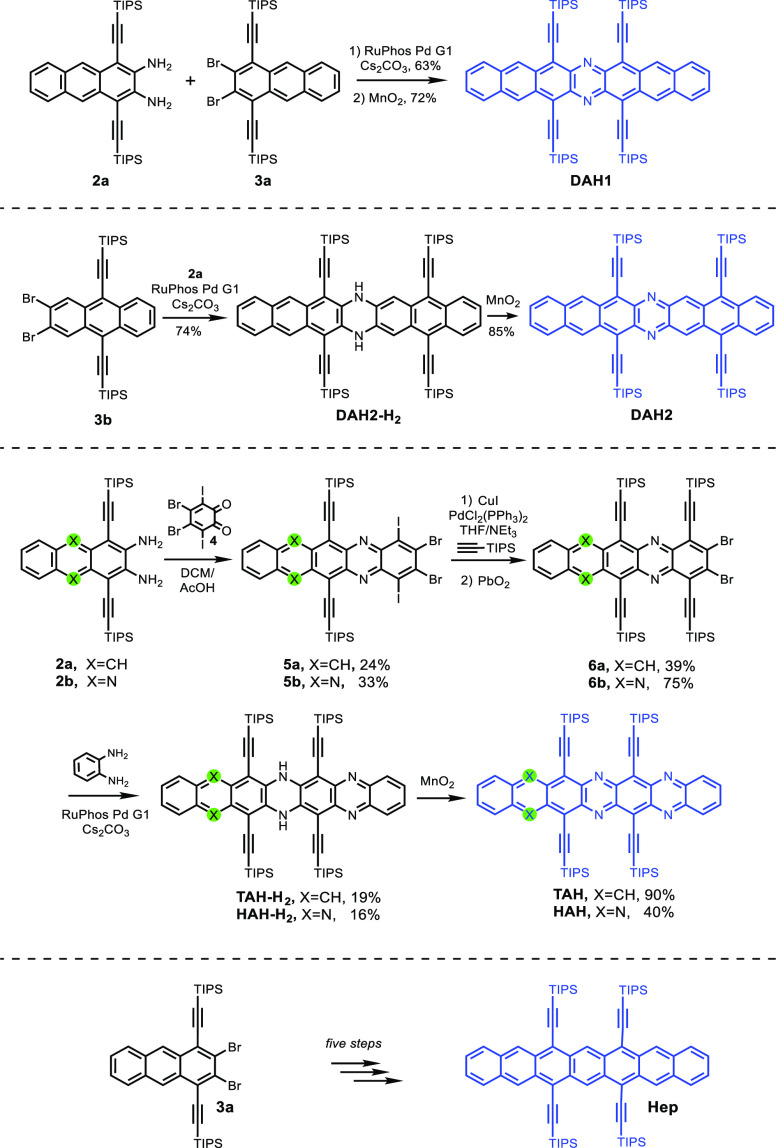
Synthesis of (Aza)heptacenes

Figure S12 (SI) displays the UV–vis spectra of the *N,N*′-dihydro compounds. Upon oxidation (Figure 2a) we observe acene p-bands with absorption onsets for **DAH1**, **DAH2**, **TAH**, and **HAH** red-shifted to 1074, 1064, 1152, and 1045 nm, proof of azaheptacene formation (Table 1). **TAH**’s absorption is the most red-shifted, a consequence of its donor–acceptor character.^40^**Hep** is the most blue-shifted congener (absorption onset: 950 nm). The heptacene p-bands are broadened and/or display shoulders at the longest wavelengths, attributed to their arising diradical character,^41^ although their NMR spectra are well-resolved. The LUMOs of **DAH1**, **HAH**, and **Hep** are evenly distributed over the molecular skeleton, while the HOMO of **TAH** has small coefficients at the pyrazine rings,^42^ resulting in a decreased band gap (SI, Figure S1). **Hep**, **DAH1**, and **DAH2** show two reduction and two oxidation waves (SI, Figure S5), while **TAH** and **HAH** only display two and three reduction events, respectively (SI, Figure S5). **TAH**’s and **HAH**’s electron affinities are <−4.10 eV; their radical anions might be stable in air and will be reported elsewhere.

**Figure 2:**
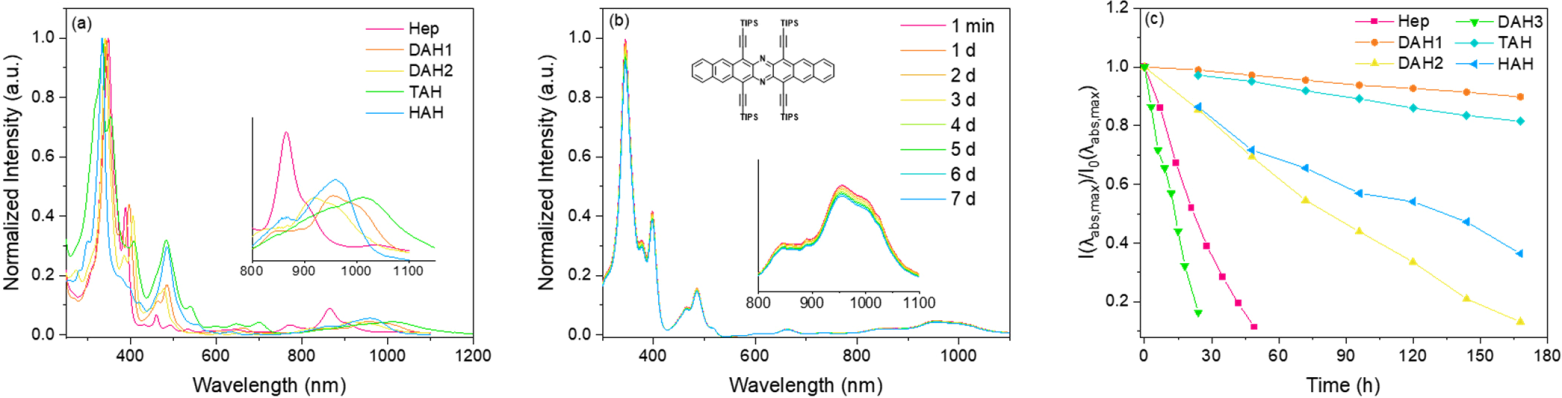
(a) Normalized UV–vis absorption spectra of (aza)heptacenes in DCM (10^–5^ mol L^–1^). (b) Time-dependent evolution of UV–vis spectra of **DAH1** (10^–5^ mol L^–1^ in dry DCM) under ambient light and atmosphere. Inset: magnification of the p-bands. (c) Evolution of UV–vis absorption intensities at λ_abs,max_ for (aza)heptacenes under ambient conditions (UV–vis spectra: see SI, Figure S13).

**Table 1:** Photophysical and Calculated Properties of (Aza)heptacenes

compd	λ_abs on_^a^ [nm]	λ_abs max_^b^ [nm]	*E*_g meas_^c^/*E*g cal^d^ [eV]	*E*_1/2_^red1^^e^ [V]	EA^f^/*E*_LUMO_^g^ [eV]	IP^h^/*E*_HOMO_^g^ [eV]
Hep	950	865	1.30/1.22	–1.15	–3.65/-3.43	–4.95/-4.65
DAH1	1074	957	1.15/1.17	–0.72	–4.08/-3.71	–5.23/-4.88
DAH2	1064	917	1.17/1.19	–0.77	–4.03/–3.70	–5.20/–4.89
TAH	1152	1011	1.08/1.12	–0.69	–4.11/–4.02	–5.19/–5.14
HAH	1045	961	1.19/1.19	–0.60	–4.20/–4.19	–5.39/–5.38

a Onset of the lowest energy absorption maxima.

b Most intense absorption of the p-band.

c Optical gap calculated by λ_onset_.

d HOMO–LUMO gap calculated by DFT calculation.

e First reduction potentials measured by cyclic voltammetry in DCM against Fc/Fc^+^ as the internal standard (−4.80 eV) using a Pt working electrode and Bu_4_NPF_6_ as electrolyte.

f Electron affinities (EA) estimated from first reduction potentials; EA = −4.80 eV – *E*_red_.^43^

g FMO values calculated by DFT calculation^44^ (Gaussian16 B3LYP, def2TZVP; TMS groups were used instead of TIPS).

h Ionization potential (IP) = EA – *E*_g meas_.

**DAH3**, with the largest spacing between the TIPS-ethynyl substituents, is the *computationally* most stable isomer with respect to total energies neglecting decomposition (Table S1). Successively taken NMR (SI, Figures S6 to S11) and UV–vis spectra (Figures 2b,c and S13) illustrate the stability of **DAH1**, **DAH2**, and **DAH3**, highlighting the effect of the TIPS-ethynyl substituent pattern on diazaheptacenes. The NMR spectrum of **DAH3** exhibited growing resonances attributed to butterfly dimers^10,25^ (≈85% consumption after 6 h). Under the same conditions, the spectra of **DAH1** and **DAH2** remained unchanged. According to UV–vis spectroscopy (Figures 2b,c and S13), the half-lives τ_1/2_ of **DAH2** and **DAH3** (DCM) are 4 days and 6 h, respectively. Solutions of **DAH1** are unchanged after 7 d under ambient light and atmosphere (Figure 2b); that is, **DAH1** is completely stable. Similar to heptacenes,^11,20,22^ the positions of the TIPS-ethynyl groups determine their stability; adjacent to the central ring they protect the center of the azaacene more effectively than those with more arene rings in between.

We studied the effect of N atom loading on stability. Adding another pyrazine moiety, **TAH** started to decompose after 7 d (*I*_7d_/*I*_0_ = 82% at λ_max_). **HAH** displayed a τ_1/2_ of 5 d; it was spontaneously reduced to its *N,N*′-dihydro species. In contrast to the decay channels of alkynylated acenes, this process is reversible: reoxidation proceeded quantitatively to **HAH**. Reduction of **HAH** was also observed under NMR conditions, and its spectrum was recorded under an inert atmosphere in the presence of PbO_2_. Removing the central pyrazine unit dramatically decreases the stability (τ_1/2 Hep_ = 25 h, endoperoxide formed; see SI, Figures S67 and S68), although this is still reasonably stable for a heptacene, as it is only protected by four substituents compared to the most stable congener with six.^22^ In dilute solution, the rank order of stability is **DAH1** > **TAH** > **HAH** ≈ **DAH2** > **Hep** > **DAH3** and depends upon the number of nitrogen atoms and placement of the TIPS-ethynyl substituents. In comparison to dilute solution, in the solid state, their stability increased with the rank order **DAH1 > DAH2 > TAH > HAH > Hep > DAH3**.

Specimens suitable for single-crystal structure analysis (SCRA) were grown by slow diffusion of methanol into chloroform (**Hep** polymorph I, **DAH1**, and **TAH**) or dichloromethane solutions (**DAH2**) or via cooling concentrated solutions in benzene (**Hep** polymorph II). **Hep** (polymorph II) and **DAH2** crystallize as benzene and dichloromethane solvates, respectively (Table S2).

All of the acene backbones deviate from planarity (Figure 3); most are S-shaped. **DAH2** contains two independent molecules per unit cell, one of which is strongly bent. The backbone of **Hep** (polymorph II) is almost planar. One-dimensional stacks extend along one direction with a mean π–π distance of 339 pm between the overlapping but slightly offset terminal rings. **DAH1** and **TAH** exhibit an overlap of ∼3.5, three rings along the backbone, and columnar stacking with π–π distances of 0.34 Å. The noncentral pyrazine moiety in **TAH** is statistically disordered throughout the crystal lattice. **Hep** (polymorph I) and **DAH2** lack π–π interactions due to their edge-to-face arrangement. **DAH1** did not form a solvate when crystallized from benzene. In **HAH** hydrogen atoms at the central pyrazine rings occupy about 50% of the molecules (see SI, Figure S29) according to SCRA, providing evidence for its spontaneous reduction.

**Figure 3:**
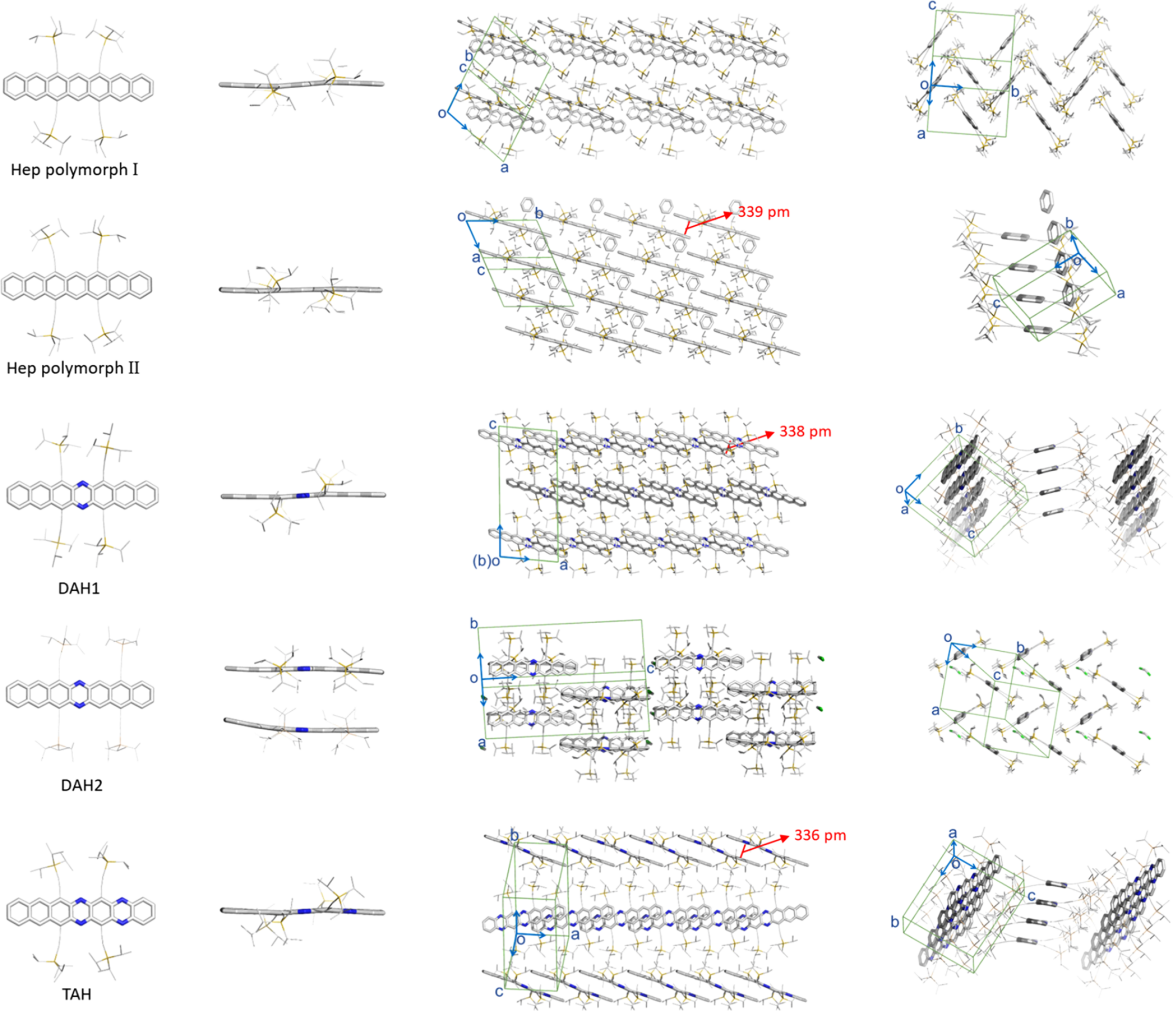
Solid state structures (representative depictions) and packing motif obtained for (aza)heptacenes; hydrogen atoms are omitted for clarity. Note that for **TAH**, the position of the noncentral pyrazine is statistically disordered due to the centrosymmetry of the lattice. π-Stacking distances are estimated from the mean distances of the (aza)acene backbones. Corresponding atomic colors: carbon, gray; nitrogen, blue; silicon, yellow; chlorine, green.

The transfer integrals of all possible pairs of neighboring molecules (SCRA, ADF software package)^45^ and reorganization energies (four-point method, Gaussian16^35^) were used to compute the electron and hole transport mobilities μ (Table S3). Only the highest transfer integral of each compound is shown; all other transfer integrals, including images of the dimer pairs, are listed in the SI (Tables S4–S6, Figure S69). In comparison to hexaethynylheptacene,^22^**Hep** displays higher calculated mobilities: a smaller number of centrally placed TIPS-ethynyl substituents facilitates charge transport. Calculated hole mobilities of 2.6 cm^2^ V^–1^ s^–1^ (comparable to that of **TIPS-Pen**(8)) and electron mobilities of 3.5 cm^2^ V^–1^ s^–1^, suggest **DAH1** is an attractive ambipolar semiconductor. **TAH** might be a promising n-type transporting material with theoretical electron mobilities up to 4.6 cm^2^ V^–1^ s^–1^, surpassing **TIPS-TAP**.^46^ These calculated mobilities are based on a diffusion model, which is valid only for crystal structures with a single molecule per unit cell of perfect translational symmetry and neglects the contribution of lattice phonons. These calculations provide the upper limit of charge carrier mobilities in the packing analyzed.^47^

**DAH1** and **TAH** transport electrons in bottom gate/top contact field-effect transistors (Figure S70) with the best electron mobilities at 0.042 and 0.0031 cm^2^ V^–1^ s^–1^ (Table S7). **Hep** and **DAH2** are ambipolar transport materials with μ_n-max_ = 0.023 cm^2^ V^–1^ s^–1^ and μ_p-max_ = 0.038 cm^2^ V^–1^ s^–1^ for **Hep** (**DAH2**: μ_n-max_ = 0.005 cm^2^ V^–1^ s^–1^ and μ_p-max_ = 0.0017 cm^2^ V^–1^ s^–1^). The discrepancies between the calculated and experimental values are due to the quality of the thin films and also the limitations of the calculations. Compared to hexaethynyl-heptacene,^22^ the mobility of **Hep** is 30 and 22 times higher for the n-channel and the p-channel transport, respectively. We expect the experimental mobilities of **Hep**, **DAH1**, and **TAH** to improve through device optimization. Thin film XRD analysis (see SI, Section 16) of **Hep** (polymorph I) and **DAH2** suggests that the order in thin films and in the single crystal are identical. For **DAH1** and **TAH** the diffraction patterns do not match the simulated diffractograms (single crystal), indicating that the molecules pack differently in thin films.

In conclusion, we have prepared stable and isolable azaheptacenes, claimed ≈100 years ago.^23^ The substitution pattern of **DAH1** renders it immune to endoperoxide formation, butterfly dimerization, and Diels–Alder reactions. The correct placement of the silylethynyl substituents is critical for the azaheptacenes’ stability with respect to dimerization. In combination with a strategically placed central pyrazine, oxidation is suppressed. In solution, an increasing number of pyrazine units destabilizes azaheptacenes: We observe spontaneous reduction to the dihydro species as the main degradation pathway of these electron-poor systems. **DAH1** and **TAH**, the most stable of any reported azaheptacene, are promising n-type semiconductors according to the calculated mobilities. We will further explore future applications of thin film transistors. The dibromides **6a**,**b** are precursors to azaoctacenes and azanonacenes if they are coupled to diaminonaphthalenes and -anthracenes. We encourage the community to apply azaheptacenes as stability issues are solved.

## Supporting Information Available

Additional synthetic procedures and characterization data of intermediates, additional spectra, X-ray crystallographic data, and details for theoretical calculations (PDF)
Experimental data are accessible via HeiData, an institutional repository from Heidelberg University, under DOI:10.11588/data/P9AVCU.
