# Supplementary material for: Stabilizing Azaheptacenes
Source: J Am Chem Soc. 2024 Feb 27;146(9):5793–8. doi: 10.1021/jacs.3c13629 (PMC10921409; doi:10.1021/jacs.3c13629)
Supplement: Supplementary file 1 — ja3c13629_si_001.pdf [file ja3c13629_si_001.pdf]

Supporting Information

Stabilizing Azaheptacenes

Wansheng Zong, Nikolai Hippchen, Nico Zeitter, Steffen Maier, Philipp Ludwig, Frank Rominger, Jan Freudenberg,* and Uwe H. F. Bunz*

Table of Content

1. General Methods......3

2. Tables......3

3. Synthesis......6

4. Calculations......14

5. IR Spectroscopy......15

6. Mass Spectra......16

7. Cyclic Voltammetry......19

8. Time-dependent NMR Spectra......20

9. UV−Vis Absorption Spectra......22

10. Photos of the As-prepared Compounds......23

11. UPLC Analysis......24

12. Crystal Structures......27

13. NMR Spectra......29

14. Crystallographic Data......49

15. Calculated Charge Carrier Mobilities......61

16. OFET Fabrication and Measurement......63

17.Film Morphology......65

18. References......67

1. General Methods

NMR spectra were recorded at room temperature on BRUKER AVANCE spectrometers using the specified frequency. Chemical shifts (δ) are given in parts per million (ppm) relative to internal solvent signals.[1] IR spectra were recorded from neat powder of the respective analyte on a JASCO FT/IR-4100 spectrometer. High-resolution mass spectra (HRMS) were obtained by (matrix-assisted) laser desorption/ionization (LDI/MALDI) using *trans*-2-[3-(4-*tert*-butylphenyl)-2-methyl-2-propenylidene]malononitrile (DCTB) as matrix, electrospray ionisation (ESI) or direct analysis in real time (DART) experiments on a BRUKER ApexQe hybrid 9.4 T FT-ICR spectrometer or BRUKER Autoflex Speed TOF spectrometer. The GC-MS spectra were recorded on the apparatus 7890 A fabricated by Agilent Technology. Cyclovoltammographic (CV) measurements were performed on a VersaSTAT 3 potentiostat by Princeton Applied Research. Absorption spectra were recorded using a Jasco UV-Vis V-670 spectrophotometer. Melting points (m.p.) were determined in open glass capillaries with a melting point apparatus MELTEMP (Electrothermal, Rochford, UK). The elemental analysis was carried out on Vario MICRO cube (Elementar) to detect the C, H, N elemental content. Ultra Performance Liquid Chromatography (UPLC) was performed on an Acquity Water UPLC-SQD2 using BEH C18 column and IPA-ACN as eluent. Samples for UPLC were prepared by dissolving compounds in THF (0.5 mg/mL). Computational studies were carried out using DFT calculations on Gaussian 16.[2] TMS groups were used instead of TIPS groups to simplify the FMO calculations. First, the gas-phase ground-state equilibrium geometry of the molecules was optimized at the B3LYP/def2-SVP level of theory. Afterwards, the received geometries were refined using the B3LYP/def2-TZVP level of theory. FMO calculations were performed starting from the optimized geometries on the B3LYP/def2-TZVP level of theory. *o*-diamines **2a**,**b**,[3] 2,3-dibromo-1,4-anthraquinone[4], ((2,3-dibromoanthracene-9,10-diyl)bis(ethyne-2,1-diyl))bis(triisopropylsilane) **3b**[5] and 1,2-Dibromo-3,6-diiodo-4,5-*o*-quinone **4**[6] were synthesized according to the respective literature procedure.

2. Tables

Table S1. Total energy of **DAH1***, **DAH2*** and **DAH3*** **(**Gaussian16, B3LYP/def2-SVP, TMS groups replaced TIPS groups to reduce computational costs).

Compd.	Energy (eV)	ΔE (eV) ^{a}
**DAH1***	-85002.892	0.178
**DAH2***	-85003.070	0.033
**DAH3***	-85003.103	0

^{a} The energy of DAH3 was set to 0 as a reference compound.

Table S2. Crystal parameters for (aza)heptacenes.

Compd.	space group	Z	a [Å]	b [Å]	c [Å]	α (deg.)	β (deg.)	γ (deg.)	cell volume [$Å^3 \times 10^3$]	solvate
**Hep** (polymorph I)	$P2_1/c$	2	15.99	15.39	15.04	90.0	113.1	90.0	3.40	—
**Hep** (polymorph II)	$P\bar{1}$	1	9.79	13.69	15.80	67.7	86.6	78.1	1.92	C_6H_6
**DAH1**	$P2_1/n$	2	9.14	17.96	20.63	90.0	95.6	90.0	3.37	—
**DAH2**	$P\bar{1}$	4	15.03	15.17	32.47	79.3	79.2	89.7	7.09	DCM
**TAH**	$P2_1/c$	2	9.49	20.15	17.14	90.0	91.3	90.0	3.28	—

Table S3. Calculated transfer integrals (only highest shown), reorganization energies and mobilities for electron and hole transport.

Entry	transfer integral [meV]		reorganization energy [meV]		μ_{theo} [$cm^2V^{-1}\,s^{-1}$]	
	electron	hole	electron	hole	electron	hole
**Hep** (polymorph I)	2.9	1.2	133.0	104.3	0.014	3.9×10^{-3}
**Hep** (polymorph II)	24.9	10.8	133.0	104.3	0.92	0.25
**DAH1**	77.2	49.7	145.2	102.3	3.5	2.6
**DAH2**	4.2	4.8	142.0	101.6	0.020	0.024
**TAH**	90.1	11.9	152.4	—	4.6	—
Hexaethynyl-heptacene	7.7	0.7	135.2	111.2	0.16	7.2×10^{-3}
**TIPS-Pen**	129.8	23.5	187.5	132.8	3.5	0.26
**TIPS-TAP**	102.5	1.3	194.9	207.4	1.8	4.3×10^{-4}

Table S4. Calculated transfer integrals for electron transport.

Compd.	Dimer 1 [meV]	Dimer 2 [meV]	Dimer 3 [meV]	Dimer 4 [meV]	Dimer 5 [meV]	Dimer 6 [meV]
**Hep** (polymorph I)	2.93	1.68	1.68	2.93	1.68	1.68
**Hep** (polymorph II)	24.87	24.87	0.96	6.45		
**DAH1**	77.22	77.22	0.38	0.38		
**DAH2**	1.03	4.27	0.96	3.52	1.14	2.49
**TAH**	90.06	90.06	1.24	1.24		
Hexaethynyl-heptacene	7.66		2.14			
**TIPS-Pen**	129.78	63.63	7.49	129.78	63.63	7.49
**TIPS-TAP**	43.93	102.53	6.59	43.93	102.53	6.59

Table S5. Calculated transfer integrals for hole transport.

Compd.	Dimer 1 [meV]	Dimer 2 [meV]	Dimer 3 [meV]	Dimer 4 [meV]	Dimer 5 [meV]	Dimer 6 [meV]
**Hep** (polymorph I)	1.17	0.26	0.26	1.17	0.26	0.26
**Hep** (polymorph II)	10.82	10.82	5.03	2.25		
**DAH1**	49.67	49.67	0.86	0.86		
**DAH2**	2.27	5.06	1.29	8.5	4.29	3.42
**TAH**	11.89	11.89	0.16	0.16		
Hexaethynyl-heptacene	0.67	2.41				
**TIPS-Pen**	23.48	2.64	1.56	23.48	2.64	1.56
**TIPS-TAP**	1.26	0.17	0.65	1.26	0.17	0.65

Table S6. Distances between the molecules in the dimer pairs.

Compd.	r1 [Å]	r2 [Å]	r3 [Å]	r4 [Å]	r5 [Å]	r6[Å]
**Hep** (polymorph I)	15.992	10.758	10.758	15.992	10.758	10.758
**Hep** (polymorph II)	13.695	13.695	18.405	9.787		
**DAH1**	9.137	9.137	14.736	14.736		
**DAH2**	17.578	10.703	10.647	10.617	10.658	17.541
**TAH**	9.443	9.443	13.209	13.209		
Hexaethynyl-heptacene	19.302	10.857				
**TIPS-Pen**	7.565	10.212	16.216	7.565	10.212	16.216
**TIPS-TAP**	9.960	7.612	16.023	9.960	7.612	16.023

Table S7. Charge carrier mobilities of drop-casting bg/tc transistors of **Hep**, **DAH1**, **DAH2** and **TAH**.

Compd.	$\mu_{n\text{-max}}$ [$cm^2V^{-1} s^{-1}$]	$\mu_{n\text{-ave}}$ [$cm^2V^{-1} s^{-1}$]	$\mu_{p\text{-max}}$ [$cm^2V^{-1} s^{-1}$]	$\mu_{p\text{-ave}}$ [$cm^2V^{-1} s^{-1}$]	on/off ratios
**Hep**	2.3×10^{-2}	1.2×10^{-2}	3.8×10^{-2}	2.3×10^{-2}	10^{3}
**DAH1**	4.2×10^{-2}	1.7×10^{-2}	—	—	10^{5}
**DAH2**	4.9×10^{-3}	2.7×10^{-3}	1.7×10^{-3}	5.7×10^{-4}	10^{3}-10^{4}
**TAH**	3.1×10^{-3}	1.5×10^{-3}	—	—	10^{4}
Hexaethynyl-heptacene[7]	1.4×10^{-3}	7.7×10^{-4}	1.7×10^{-3}	1.1×10^{-3}	—
**TIPS-Pen**[8]	—	—	1.8	0.65	—
**TIPS-TAP**[9]	11.0	7.6	—	—	—

3. Synthesis

GP1: Cyclocondensation reaction

The freshly prepared *o*-quinone (1.20 eq.) and the *o*-diamine (1.00 eq.) were dissolved in DCM/acetic acid (1:1) and stirred at room temperature until TLC monitoring indicated complete reaction. After evaporation of the solvent, the crude product was purified by column chromatography (PE/DCM, 10:1).

GP2: Sonogashira reaction

Under inert conditions a mixture of **10a** or **10b** (1.00 eq.), CuI (0.5 eq.), $(PPh_3)_2PdCl_2$ (0.20 eq.) and TIPS acetylene (5.00 eq. for **11a**; 10.0 eq. for **11b**) were dissolved in degassed THF/triethylamine (1:1). The reaction mixture was stirred at room temperature (for **11a**) or 50 °C (for **11b**) for 36 h. After cooling to room temperature, the mixture was purified by column chromatography (PE→PE/DCM, 30:1). The resulting mixture of dihydro compounds were dissolved in DCM and oxidized with PbO_2 (20 eq.) for 1 h. After filtering through Celite and solvent removal under vacuum, the crude product was purified by column chromatography (PE→PE/DCM, 10:1).

GP3: Buchwald-Hartwig coupling

The dibromides (1.00 eq.), diamines (1.50 eq.), RuPhosPd G1 (ethyl *t*-butyl ether adduct) (0.50 eq.) P(*t*-Bu)$_3$HBF$_4$ (0.50 eq.) and Cs_2CO_3 (4.00 eq.) were dissolved in dry, degassed toluene in a flame-dried flask under nitrogen. The mixture was heated to 100 °C for 6 h. After cooling to room temperature, the mixture was filtrated over silica, the solvent was removed and the residue was purified by column chromatography (PE/DCM, 5:1).

GP4: Oxidation reaction of *N,N'*-dihydro- azaheptacenes

An excess of MnO_2 (100 eq.) was added to solutions of *N,N'*-dihydro- azaheptacenes (1.00 eq.) in DCM (10 mL) and stirred at room temperature for 5 min (**DAH** and **TAH**) or 30 min (**HAH**). The solvent was removed and the residue was purified by column chromatography (PE/DCM, 5:1→1:1). To avoid decomposition, a small amount of methanol was added to the fractions to precipitate the products after separation. Afterwards, the solvent was evaporated as fast as possible in a schlenk line under nitrogen atmosphere to furnish microcrystalline target products.

[(2,3-Dibromoanthracene-1,4-diyl)diethyne-2,1-diyl]bis(tripropan-2-ylsilane). (3a)

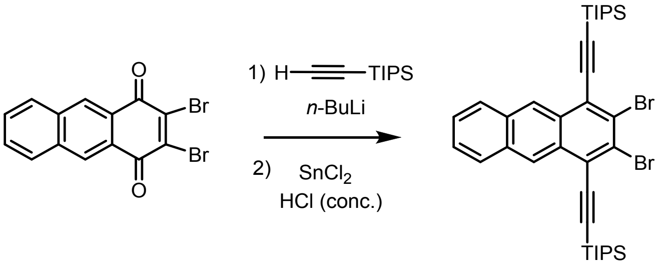

3a

Scheme S1. Synthesis of [(2,3-dibromoanthracene-1,4-diyl)diethyne-2,1-diyl]bis(tripropan-2-ylsilane) **3a**.

Under an inert atmosphere, tri-*iso*-propylsilyl acetylene (1.49 g, 8.20 mmol, 3.00 eq.) was dissolved in dry THF (20 mL) at 0°C and treated with *n*-BuLi (3.28 mL, 2.50 M in hexane, 8.20 mmol, 3.00 eq.). After stirring for 1.5 h at room

temperature, the 2,3-dibromo-1,4-anthraquinone (1.00 g, 2.73 mmol, 1.00 eq.) was added portionwise to form a light brown solution, which was stirred for 20 h. The reaction was quenched with aqueous ammonium chloride (50 mL), followed by extraction with diethyl ether (3×50 mL). The combined organic layer was washed with water and brine and dried over $MgSO_4$. Evaporation under reduced pressure gave the crude diol which was filtered through a plug of silica using petroleum ether and diethyl ether subsequently to separate it from excessive acetylene. After evaporation of the solvents, the intermediate diol was diluted in THF (20 mL) and a saturated solution of $SnCl_2$ in conc. hydrochloric acid (7 mL) was added to form a yellow reaction mixture, which was stirred for 2.5 h. At this point, the product was extracted with diethyl ether (3 x 50 mL) and the combined organic layer was washed with water (30 mL), 1 M aqueous sodium hydroxide (30 mL) and brine (30 mL). After drying over $MgSO_4$, the solvent was evaporated under reduced pressure. Flash column chromatography (PE/DCM, 10:1) yielding **3a** as a blocky crystalline solid (670 mg, 0.96 mmol, 34 %). ^{1}H NMR (500 MHz, $CDCl_3$, r.t.) δ = 8.99 (s, 2H), 8.04 (dd, *J* = 6.4, 3.3 Hz, 2H), 7.55 (dd, *J* = 6.4, 3.2 Hz, 2H), 1.31-1.26 (m, 42H) ppm. ^{13}C NMR (126 MHz, $CDCl_3$, r.t.) δ = 132.8, 130.2, 128.7, 127.0, 126.6, 126.3, 124.9, 105.5, 104.1, 19.0, 18.9, 11.6 ppm. m.p. >350 °C (decomp.). IR: ν = 2939, 2862, 1518, 1459, 1359, 1289, 1063, 995, 881, 820, 765, 660 cm^{-1}. HR-MS(MALDI+): *m/z* [M]$^+$ calcd for $C_{36}H_{48}Br_2Si_2$ 694.1656; found 694.1642.

6,8,15,17-Tetrakis((triisopropylsilyl)ethynyl)-7,16-dihydrodinaphtho[2,3-b:2',3'-i]phenazine. (DAH1-H$_2$)

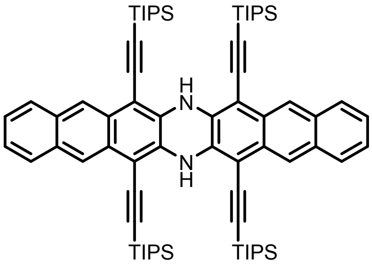

GP3 was applied to **2a** (61.2 mg, 107 µmol), **3a** (50.0 mg, 71.8 µmol), RuPhosPd G1 (29.3 mg, 35.9 µmol) and Cs_2CO_3 (93.5 mg, 287 µmol) yielding **DAH1-H$_2$** (49.9 mg, 45.2 µmol, 63%) as a yellow flaky crystalline solid. ^{1}H NMR (600 MHz, $CDCl_3$, r.t.) δ = 8.56 (s, 4H), 7.82 (dd, *J* = 4.2, 2.2 Hz, 4H), 7.76 (s, 2H), 7.38 (dd, *J* = 4.3, 2.1 Hz, 4H), 1.30-1.16 (m, 84H) ppm. ^{13}C{^{1}H} NMR (151 MHz, $CDCl_3$) δ = 131.9, 131.8, 129.2, 128.0, 125.5, 123.7, 106.6, 100.9, 99.4, 19.3, 12.4 ppm. m.p. >350 °C (decomp.). IR: ν = 3434, 2941, 2864, 1740, 1460, 1371, 1223, 880, 831, 740, 669 cm^{-1}. HR-MS(MALDI+): *m/z* [M+H]$^+$ calcd for $C_{72}H_{99}N_2Si_4$ 1103.6880; found 1103.6843. R_f = 0.32 (SiO_2, petroleum ether/DCM 5:1, v/v).

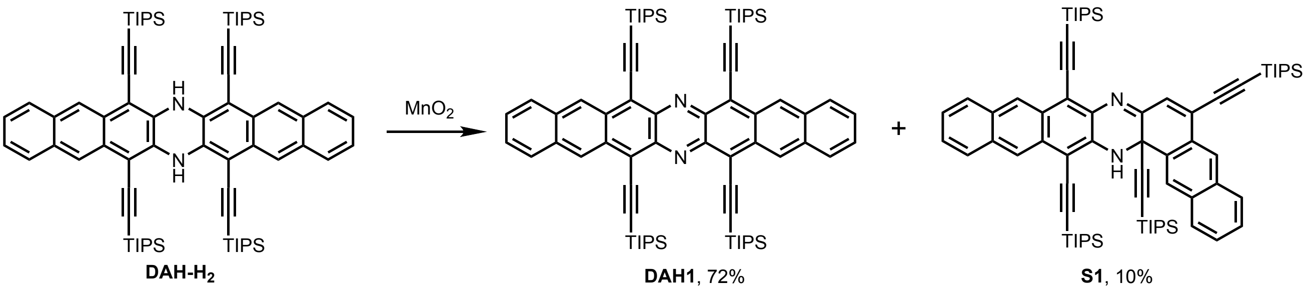

Scheme S2. Synthesis of **DAH1** and the by-product **S1**.

6,8,15,17-Tetrakis((triisopropylsilyl)ethynyl)dinaphtho[2,3-b:2',3'-i]phenazine. (DAH1)

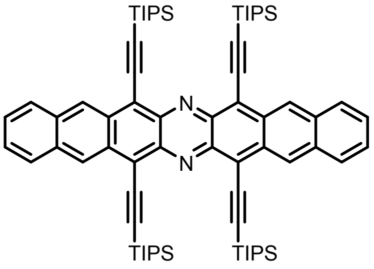

GP4 was applied to **DAH1-H$_2$** (20.0 mg, 18.1 µmol) and MnO_2 (157 mg, 1.81 mmol) yielding **DAH1** (14.3 mg, 13.0 µmol, 72%) as a dark green microcrystalline solid. ^{1}H NMR (600 MHz, $CDCl_3$, r.t.) δ = 9.35 (s, 4H), 7.89 (dd, *J* = 6.6, 3.2 Hz, 4H), 7.41 (dd, *J* = 5.9, 2.8 Hz, 4H), 1.49-0.80 (m, 84H). ^{13}C{^{1}H} NMR (151 MHz, $CDCl_3$, r.t.) δ = 141.0, 134.6, 133.7, 128.9, 127.6, 127.2, 120.9, 111.9, 105.2, 19.3, 12.2 ppm. m.p. >350 °C (decomp.). IR: $\boldsymbol{\nu}$ = 2937, 2862, 1740, 1462, 1359, 1231, 1057, 994, 880, 821, 762, 661 cm^{-1}. HR-MS(MALDI+): *m/z* [M]$^+$ calcd for $C_{72}H_{96}N_2Si_4$ 1100.6645; found 1100.6637. R_f = 0.52 (SiO_2, petroleum ether/DCM 1:1, v/v). Element analysis calcd. (%) for $C_{72}H_{96}N_2Si_4$: C 78.48, H 8.78, N 2.54; found C 78.11, H 8.79, N 2.53.

6,9,16,17a-Tetrakis((triisopropylsilyl)ethynyl)-17,17a-dihydrodinaphtho[2,3-a:2',3'-i]phenazine. (S1)

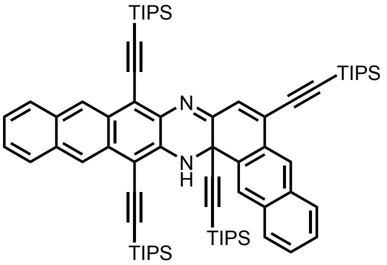

S1 was obtained as a by-product of the oxidation reaction of **DAH1-H$_2$**: An excess of MnO_2 (100 eq., 157 mg, 1.81 mmol) was added to a solution of **DAH1-H$_2$** (20.0 mg, 18.1 µmol) in DCM (5.00 mL) and stirred at room temperature for 10 min. The solvent was removed under vacuum and the residue was purified by column chromatography (PE/DCM, 5:1→1:1) yielding **S1** (1.98 mg, 1.80 µmol, 10%) as a brown powderous solid. ^{1}H NMR (600 MHz, $CDCl_3$, r.t.) δ = 9.01 (s, 1H), 8.67 (s, 1H), 8.46 (s, 1H), 8.08 (s, 1H), 7.98 (d, *J* = 8.2 Hz, 1H), 7.94 (d, *J* = 8.3 Hz, 1H), 7.90 (d, *J* = 7.2 Hz, 1H), 7.83 (d, *J* = 7.5 Hz, 1H), 7.64-7.53 (m, 2H), 7.50-7.46 (m, 1H), 7.46-7.40 (m, 1H), 7.08 (s, 1H) ppm. ^{13}C NMR (126 MHz, $CDCl_3$, r.t.) δ = 158.6, 138.8, 137.7, 134.3, 133.7, 133.2, 133.1, 131.8, 131.1, 131.0, 129.6, 129.0, 129.0, 128.7, 128.2, 128.1, 127.9, 127.7, 127.4, 127.3, 127.2, 126.4, 125.1, 124.8, 123.2, 120.0, 106.6, 104.2, 103.5, 102.8, 102.3, 102.2, 101.8, 86.9, 50.1, 19.3, 19.3, 19.1, 19.0, 18.4, 18.4, 11.8, 11.8, 11.6, 11.0 ppm. m.p. >350 °C (decomp.). IR: $\boldsymbol{\nu}$ = 2941, 2864, 2360, 2341, 1457, 1379, 1110, 1050, 881, 732, 669, 457 cm^{-1}. HR-MS(MALDI+): *m/z* [M+H]$^+$ calcd for $C_{72}H_{99}N_2Si_4$ 1103.6880; found 1103.6829.

5,8,15,18-Tetrakis((triisopropylsilyl)ethynyl)-7,16-dihydrodinaphtho[2,3-b:2',3'-i]phenazine. (DAH2-H$_2$)

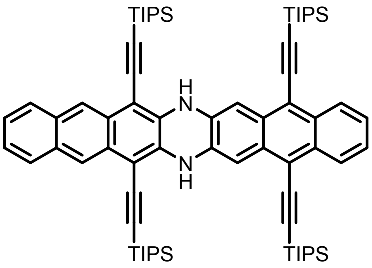

GP3 was applied to **2a** (49.0 mg, 86.1 µmol), **3b** (40.0 mg, 57.4 µmol), RuPhosPd G1 (23.4 mg, 28.7 µmol) and Cs_2CO_3 (74.8 mg, 229 µmol) yielding **DAH2-H$_2$** (46.9 mg, 42.5 µmol, 74%) as an orange flaky crystalline solid. ^{1}H NMR (600 MHz, $CDCl_3$, r.t.) δ = 8.47 (m, 4H), 7.84 (dd, *J* = 3.8, 1.6 Hz, 2H), 7.48 (dd, *J* = 3.8, 1.7 Hz, 2H), 7.47 (s, 2H), 7.39 (dd, *J* = 3.7, 1.8 Hz), 7.29 (s, 2H), 1.42-1.14 (m, 84H) ppm. ^{13}C{^{1}H} NMR (151 MHz, $CDCl_3$) δ = 133.3, 132.2, 131.8, 131.3, 131.2, 128.4, 127.9, 126.8, 126.2, 125.4, 123.1, 115.4, 105.8, 105.6, 104.3, 103.7, 100.2, 98.3, 18.8, 11.7, 11.6 ppm. m.p. >350 °C (decomp.). IR: $\boldsymbol{\nu}$ = 3369, 2943, 2865, 1462, 1436, 1394, 1361, 1056, 1013, 991, 876, 761, 661 cm^{-1}. HR-MS(MALDI+): *m/z* [M+H]$^+$ calcd for $C_{72}H_{99}N_2Si_4$ 1103.6880; found 1103.6856. R_f = 0.33 (SiO_2, petroleum ether/DCM 5:1, v/v).

5,8,15,18-Tetrakis((triisopropylsilyl)ethynyl)dinaphtho[2,3-b:2',3'-i]phenazine. (DAH2)

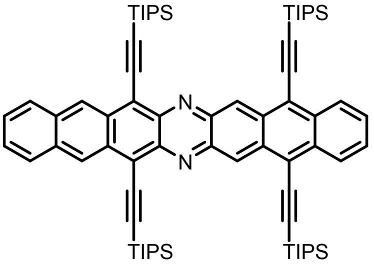

GP4 was applied to **DAH2-H$_2$** (20.0 mg, 18.1 µmol) and MnO_2 (157 mg, 1.81 mmol) yielding **DAH2** (16.9 mg, 15.3 µmol, 85%) as a dark green microcrystalline solid. ^{1}H

NMR (600 MHz, $CDCl_3$, r.t.) δ = 9.69 (s, 2H), 9.34 (s, 2H), 8.58 (dd, *J* = 4.5, 2.1 Hz, 2H), 7.93 (dd, *J* = 4.3, 2.1 Hz, 2H), 7.51 (dd, *J* = 4.6, 2.0 Hz, 2H), 7.43 (dd, *J* = 4.5, 2.0 Hz, 2H), 1.46-1.16 (m, 84H) ppm. $^{13}C\{^1H\}$ NMR (151 MHz, $CDCl_3$) δ = 142.4, 141.4, 134.4, 134.0, 133.5, 133.3, 129.1, 128.8, 127.8, 127.7, 127.3, 127.1, 120.9, 119.5, 110.9, 107.9, 104.5, 103.5, 19.1, 19.0, 11.7, 11.6 ppm. m.p. >350 °C (decomp.). IR: $\boldsymbol{\nu}$ = 2942, 2861, 1462, 1392, 1362, 1265, 1078, 1047, 1015, 878, 801, 747, 663 cm^{-1}. HR-MS(MALDI+): *m/z* $[M]^+$ calcd for $C_{72}H_{96}N_2Si_4$ 1100.6645; found 1100.6657. R_f = 0.56 (SiO_2, petroleum ether/DCM 1:1, v/v). Element analysis calcd. (%) for $C_{72}H_{96}N_2Si_4$: C 78.48, H 8.78, N 2.54; found C 78.29, H 8.80, N 2.54.

2,3-Dibromo-1,4-diiodo-6,13-bis((triisopropylsilyl)ethynyl)naphtho[2,3-b]phenazine. (5a)

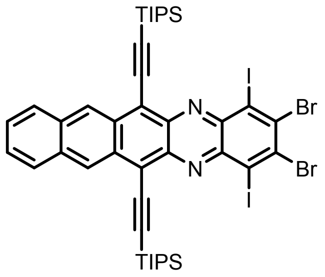

GP1 was applied to **2a** (1.00 g, 1.76 mmol) and **4**[6] (1.09 g, 2.11 mmol) in DCM/acetic acid (25 mL, 1:1) yielding **5a** (444 mg, 423 µmol, 24 %) as a blue powderous solid. ^1H NMR (600 MHz, $CDCl_3$, r.t.) δ = 9.50 (s, 2H), 8.08-7.92 (m, 2H), 7.62-7.40 (m, 2H), 1.46-1.14 (m, 42H) ppm. $^{13}C\{^1H\}$ NMR (151 MHz, $CDCl_3$, r.t.) δ = 142.2, 141.7, 136.2, 133.8, 133.6, 128.9, 127.6, 127.5, 120.9, 114.4, 111.2, 103.5, 19.3, 11.9 ppm. m.p. >350 °C (decomp.). IR: $\boldsymbol{\nu}$ = 2938, 2861, 1459, 1371, 1337, 1105, 1016, 879, 760, 668 cm^{-1}. HR-MS(MALDI+): *m/z* $[M+H]^+$ calcd for $C_{42}H_{49}Br_2I_2N_2Si_2$ 1052.9792; found 1052.9791. R_f = 0.35 (SiO_2, petroleum ether/DCM 5:1, v/v).

2,3-Dibromo-1,4-diiodo-6,13-bis((triisopropylsilyl)ethynyl)quinoxalino[2,3-b]phenazine. (5b)

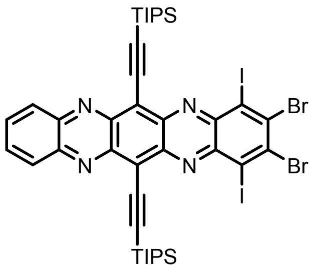

GP1 was applied to **2b** (800 mg, 1.40 mmol) and **4**[6] (870 mg, 1.68 mmol) in DCM/acetic acid (20 mL, 1:1) yielding **5b** (485 mg, 462 µmol, 33 %) as a green powderous solid. ^1H NMR (500 MHz, $CDCl_3$, r.t.) δ = 8.20 (dd, *J* = 7.0, 3.4 Hz, 2H), 7.87 (dd, *J* = 7.0, 3.3 Hz, 2H), 1.44-1.22 (m, 42H) ppm. $^{13}C\{^1H\}$ NMR (126 MHz, $CDCl_3$, r.t.) δ = 146.0, 144.2, 143.6, 142.8, 137.3, 132.9, 130.7, 123.1, 114.5, 114.3, 103.0, 19.3, 11.9 ppm. m.p. >350 °C (decomp.). IR: $\boldsymbol{\nu}$ = 2937, 2860, 1525, 1438, 1380, 1336, 1115, 1024, 881, 750, 672 cm^{-1}. HR-MS(MALDI+): *m/z* $[M+H]^+$ calcd for $C_{40}H_{47}Br_2I_2N_4Si_2$: 1054.9941; found: 1054.9941. R_f = 0.32 (SiO_2, petroleum ether/DCM 5:1, v/v).

2,3-Dibromo-1,4,6,13-tetrakis((triisopropylsilyl)ethynyl)naphtho[2,3-b]phenazine. (6a)

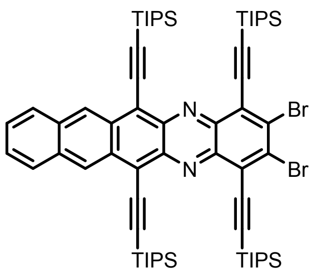

GP2 was applied to **5a** (430 mg, 409 µmol), CuI (39.0 mg, 205 µmol), $(PPh_3)_2PdCl_2$ (57.4 mg, 81.8 µmol) and TIPS acetylene (373 mg, 2.05 mmol) in THF/triethylamine (12 mL, 1:1 v/v) yielding **6a** (186 mg, 159 µmol, 39 %) as a green powderous solid. ^1H NMR (500 MHz, $CDCl_3$, r.t.) δ = 9.49 (s, 2H), 7.99 (dd, *J* = 6.6, 3.2 Hz, 2H), 7.65-7.37 (m, 2H) 1.42-1.17 (m, 84H) ppm. $^{13}C\{^1H\}$ NMR (126 MHz, $CDCl_3$, r.t.) δ = 142.0, 139.4, 134.9, 133.9, 133.4, 128.8, 127.4, 127.2, 126.5, 121.2, 111.3, 109.1, 104.4, 103.9, 19.2, 19.1, 12.1, 11.9 ppm. m.p. >350 °C (decomp.). IR: $\boldsymbol{\nu}$ = 2941, 2864, 2359, 2333, 1461, 1371, 1110, 1077, 1014, 882, 842, 765 cm^{-1}. HR-MS(MALDI+): *m/z* $[M+H]^+$ calcd for $C_{64}H_{91}Br_2N_2Si_4$ 1160.4727; found: 1160.4706. R_f = 0.40 (SiO_2, petroleum ether/DCM 10:1, v/v).

2,3-Dibromo-1,4,6,13-tetrakis((triisopropylsilyl)ethynyl)quinoxalino[2,3-b]phenazine. (6b)

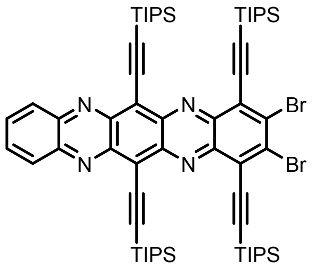

GP2 was applied to **5b** (460 mg, 437 µmol), CuI (41.5 mg, 218 µmol), $(PPh_3)_2PdCl_2$ (61.3 mg, 87.4 µmol) and TIPS acetylene (797 mg, 4.37 mmol) in THF/triethylamine (13 mL, 1:1) yielding **6b** (381 mg, 328 µmol, 75 %) as a green powderous solid. ^{1}H NMR (500 MHz, $CDCl_3$, r.t.) δ = 8.20 (dd, *J* = 6.9, 3.4 Hz, 2H), 7.83 (dd, *J* = 7.1, 3.4 Hz, 2H), 1.36-1.16 (m, 84H) ppm. ^{13}C{^{1}H} NMR (126 MHz, $CDCl_3$, r.t.) δ = 145.7, 144.3, 142.6, 141.3, 136.0, 132.4, 130.5, 126.6, 123.4, 115.1, 109.7, 103.7, 103.6, 19.2, 19.1, 12.1, 11.9 ppm. m.p. >350 °C (decomp.). IR: $\boldsymbol{\nu}$ = 2941, 2864, 2360, 2340, 1444, 1275, 1080, 1016, 882, 842, 764, 671 cm^{-1}. HR-MS(MALDI+): *m/z* $[M+H]^+$ calcd for $C_{62}H_{89}Br_2N_4Si_4$ 1162.4732; found 1162.4760. R_f = 0.38 (SiO_2, petroleum ether/DCM 10:1, v/v).

6,8,15,17-Tetrakis((triisopropylsilyl)ethynyl)-7,16-dihydronaphtho[2,3-b]quinoxalino[2,3-i]phenazine. (TAH-H$_2$)

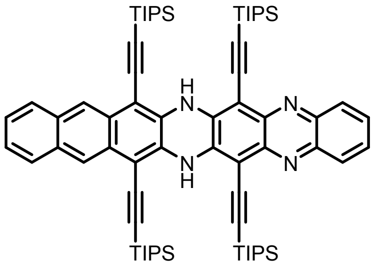

GP3 was applied to **6a** (180 mg, 155 µmol), *o*-phenylenediamine (25.1 mg, 233 µmol), RuPhosPd G1 (63.4 mg, 77.6 µmol) $P(t\text{-}Bu)_3HBF_4$ (22.5 mg, 77.6 µmol) and Cs_2CO_3 (202 mg, 621 µmol) yielding **TAH-H$_2$** (32.6 mg, 29.5 µmol, 19 %) as an orange-red flaky crystalline. ^{1}H NMR (600 MHz, $CDCl_3$, r.t.) δ = 8.64 (s, 2H), 8.06 (s, 2H), 8.02 (dd, *J* = 4.3, 2.3 Hz, 2H), 7.84 (dd, *J* = 4.3, 2.2 Hz, 2H), 7.63 (dd, *J* = 4.4, 2.2 Hz, 2H), 7.42 (dd, *J* = 4.2, 2.0 Hz, 2H) 1.43-1.08 (m, 84H) ppm. ^{13}C{^{1}H} NMR (151 MHz, $CDCl_3$, r.t.) δ = 143.5, 142.6, 135.3, 132.0, 130.5, 129.1, 129.1, 129.0, 128.0, 125.8, 124.1, 107.9, 107.6, 100.9, 100.2, 100.0, 99.3, 19.2, 19.1, 12.2 ppm. m.p. >250 °C (decomp.). IR: $\boldsymbol{\nu}$ = 3356, 2938, 2862, 2359, 2134, 1573, 1449, 1363, 1255, 1056, 852, 757, 671 cm^{-1}. HR-MS(ESI+): *m/z* $[M]^+$ calcd for $C_{70}H_{96}N_4Si_4$ 1104.6707; found 1104.6709. R_f = 0.42 (SiO_2, petroleum ether/DCM 2:1, v/v).

6,8,15,17-Tetrakis((triisopropylsilyl)ethynyl)naphtho[2,3-b]quinoxalino[2,3-i]phenazine. (TAH)

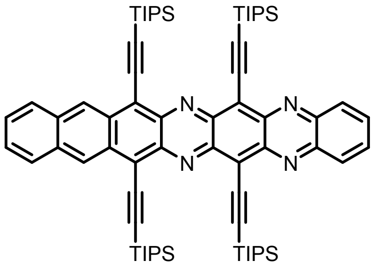

GP4 was applied to **TAH-H$_2$** (10.0 mg, 9.03 µmol) and MnO_2 (78.5 mg, 903 µmol) yielding **TAH** (8.95 mg, 8.12 µmol, 90%) as a dark brown microcrystalline solid. ^{1}H NMR (600 MHz, $CDCl_3$, r.t.) δ = 9.37 (s, 2H), 8.13 (dd, *J* = 4.6, 2.2 Hz, 2H), 7.89 (dd, *J* = 4.6, 2.0 Hz, 2H), 7.76 (dd, *J* = 4.7, 2.1 Hz, 2H), 7.42 (dd, *J* = 4.6, 2.1 Hz, 2H) 1.43-1.05 (m, 84H) ppm. ^{13}C{^{1}H} NMR (151 MHz, $CDCl_3$, r.t.) δ = 146.3, 145.3, 142.6, 141.5, 135.2, 134.3, 133.7, 133.0, 130.6, 129.0, 128.0, 123.3, 121.6, 116.3, 113.2, 105.0, 104.9, 30.1, 19.3, 12.5 ppm. m.p. >130 °C (decomposed into dark red solid with a red shine). IR: $\boldsymbol{\nu}$ = 2938, 2860, 2127, 1533, 1462, 1384, 1264, 1043, 883, 785, 659 cm^{-1}. HR-MS(ESI+): *m/z* $[M]^+$ calcd for $C_{70}H_{94}N_4Si_4$ 1102.6552; found 1102.6550. R_f = 0.48 (SiO_2, petroleum ether/DCM 1:1, v/v). Element analysis calcd. (%) for $C_{70}H_{94}N_4Si_4$: C 76.16, H 8.58, N 5.08; found C 75.97, H 8.61, N 5.05.

6,8,15,17-Tetrakis((triisopropylsilyl)ethynyl)-7,16-dihydropyrazino[2,3-b:5,6-b']diphenazine. (HAH-H$_2$)

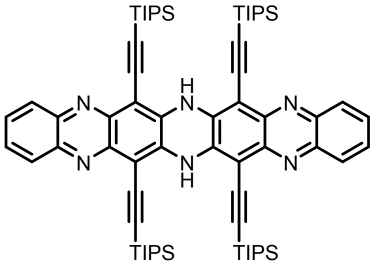

GP3 was applied to **6b** (355 mg, 305 µmol), *o*-phenylenediamine (49.6 mg, 457 mol), RuPhosPd G1 (125 mg, 153 µmol) $P(t\text{-}Bu)_3HBF_4$ (44.3 mg, 153 µmol) and Cs_2CO_3 (397

mg, 1.22 mmol) yielding **HAH-H$_2$** (54.1 mg, 48.9 µmol, 16 %) as a red flaky crystalline. ^{1}H NMR (500 MHz, $CDCl_3$, r.t.) δ = 8.30 (s, 2H), 8.05 (dd, *J* = 6.6, 3.4 Hz, 2H), 7.67 (dd, *J* = 6.7, 3.4 Hz, 2H), 1.35-1.21 (m, 84H) ppm. ^{13}C{^{1}H} NMR (126 MHz, $CDCl_3$, r.t.) δ = 143.3, 142.9, 134.2, 129.6, 129.4, 109.2, 101.9, 98.7, 19.3, 12.3 ppm. m.p. >350 °C (decomp.). IR: $\boldsymbol{\nu}$ = 3345, 2941, 2864, 2360, 1582, 1476, 1433, 1275, 1210, 1061, 883, 750 cm^{-1}. HR-MS(ESI+): *m/z* $[M+H]^+$ calcd for $C_{68}H_{95}N_6Si_4$ 1107.6690; found 1107.6690. R_f = 0.45 (SiO_2, petroleum ether/DCM 2:1, v/v).

6,8,15,17-Tetrakis((triisopropylsilyl)ethynyl)pyrazino[2,3-b:5,6-b']diphenazine. (HAH)

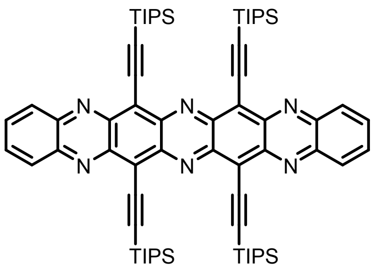

GP4 was applied to **HAH-H$_2$** (10.0 mg, 9.03 µmol) and MnO_2 (78.5 mg, 903 µmol) yielding **HAH** (3.99 mg, 3.61 µmol, 40%) as a dark brown microcrystalline solid. ^{1}H NMR (600 MHz, $CDCl_3$, r.t.) δ = 8.13 (dd, *J* = 6.9, 3.3 Hz, 2H), 7.78 (dd, *J* = 7.0, 3.3 Hz, 2H), 1.37-1.21 (m, 84H) ppm. ^{13}C{^{1}H} NMR (151 MHz, $CDCl_3$, r.t.) δ = 146.0, 145.1, 142.6, 132.5, 130.4, 123.4, 118.0, 104.3, 19.1, 12.0 ppm. m.p. >130 °C (decomposed into dark red solid with a red shine). IR: $\boldsymbol{\nu}$ = 2940, 2861, 2369, 1524, 1456, 1381, 1275, 1111, 1015, 880, 747, 672 cm^{-1}. HR-MS(ESI+): *m/z* $[M+H]^+$ calcd for $C_{68}H_{93}N_6Si_4$ 1105.6535; found 1105.6533. R_f = 0.48 (SiO_2, petroleum ether/DCM 1:1, v/v). Element analysis calcd. (%) for $C_{68}H_{92}N_6Si_4$: C 73.86, H 8.39, N 7.60; found C 73.62, H 8.50, N 7.56.

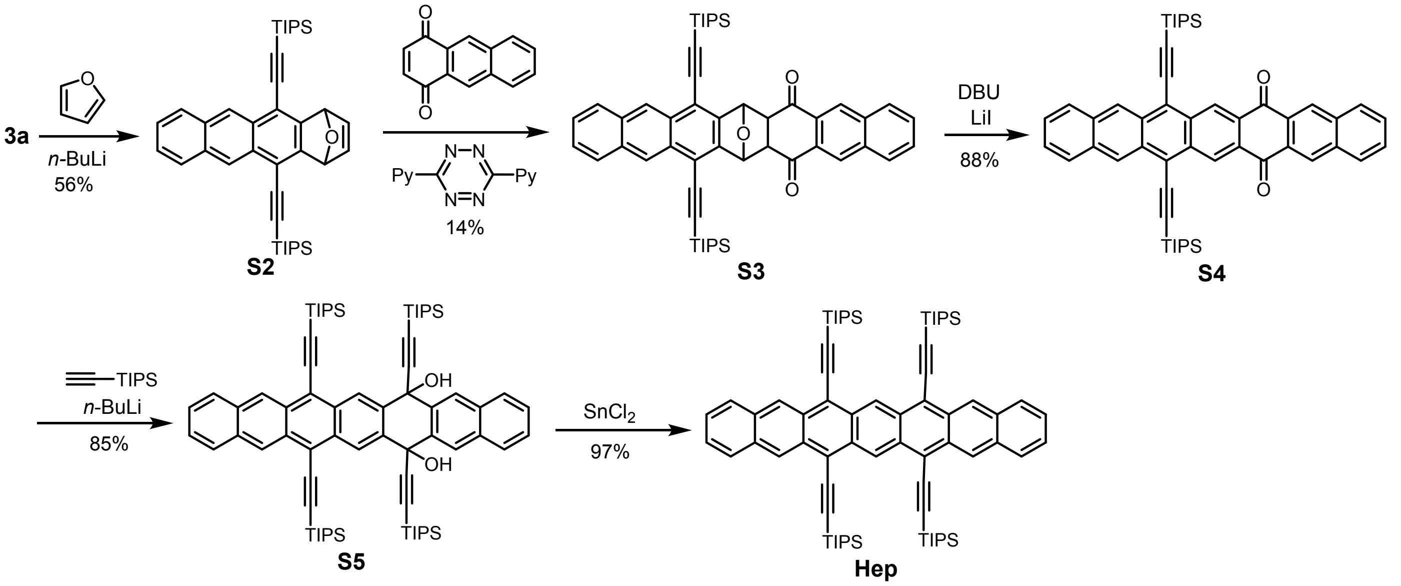

Scheme S3. Synthesis of 6,8,15,17-Tetrakis((triisopropylsilyl)ethynyl)heptacene (**Hep**).

Hep was synthesized according to literature.[10]

5,12-Bis((triisopropylsilyl)ethynyl)-1,4-dihydro-1,4-epoxytetracene (S2)[10]

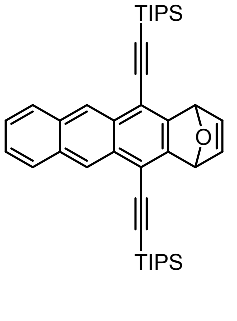

3a (14.3 g, 20.5 mmol, 1.00 eq.) was dissolved in toluene (400 mL) and furan (14.9 mL, 205 mmol, 10.0 eq.) was added under argon atmosphere. The mixture was cooled to 0 °C and *n*-BuLi (8.62 mL, 21.5 mmol, 1.05 eq., 2.50 M in *n*-hexane) was added dropwise over 60 min. The mixture was

stirred for 5 h at r.t. The reaction was quenched with water (300 mL), and the product was extracted with DCM (3 x 200 mL). The solvent was removed under reduced pressure and the crude product was purified by flash column chromatography (SiO_2, PE:DCM 5:1) yielding **S2** (6.97 g, 11.5 mmol, 56%) as a yellow solid. ^{1}H NMR (600 MHz, $CDCl_3$, r.t.) δ = 8.78 (s, 2H), 7.99 – 7.95 (m, 2H), 7.52 – 7.50 (m, 2H), 7.01 (s, 2H), 6.02 (s, 2H), 1.25 (s, 42H) ppm. ^{13}C{^{1}H} NMR (151 MHz, $CDCl_3$, r.t.) δ = 147.0, 140.9, 132.4, 129.5, 128.4, 126.2, 125.9, 113.1, 101.6, 100.2, 82.0, 19.0, 11.5 ppm. m.p. = 334 °C. IR: $\boldsymbol{\nu}$ = 2940, 2863, 2154, 1457, 1328, 993, 949, 882, 871, 819, 745, 718, 667, 577, 469 cm^{-1}. HR-MS(MALDI+): *m/z* [M]$^+$ calcd for $C_{40}H_{52}O_1Si_2$ 604.3551; found 604.3550. R_f = 0.45 (SiO_2, petroleum ether/DCM 4:1, v/v).

8,15-Bis((triisopropylsilyl)ethynyl)-6a,7,16,16a-tetrahydro-7,16-epoxyheptacene-6,17-dione (S3)[10]

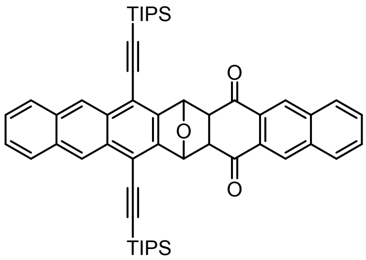

S2 (1.00 g, 1.65 mmol, 1.00 eq.), anthracene-1,4-dione (379 mg, 1.82 mmol, 1.10 eq.) and 3,6-di(pyridin-2-yl)-1,2,4,5-tetrazine (430 mg, 1.82 mmol, 1.10 eq.) were dissolved in dry toluene (100 mL) under argon atmosphere and the solution was stirred at 110 °C for 16 h. Subsequently, the solvent was removed under reduced pressure and the crude product was purified by flash column chromatography (SiO_2, PE:EE 20:1) yielding **S3** (185 mg, 235 µmol, 14%) as a yellow brown solid. ^{1}H NMR (301 MHz, $CDCl_3$, r.t.) δ = 8.36 (s, 2H), 7.89 (s, 2H), 7.67 – 7.58 (m, 2H), 7.36 – 7.26 (m, 4H), 7.08 – 6.91 (m, 2H), 6.08 (dd, J = 3.6, 2.1 Hz, 2H), 3.88 (dd, J = 3.7, 2.1 Hz, 2H), 1.30 (s, 42H) ppm. ^{13}C{^{1}H} NMR (176 MHz, $CDCl_3$, r.t.) δ = 193.0, 141.9, 134.3, 131.8, 130.6, 129.6, 129.1, 128.1, 128.0, 127.2, 126.1, 125.8, 114.5, 103.1, 100.9, 83.5, 51.5, 19.1, 19.0, 11.7 ppm. m.p. 146 °C. IR: $\boldsymbol{\nu}$ = 2940, 2863, 1683, 1455, 1259, 950, 882, 745, 717, 674, 568, 472 cm^{-1}. HR-MS(MALDI+): *m/z* [M]$^+$ calcd for $C_{52}H_{58}O_3Si_2$ 786.3919; found 786.3931. R_f = 0.66 (SiO_2, DCM).

8,15-Bis((triisopropylsilyl)ethynyl)heptacene-6,17-dione (S4) [10]

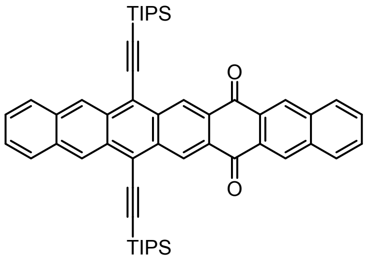

S3 (105 mg, 133 µmol, 1.00 eq.), diazabicycloundecene (406 mg, 0.40 mL, 2.67 mmol, 20.0 eq.) and lithium iodide (35.7 mg, 267 µmol, 2.00 eq.) were dissolved in dry THF (10 mL) under argon atmosphere and the mixture was refluxed overnight. After cooling to room temperature the solvent was removed under reduced pressure and the crude product was purified by flash column chromatography (SiO_2, PE:DCM 1:1) yielding **S4** (90.0 mg, 117 µmol, 88%) as a dark red solid. ^{1}H NMR (600 MHz, $CDCl_3$, r.t.) δ = 9.81 (s, 2H), 9.36 (s, 2H), 9.03 (s, 2H), 8.15 – 8.09 (m, 2H), 8.09 – 8.04 (m, 2H), 7.73 – 7.67 (m, 2H), 7.58 – 7.52 (m, 2H), 1.47 – 1.36 (m, 42H) ppm. ^{13}C{^{1}H} NMR (151 MHz, $CDCl_3$, r.t.) δ = 182.7, 135.5, 133.3, 132.7, 131.5, 131.2, 131.2, 130.4, 130.2, 130.0, 129.6, 128.9, 127.4, 127.2, 122.7, 109.1, 103.0, 19.1, 19.1, 11.8 ppm. m.p. >300 °C. IR: $\boldsymbol{\nu}$ = 2940, 2863, 1681, 1449, 1263, 1201, 997, 879, 753, 735, 713, 673, 582, 471 cm^{-1}. HR-MS(MALDI+): *m/z* [M]$^+$ calcd for $C_{52}H_{56}O_2Si_2$ 768.3814; found 768.3813. R_f = 0.30 (SiO_2, PE:DCM 1:1).

6,8,15,17-Tetrakis((triisopropylsilyl)ethynyl)-6,17-dihydroheptacene-6,17-diol (S5) [10]

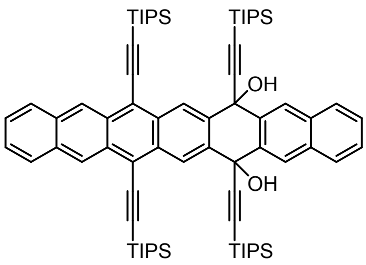

(Triisopropylsilyl)-acetylene (118 mg, 146 µL, 650 µmol, 10.0 eq.) was dissolved in dry *n*-hexane (5.00 mL) under argon atmosphere. *n*-BuLi (247 µL, 9.50 eq., 2.50 M in n-hexane) was added dropwise at r.t. and the mixture was stirred for 60 min. **S4** (50.0 mg, 65.0 µmol, 1.00 eq.) and dry THF (0.20 mL) were added. The suspension was

stirred for 12 h r.t. The reaction was quenched with saturated aqueous ammonium chloride solution and extracted with DCM. The combined organic layers were dried over anhydrous magnesium sulfate and the solvent was removed under reduced pressure. The crude product was purified by flash column chromatography (SiO_2, PE:DCM 2:1) yielding **S5** (63.0 mg, 55.6 µmol, 85%) as a pink solid. ^1H NMR (600 MHz, $CDCl_3$, r.t.) δ = 9.39 (s, 2H), 9.35 (s, 2H), 8.76 (s, 2H), 8.05 – 7.99 (m, 2H), 7.96 – 7.91 (m, 2H), 7.57 – 7.51 (m, 2H), 7.51 – 7.45 (m, 2H), 3.27 (s, 2H), 1.40 – 1.32 (m, 42H), 1.07 – 0.99 (m, 42H) ppm. $^{13}C\{^1H\}$ NMR (151 MHz, $CDCl_3$, r.t.) δ = 137.4, 136.1, 133.5, 132.5, 132.3, 131.0, 128.7, 128.3, 126.9, 126.7, 126.7, 126.3, 126.1, 119.3, 110.4, 107.2, 104.2, 89.6, 69.6, 19.2, 19.1, 18.8, 11.9, 11.5 ppm. m.p. 257 °C. IR: $\boldsymbol{\nu}$ = 2941, 2864, 2350, 1462, 1362, 993, 881, 732, 674, 580, 460 cm^{-1}. HR-MS(MALDI+): *m/z* $[M]^+$ calcd for $C_{74}H_{100}O_2Si_4$ 1132.6811; found 1132.6811. R_f = 0.75 (SiO_2, PE:DCM 1:1).

6,8,15,17-Tetrakis((triisopropylsilyl)ethynyl)heptacene (Hep) [10]

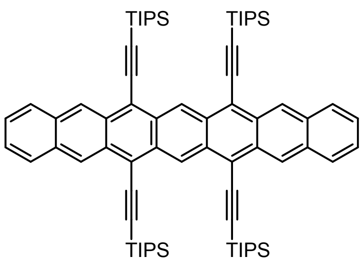

S5 (50.0 mg, 44.1 µmol, 1.00 eq.) was dissolved in a mixture of acetonitrile / THF (3 mL, 1:1) under argon atmosphere. Anhydrous $SnCl_2$ (83.6 mg, 441 µmol, 10.0 eq.) was added, and the reaction mixture was stirred at r.t. overnight. The resulting mixture was filtered, and the precipitate was washed with acetonitrile until the filtrate was clear yielding **Hep** (47.0 mg, 43.0 µmol, 97%) as a brown solid. ^1H NMR (301 MHz, $CDCl_3$, r.t.) δ = 9.88 (s, 2H), 9.26 (s, 4H), 7.95 – 7.80 (m, 4H), 7.41 – 7.32 (m, 4H), 1.39 – 1.30 (m, 84H). The compound was not stable enough for ^{13}C NMR analysis. m.p. 315 °C. IR: $\boldsymbol{\nu}$ = 2941, 2864, 1460, 1259, 1061, 1015, 881, 798, 728, 708, 671, 588, 459 cm^{-1}. HR-MS(MALDI+): *m/z* $[M]^+$ calcd for $C_{74}H_{98}Si_4$ 1098.6740; found 1098.6728. ^{13}C NMR spectra were always contaminated by a degradation product of the heptacene. Element analysis calcd. (%) for $C_{74}H_{98}Si_4$: C 80.81, H 8.98; found C 80.34, H 8.93.

The degradation product of Hep: 6,8,15,17-tetrakis((triisopropylsilyl)ethynyl)-7,16-dihydro-7,16-epidioxyheptacene (S6) [10]

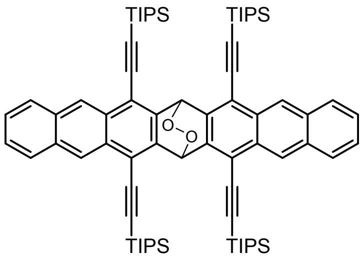

The decomposition product is most likely due to oxidation of the central benzene ring to the endoperoxide. The degradation product was also characterized. ^1H NMR (600 MHz, $CDCl_3$, r.t.) δ = 9.12 (s, 4H), 8.03 – 7.97 (m, 4H), 7.57 – 7.50 (m, 4H), 7.16 (s, 2H), 1.34 – 1.25 (m, 84H) ppm. $^{13}C\{^1H\}$ NMR (151 MHz, $CDCl_3$, r.t.) δ = 134.49, 132.60, 130.94, 128.47, 127.04, 126.68, 117.65, 105.48, 101.77, 76.61, 19.22, 19.11, 12.17.ppm HR-MS(MALDI+): *m/z* $[M]^+$ calcd for $C_{74}H_{98}O_2Si_4$ 1130.6638; found 1130.6616.

4. Calculations

All calculations were performed using Gaussian16. TMS groups were used instead of TIPS groups to simplify calculations. First, the gas-phase ground-state equilibrium geometry of the molecules was optimized at the B3LYP/def2-SVP level of theory. Afterwards, the received geometries were refined using the B3LYP/def2-TZVP level of theory. FMO calculations were performed starting from the optimized geometries on the B3LYP/def2-TZVP level of theory.

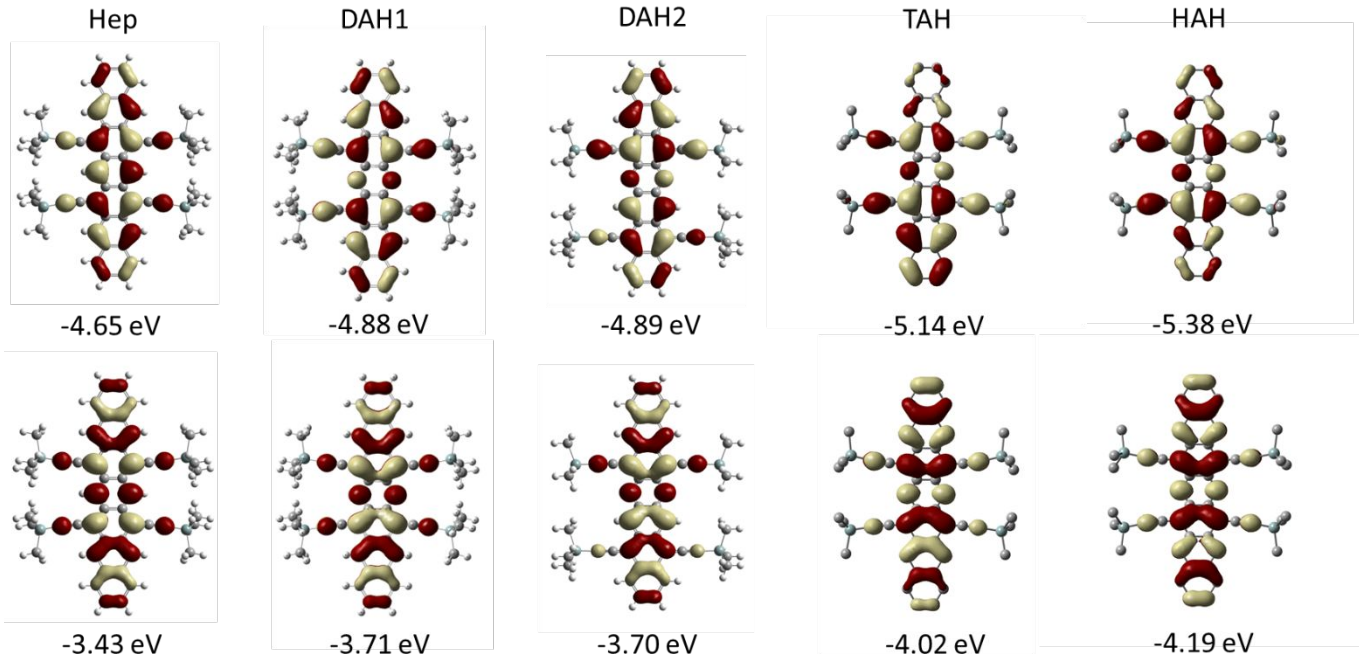

Figure S1. FMO distribution of **Hep**, **DAH1, DAH2, TAH** and **HAH**.

Strain calculation in S-shaped heptacene:

To estimate the strain introduced by the S-shape, heptacene was optimized at the B3LYP/def2-SVP level of theory. From the two obtained crystal structures of **Hep** the heptacene backbone was extracted (TIPS-ethynyl groups were removed) and the empty bonding sites were saturated with hydrogens. Geometry optimization was performed only on the hydrogen atoms the preserve the S-shape and the resulting energy difference between those optimizations and the free optimization of heptacene gives an estimate of the strain energy introduced by the S-shape (20 kJ/mol).

5. IR Spectroscopy

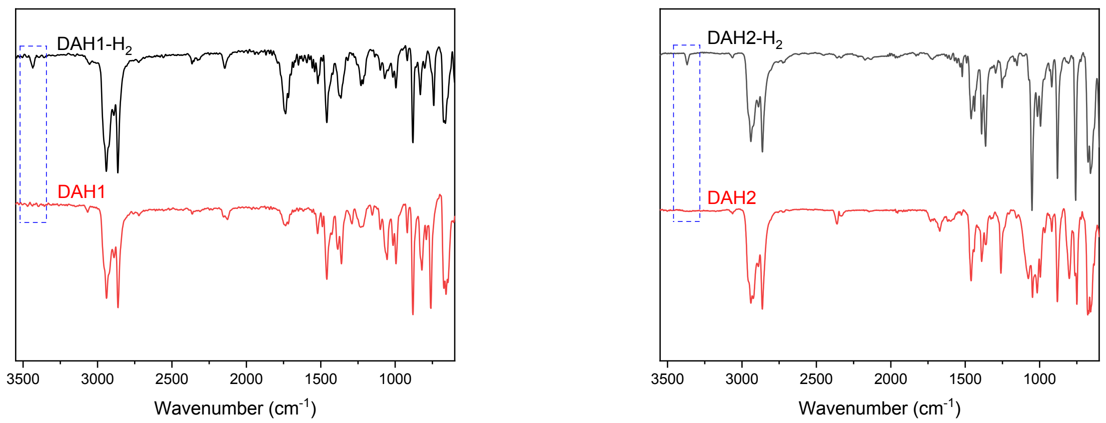

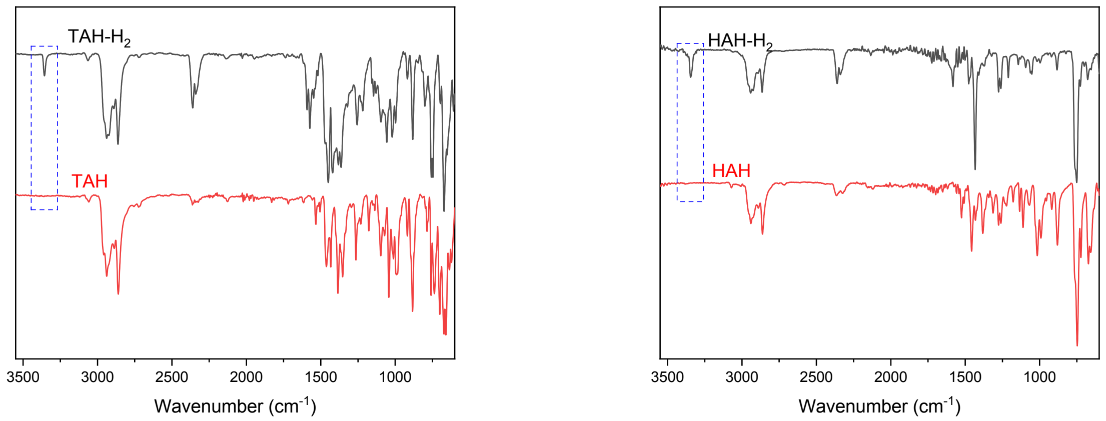

Figure S2. Comparison of IR spectra of dihydro compounds (black) and azaheptacenes (red). The lack of the N-H vibration mode around 3350 cm^{-1} for **DAH1**, **DAH2**, **TAH** and **HAH** indicates the existence of the oxidized azaheptacenes.

6. Mass Spectra

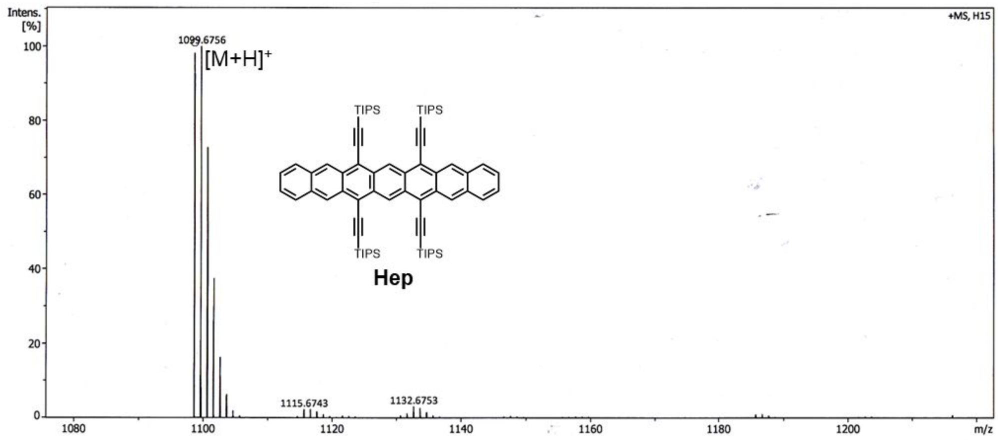

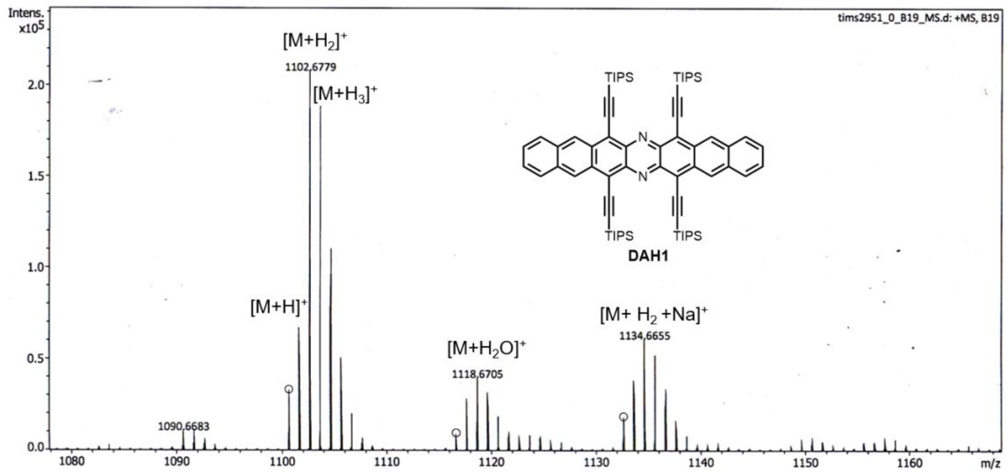

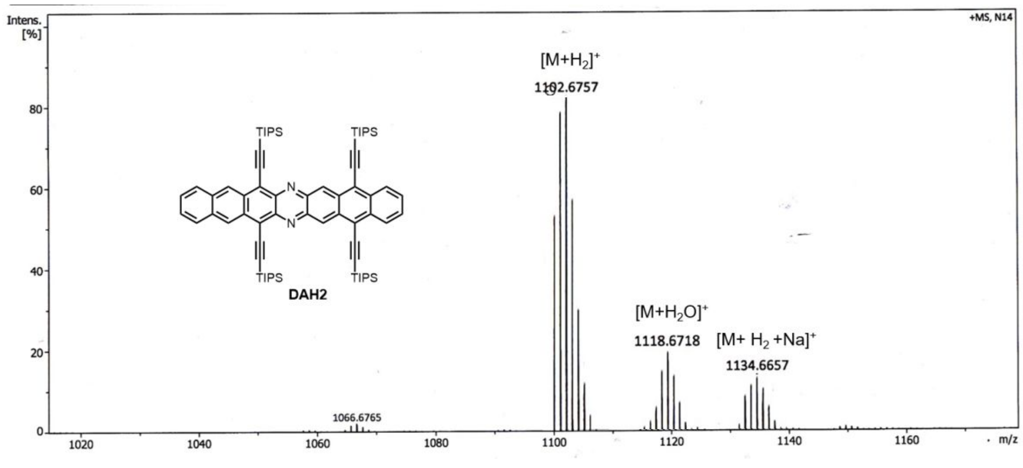

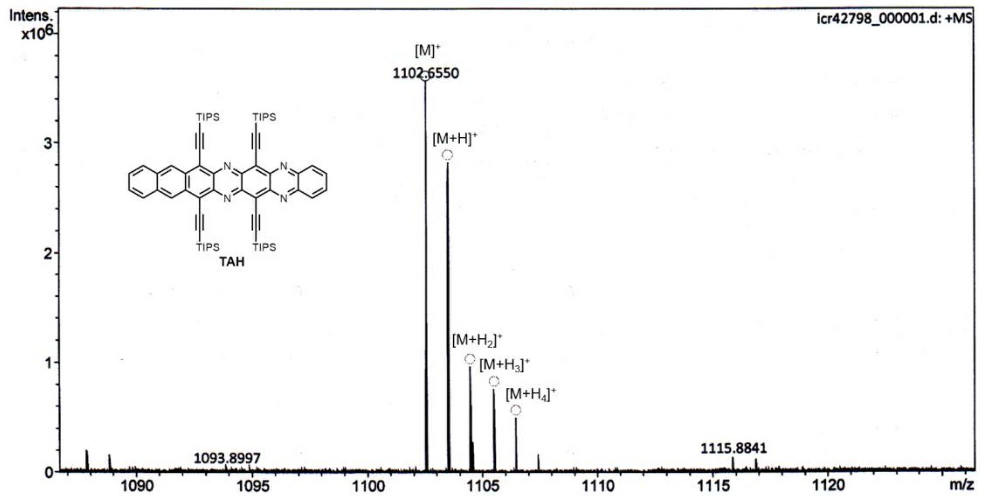

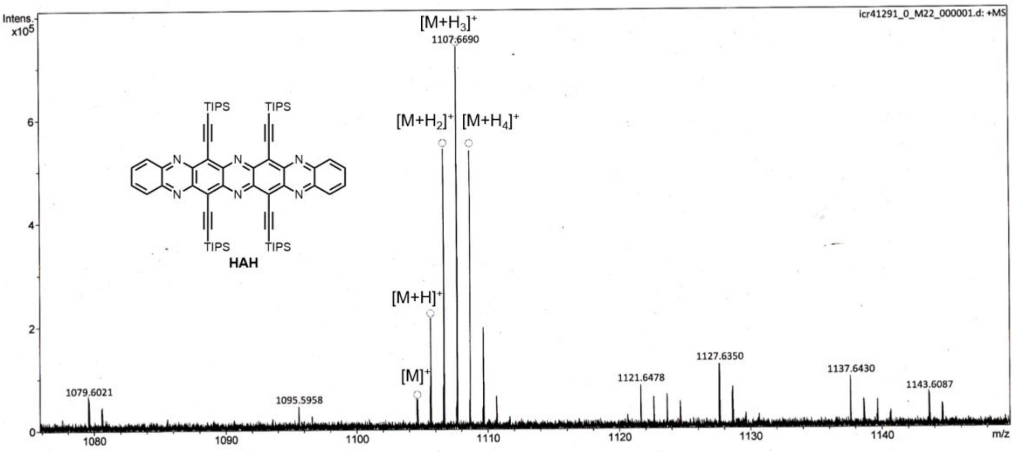

Figure S3: Mass spectra (MALDI-pos.) of the (aza)heptacenes.

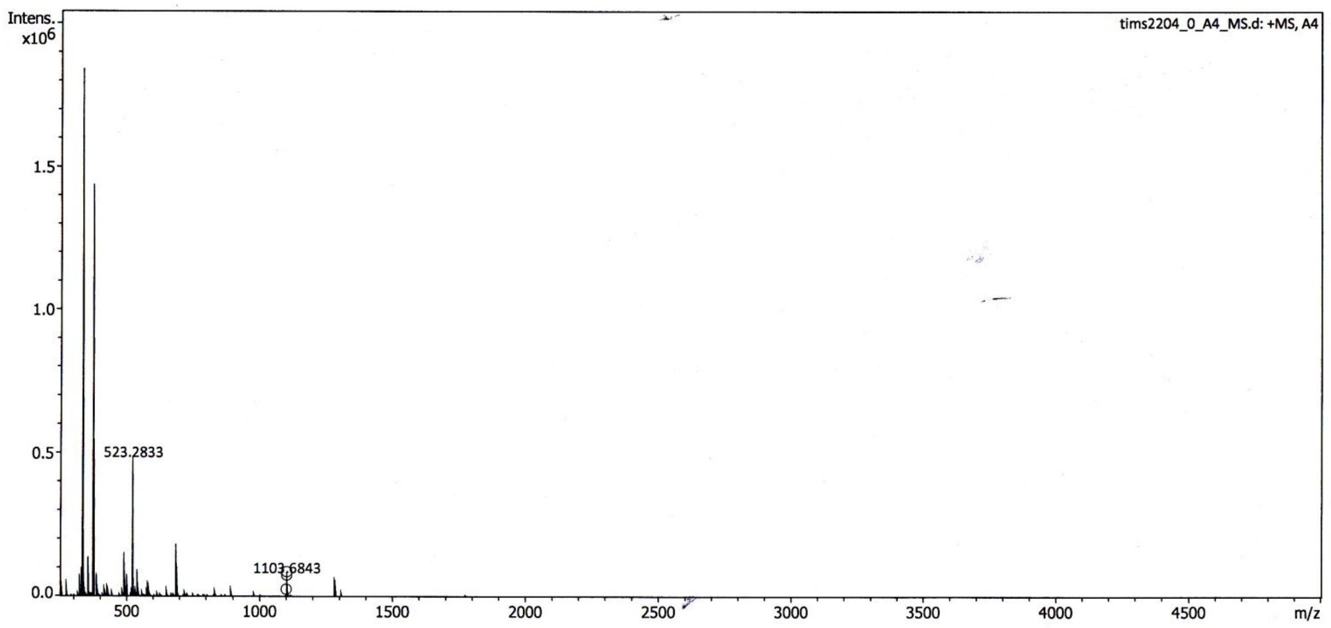

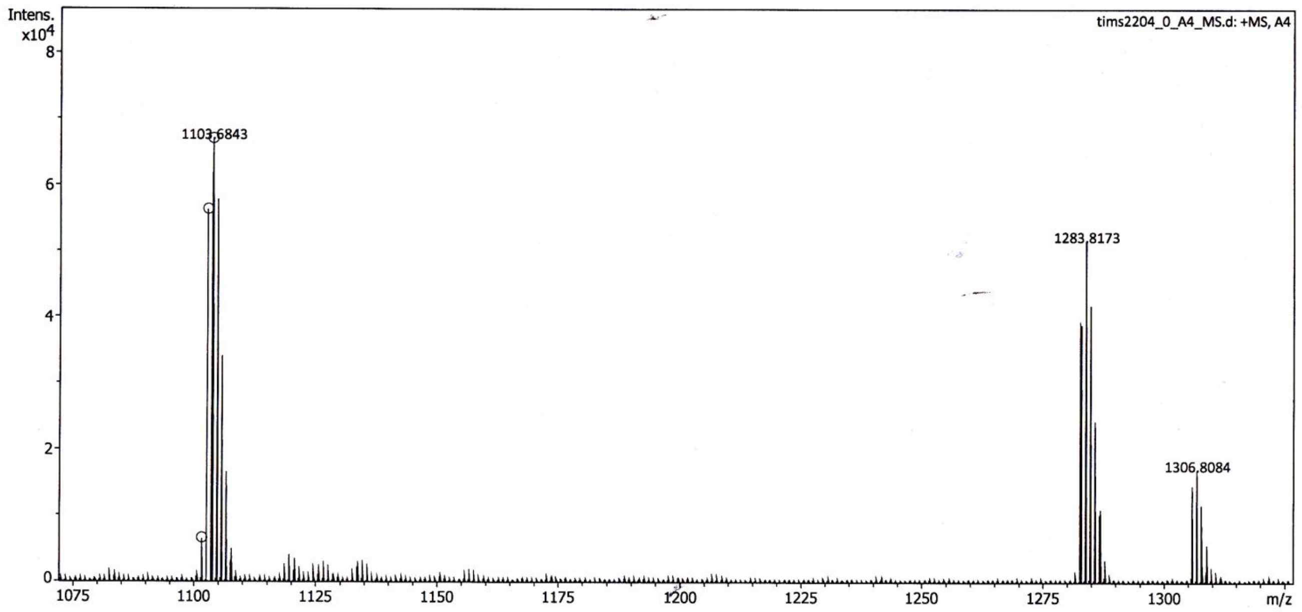

Figure S4. Mass spectra (MALDI-pos.) of **DAH1** after storing on benchtop for 8 months. Mass spectrum shows the formation of its dihydro-species and other by-products.

7. Cyclic Voltammetry

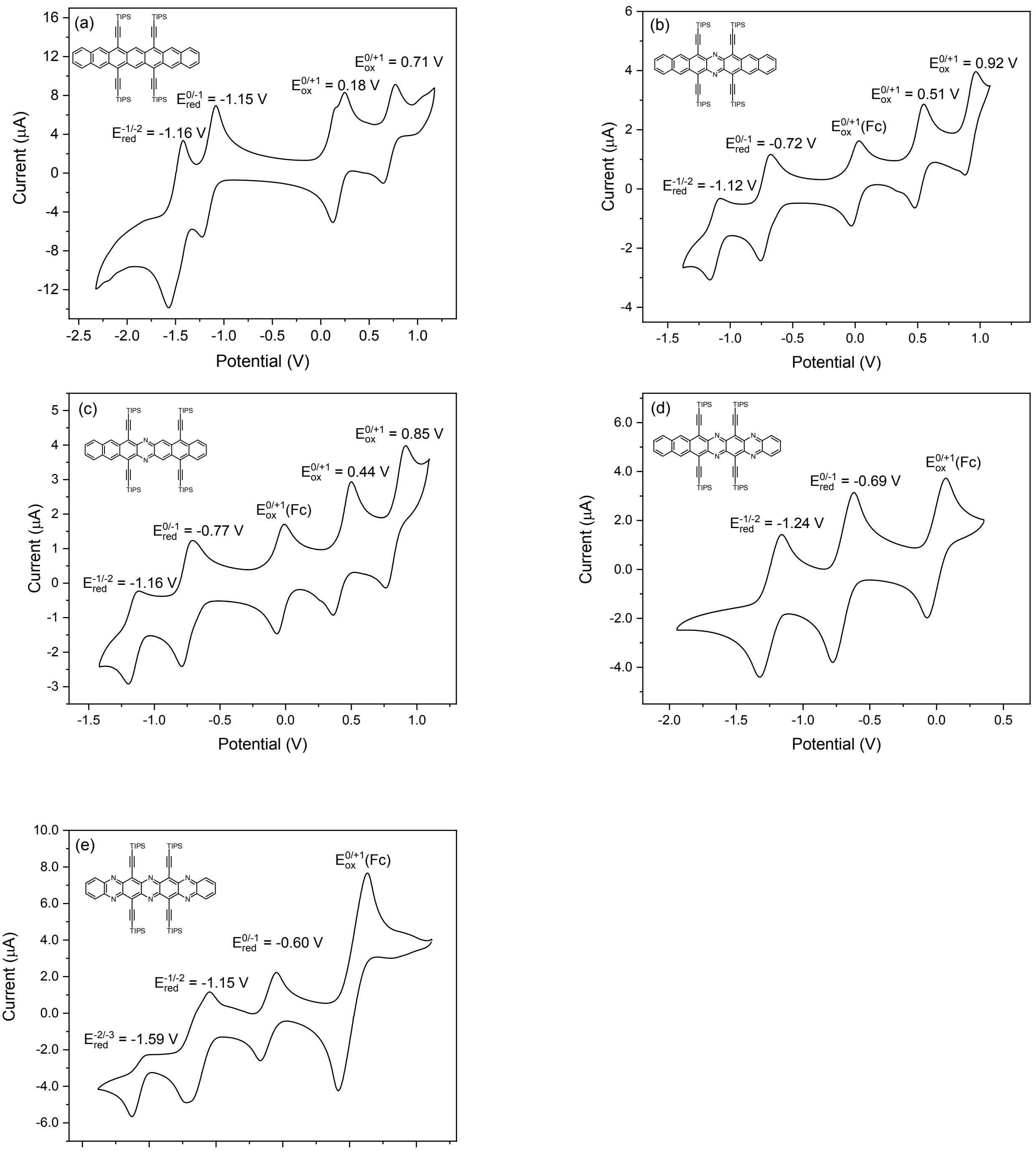

Figure S5. Cyclic voltammograms of (a) **Hep**, (b) **DAH1**, (c) **DAH2**, (d) **TAH** and (e) **HAH** in DCM (Fc/Fc^+ as reference, Pt as working electrode and Bu_4NPF_6 as electrolyte, scanning speed 100 mV/s).

8. Time-dependent NMR Spectra

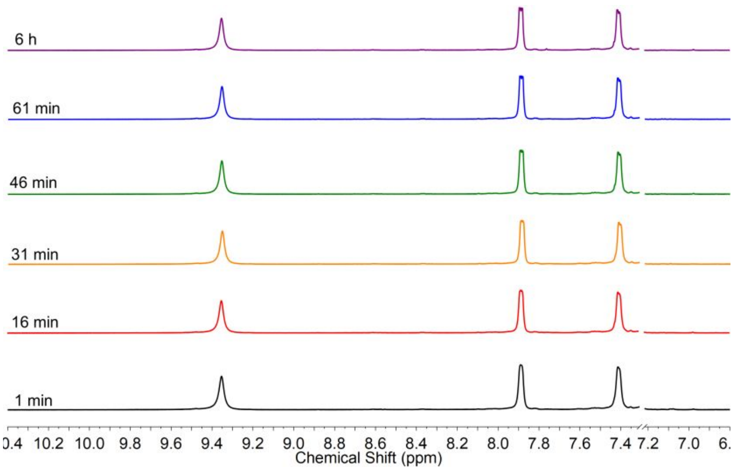

Figure S6. Time-dependent ^{1}H NMR spectra of **DAH1** in $CDCl_3$ under ambient conditions magnifying all signals in the aromatic region. Chloroform peak was omitted for clarity.

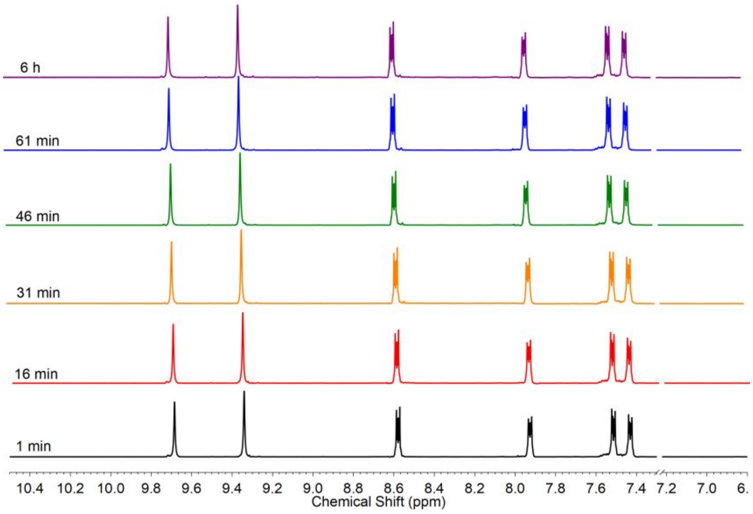

Figure S7. Time-dependent ^{1}H NMR spectra of **DAH2** in $CDCl_3$ under ambient conditions magnifying all signals in the aromatic region. Chloroform peak was omitted for clarity.

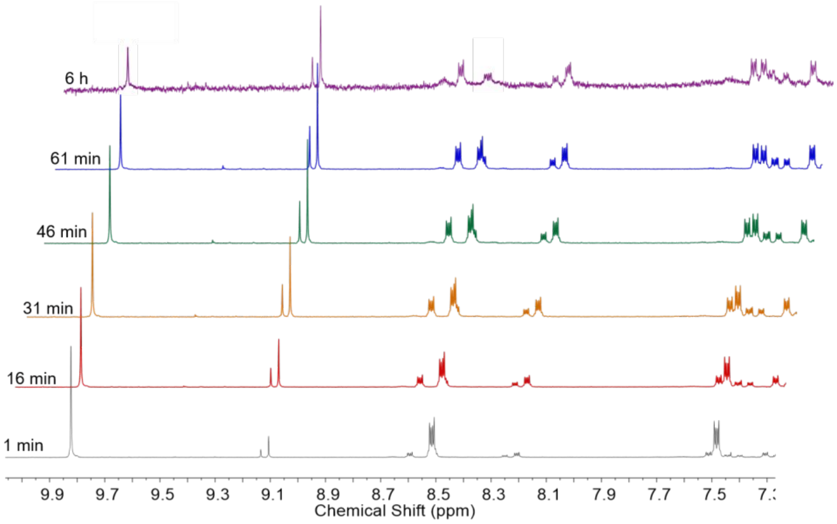

Figure S8. Time-dependent ^{1}H NMR spectra of **DAH3** in $CDCl_3$ under ambient conditions magnifying all signals in the aromatic region.

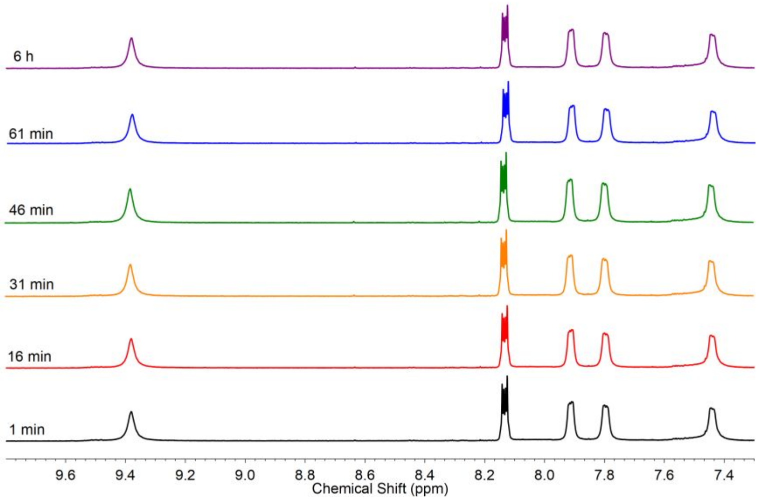

Figure S9. Time-dependent ^{1}H NMR spectra of **TAH** in $CDCl_3$ under ambient conditions magnifying all signals in the aromatic region.

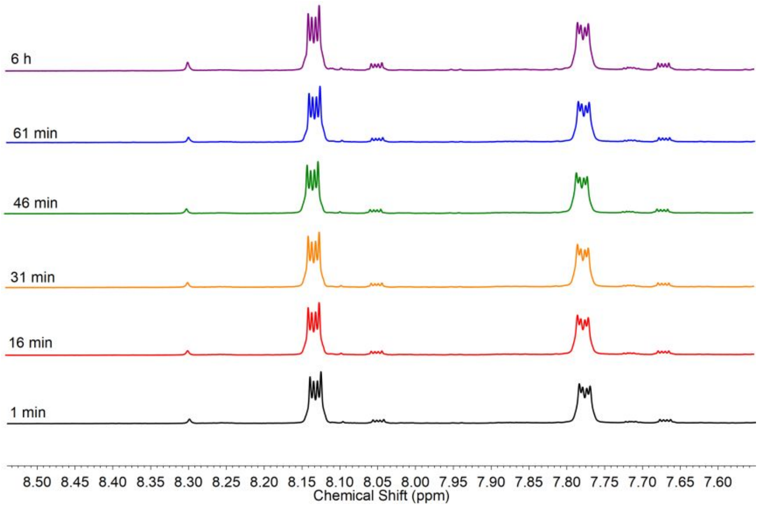

Figure S10. Time-dependent ^{1}H NMR spectra of **HAH** in $CDCl_3$ under ambient conditions magnifying all signals in the aromatic region. Weak signals originating from degradation specie (**HAH-H_2**) was observed because of the spontaneous back reduction process of **HAH**, and this part occupied about 7% (estimated from the integration).

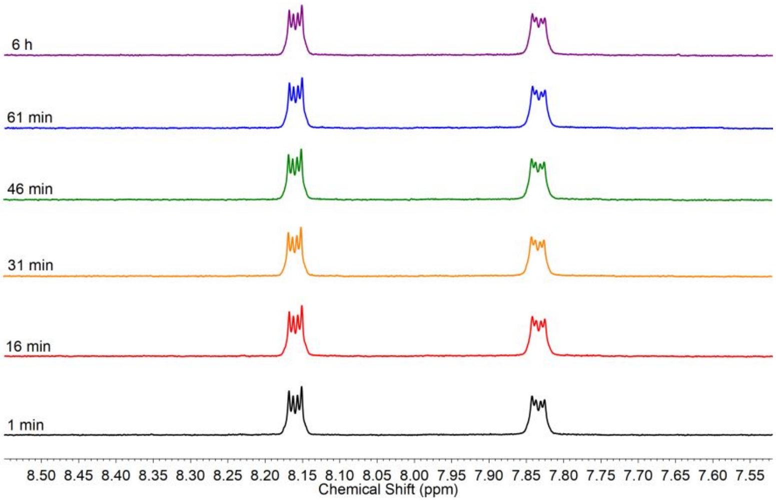

Figure S11. Time-dependent ^{1}H NMR spectra of **HAH** in CD_2Cl_2 with PbO_2 under an inert atmosphere magnifying all signals in the aromatic region.

9. UV–Vis Absorption Spectra

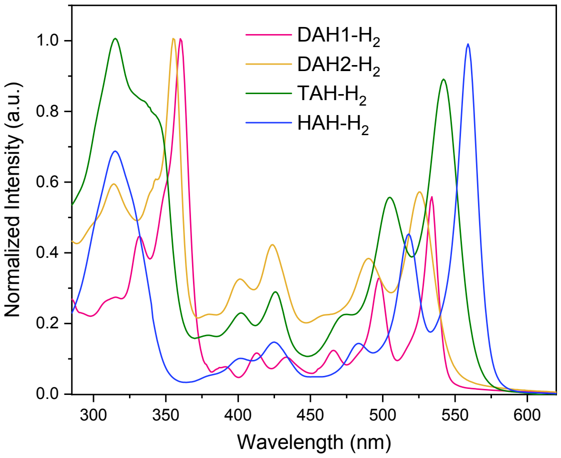

Figure S12. Normalized UV-Vis absorption spectra of *N,N'*-dihydro compounds in DCM (10^{-5} mol L^{-1}).

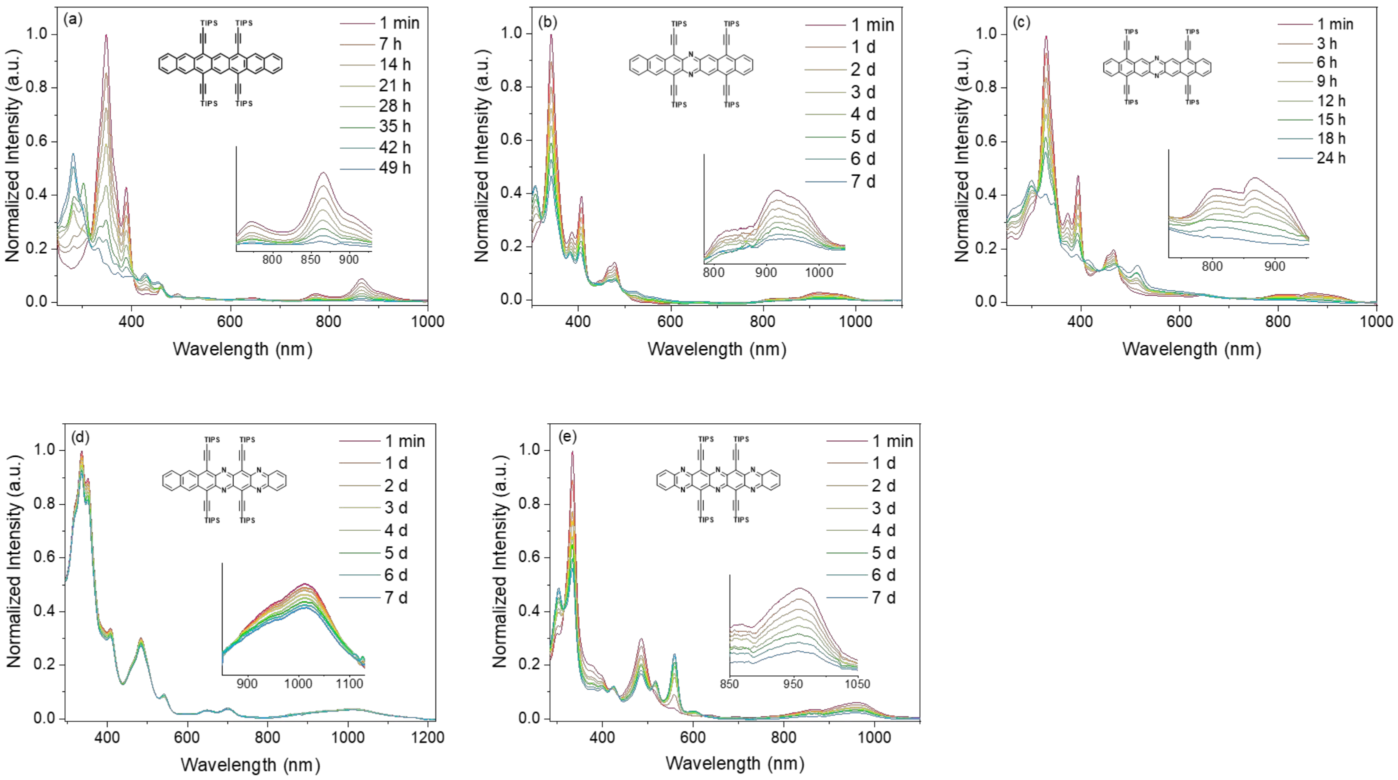

Figure S13. Time-dependent evolution of UV-Vis spectra of (a) **Hep**, (b) **DAH2**, (c) **DAH3**, (d) **TAH** and (e) **HAH** in dilute solutions on the benchtop (10^{-5} mol L^{-1} in dry DCM) under ambient conditions at room temperature. The inset shows magnifications of the time-dependent evolution of the p-bands

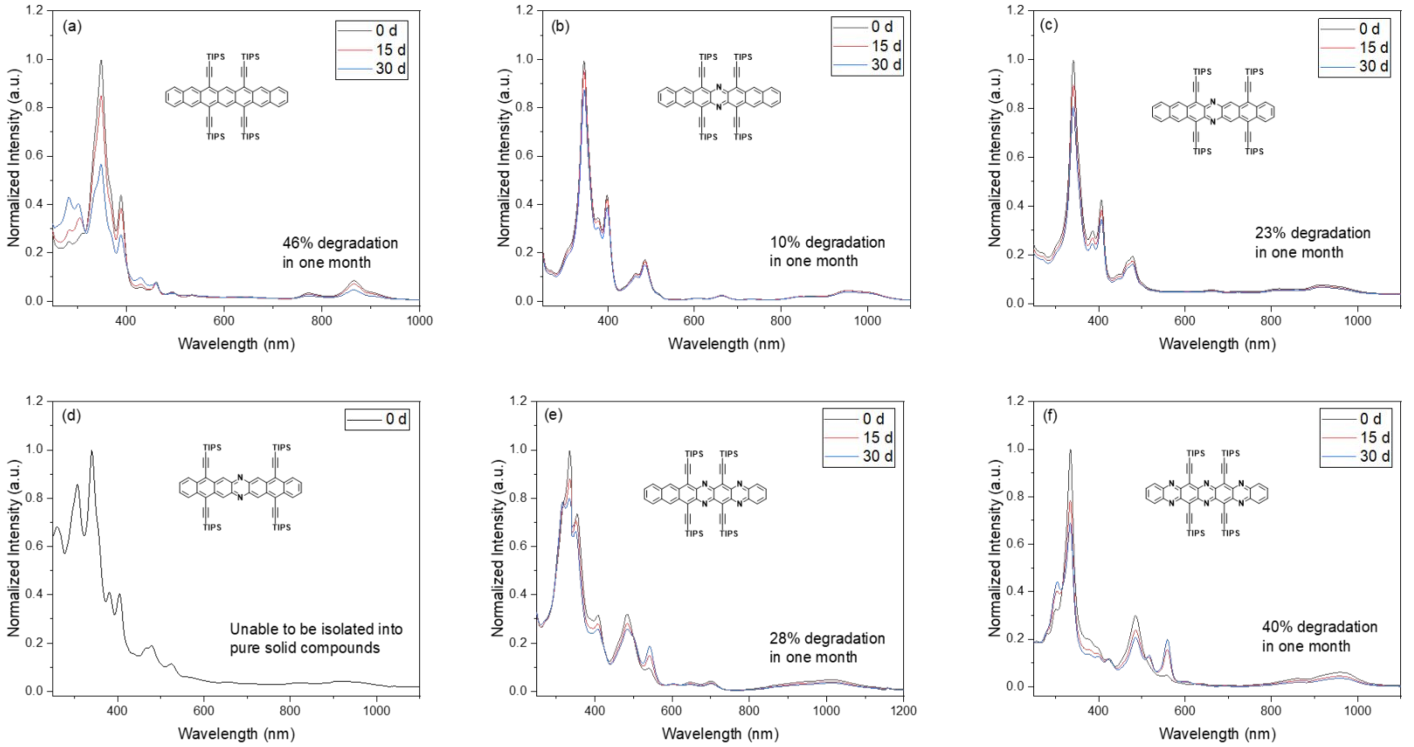

Figure S14. Time-dependent evolution of UV-Vis spectra of (a) **Hep**, (b) **DAH1**, (c) **DAH2**, (d) **DAH3**, (e) **TAH** and (f) **HAH**. The degradation content is estimated from the absorption maxima intensity (100% – $I(\lambda_{abs,\ max})/I_0(\lambda_{abs,\ max})$). The compounds were stored as solids on the benchtop under ambient conditions at room temperature, and then the solution samples for UV-Vis measurement were prepared in DCM solution (10^{-5} mol L^{-1}).

10. Photos of the As-prepared Compounds

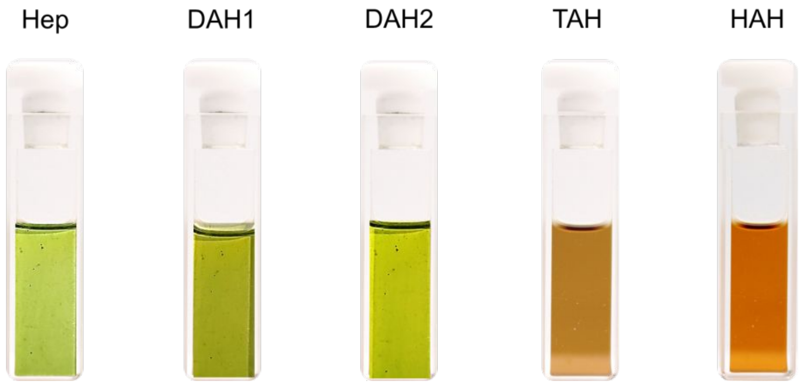

Figure S15. Photos of the respective dilute solutions (10^{-5} mol L^{-1}) of (aza)heptacenes.

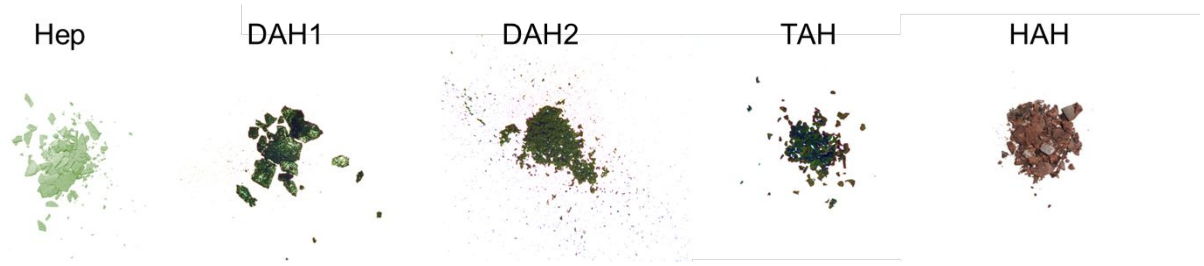

Figure S16. Photos of the respective solids of (aza)heptacenes.

11. UPLC Analysis

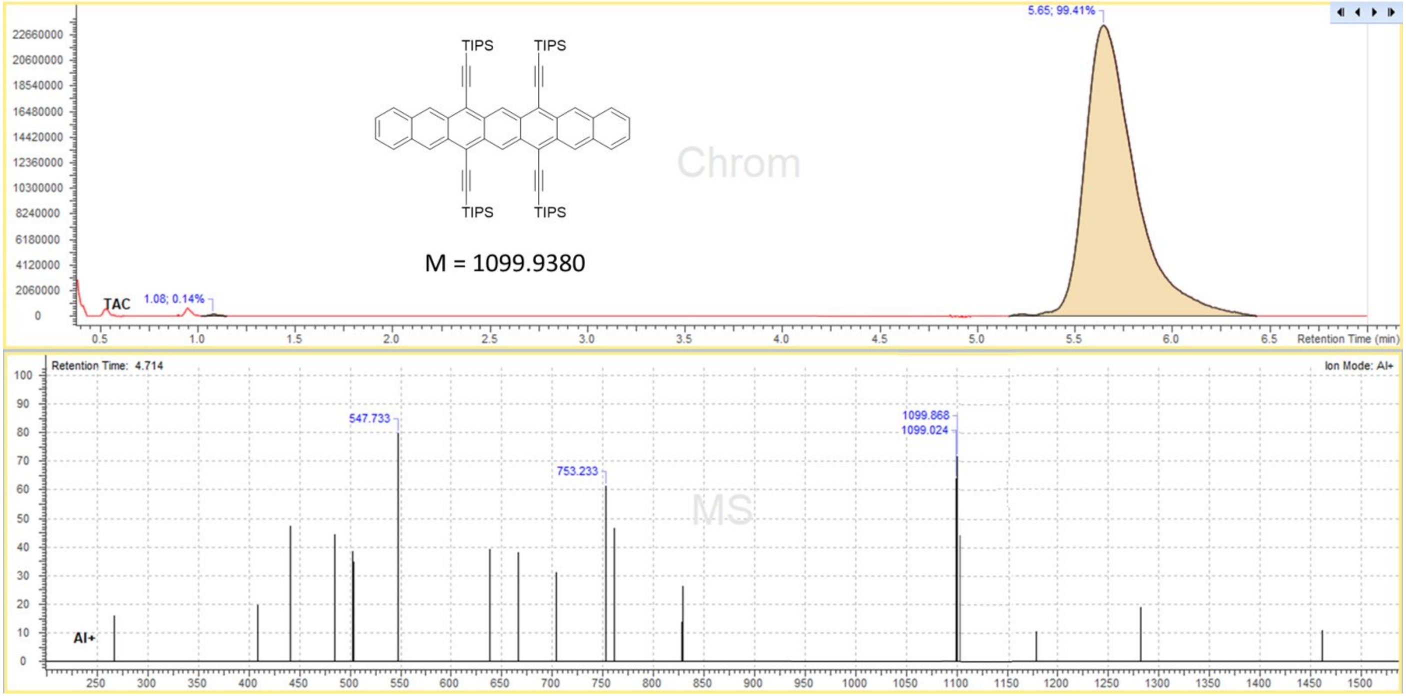

Figure S17. UPLC spectrum of **Hep**.

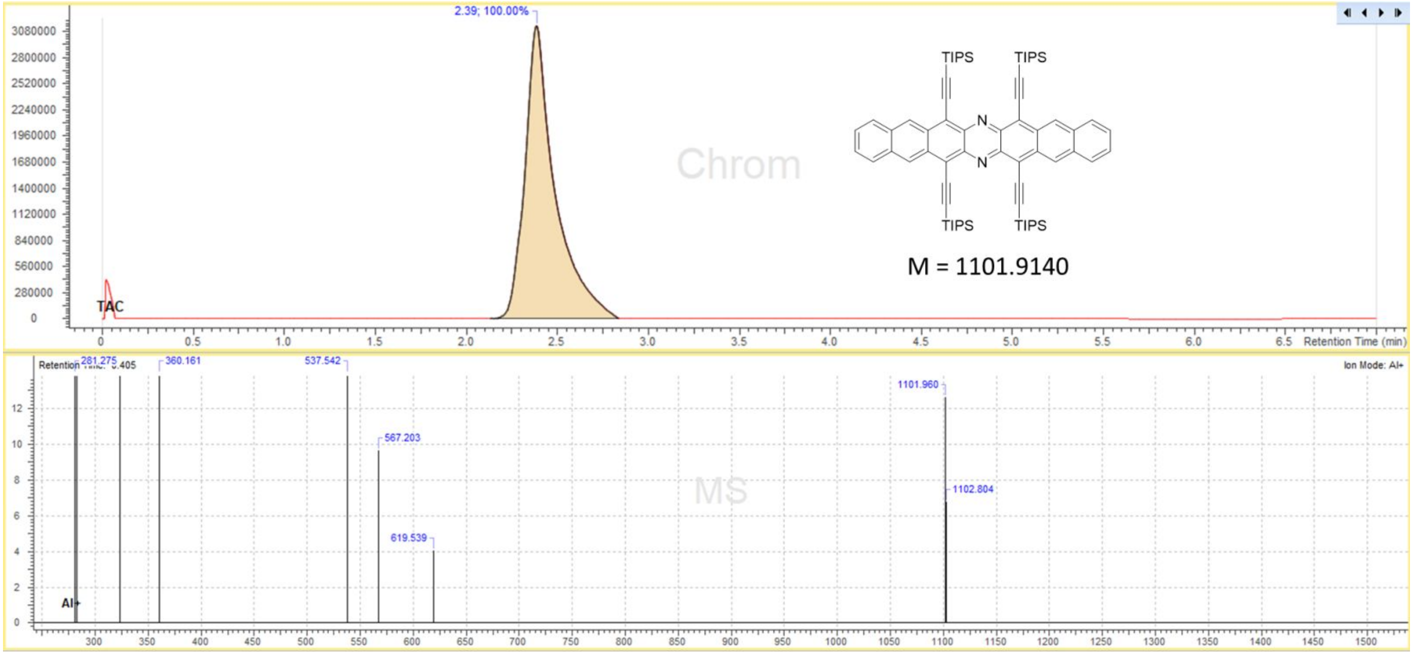

Figure S18. UPLC spectrum of **DAH1**.

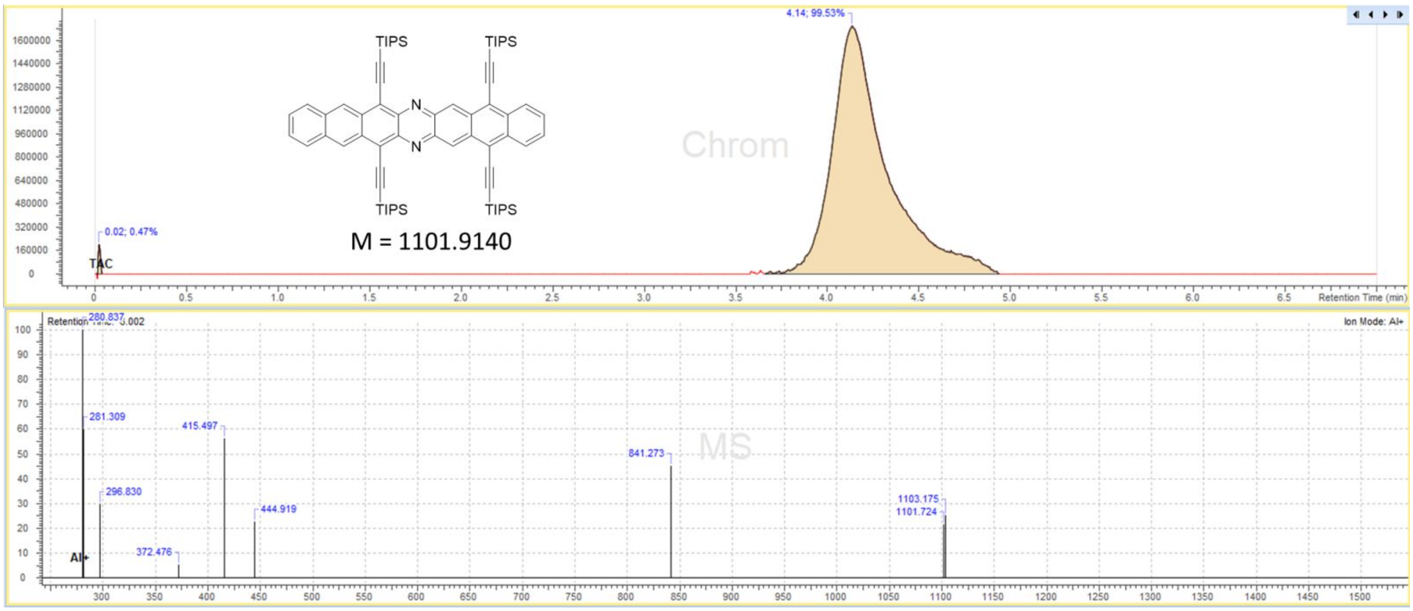

Figure S19. UPLC spectrum of **DAH2**.

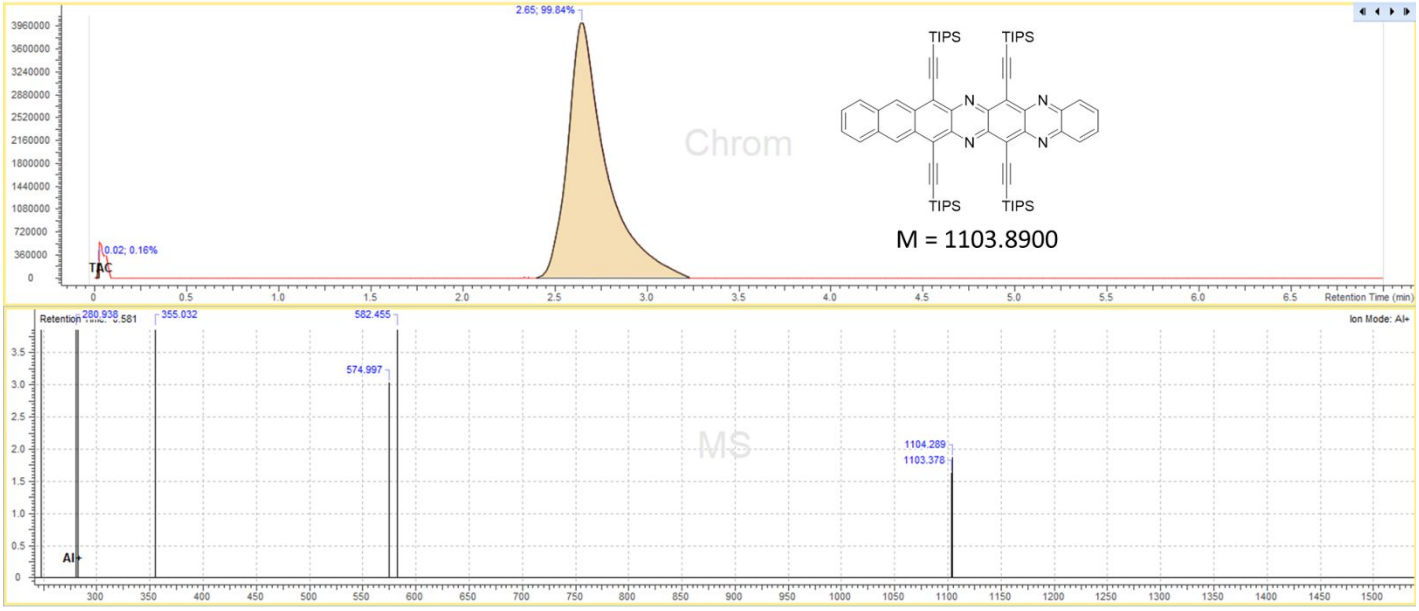

Figure S20. UPLC spectrum of **TAH**.

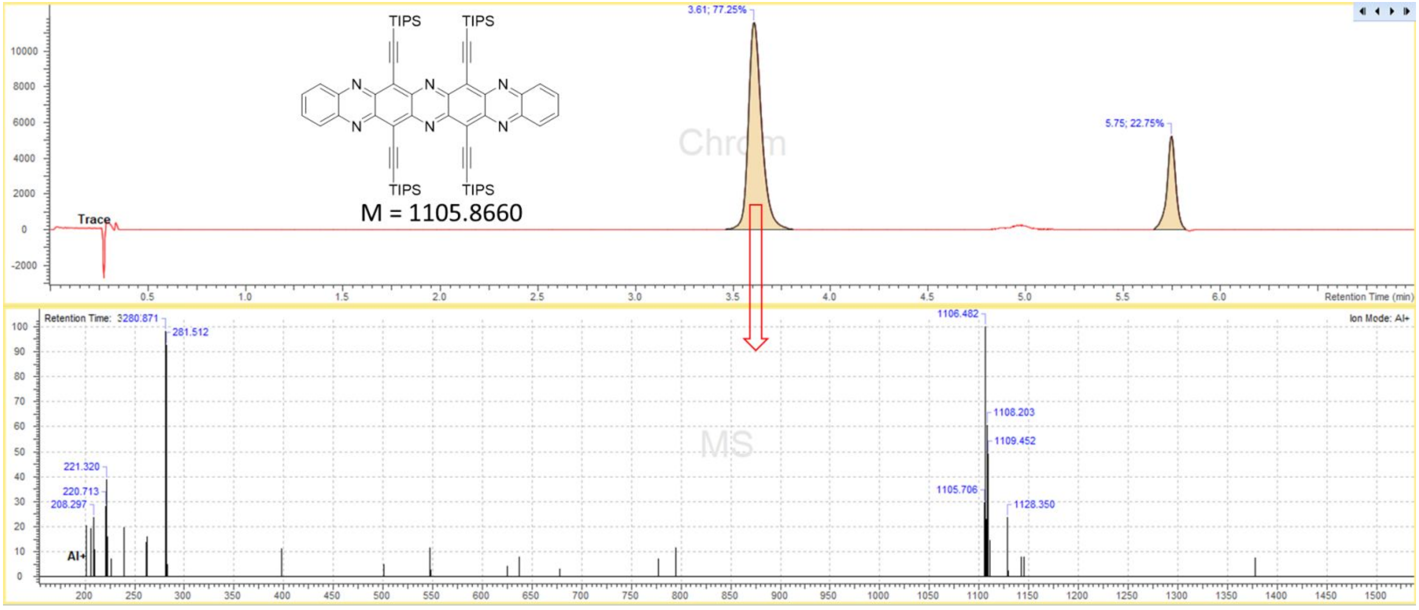

Figure S21. UPLC spectrum of **HAH**.

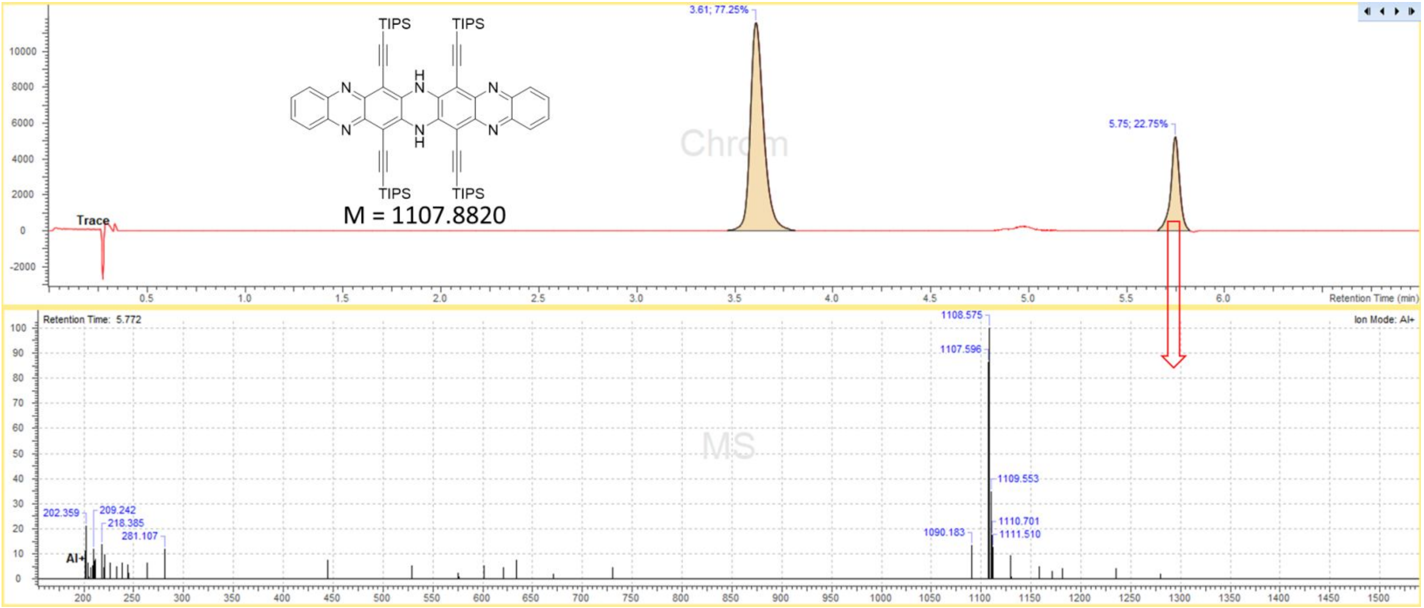

Figure S22. UPL chromatography of **HAH** also indicates the formation of its dihydro-species **HAH-H_2**.

12. Crystal Structures

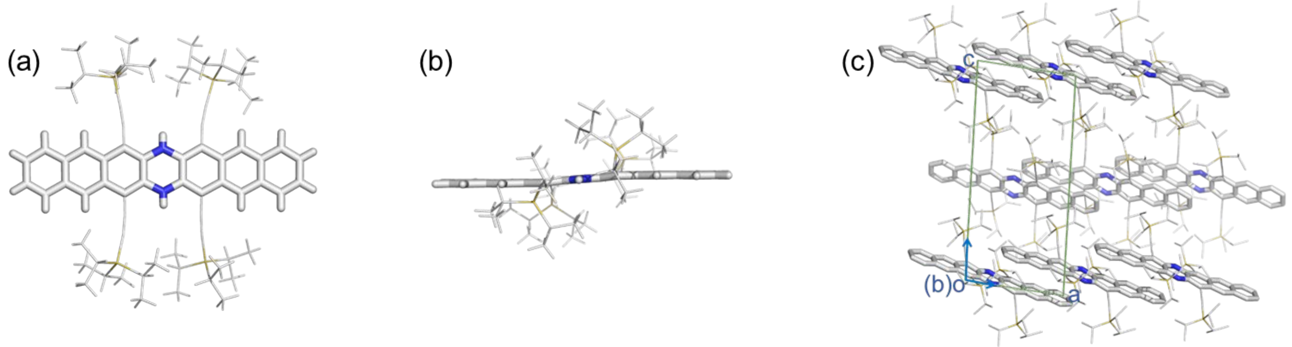

Figure S23. Solid-state packing obtained for **DAH1-H$_2$**. (a) Top view, (b) side view and (c) packing motif. **DAH1-H$_2$** was found as the only structure in the sample, without any other possible precursors for by-product **S1**.

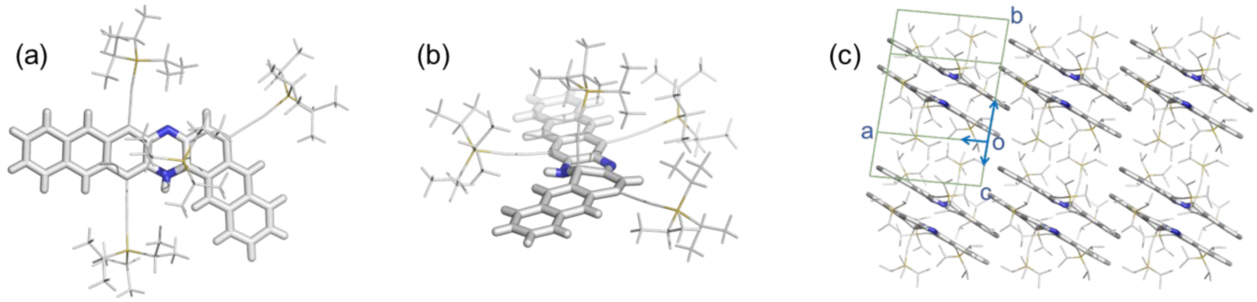

Figure S24. Solid-state packing obtained for by-product **S1**. (a) Top view, (b) side view and (b) packing motif.

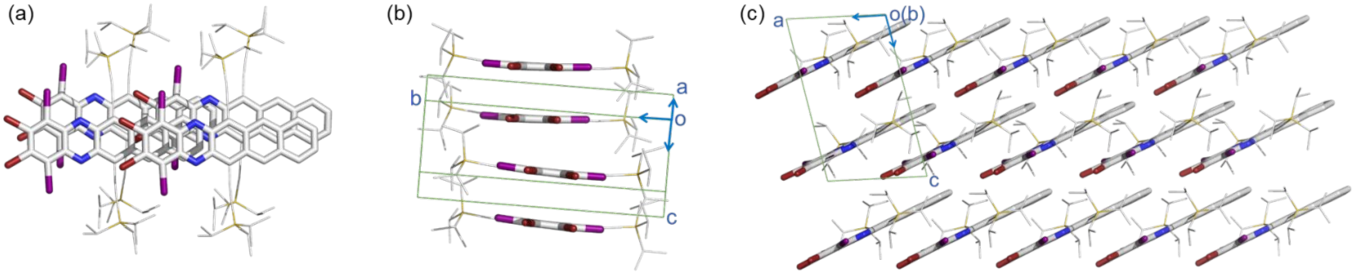

Figure S25. Solid-state packing obtained for **5a**. (a) Top view, (b) side view and (c) packing motif.

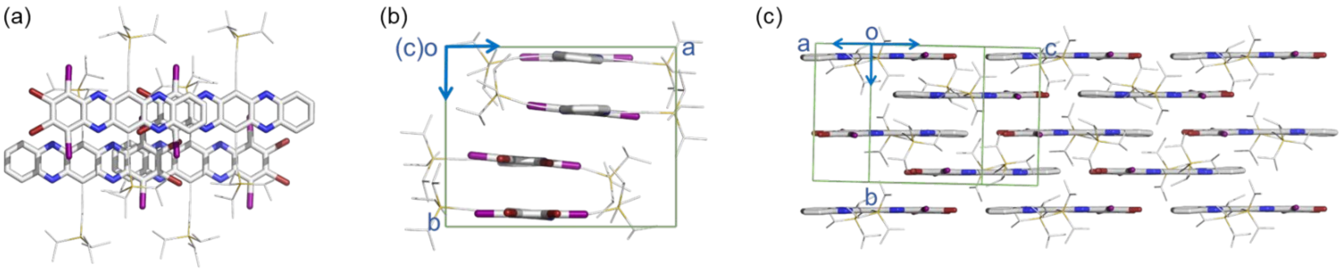

Figure S26. Solid-state packing obtained for **5b**. (a) Top view, (b) side view and (c) packing motif.

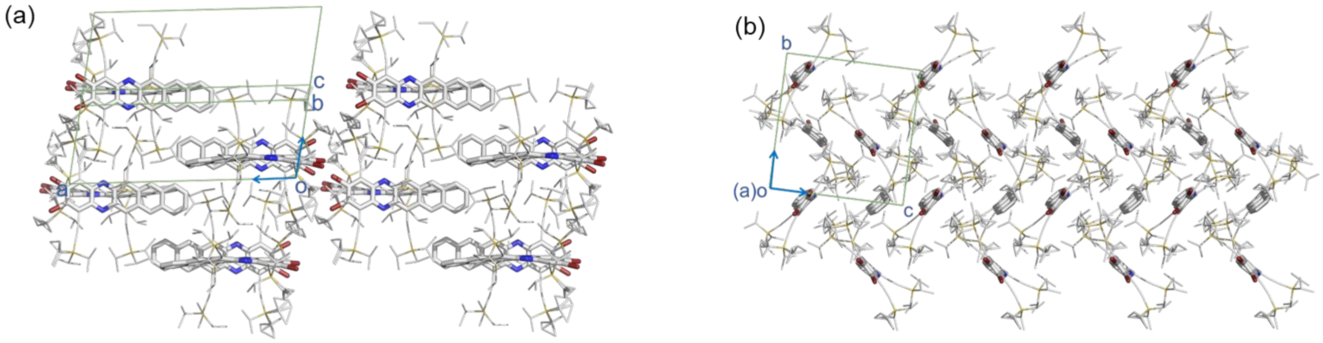

Figure S27. Solid-state packing obtained for **6a**. (a) Top view and (b) side view.

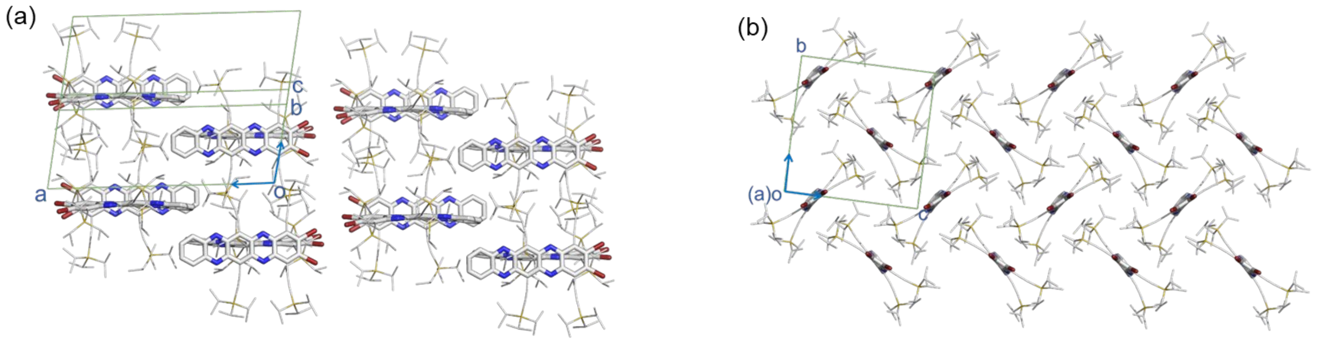

Figure S28. Solid-state packing obtained for **6b**. (a) Top view and (b) side view.

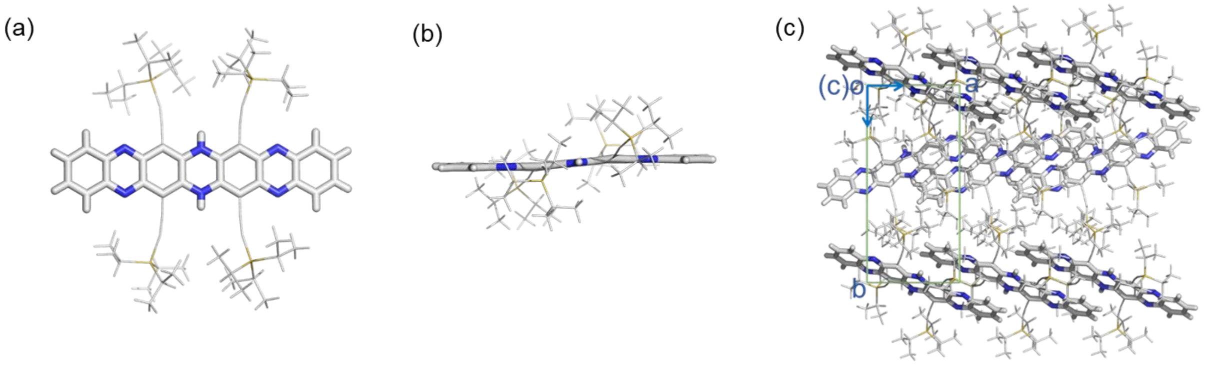

Figure S29. Solid-state packing obtained for **HAH**. (a) Top view, (b) side view and (c) packing motif. Protons were found at the central N atom in the crystalline, and this position seems to be occupied about 50%.

13. NMR Spectra

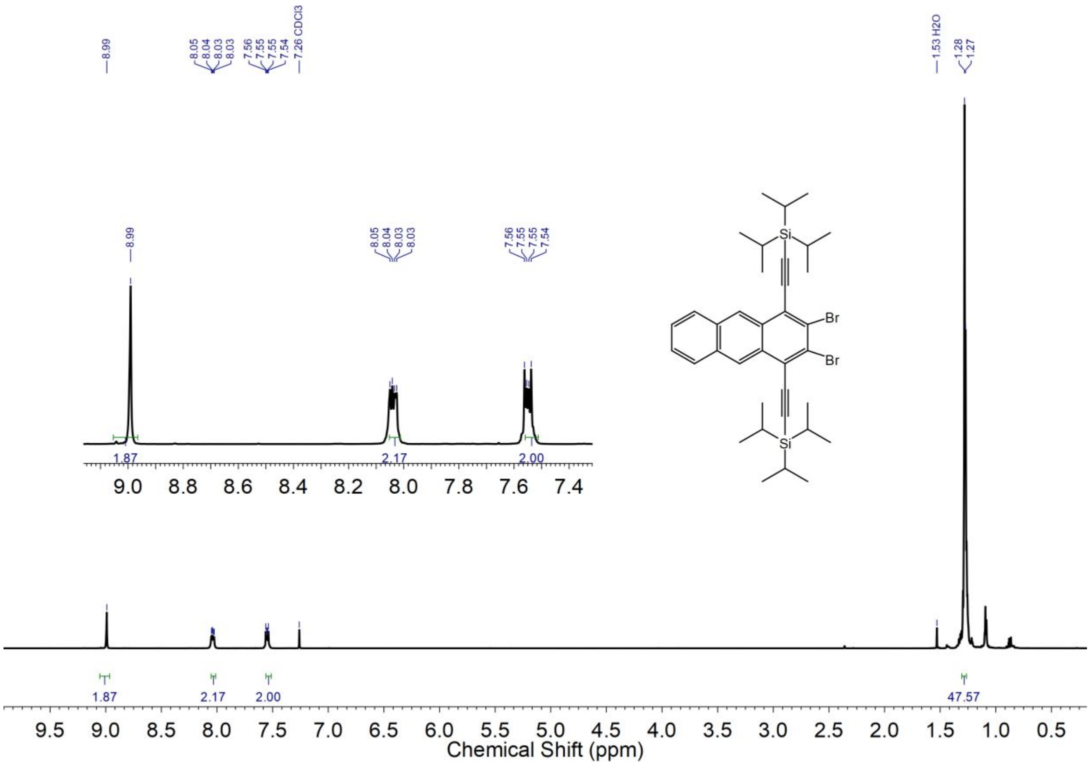

Figure S30. ^{1}H NMR spectrum (500 MHz, 295 K) of **3a** in $CDCl_3$.

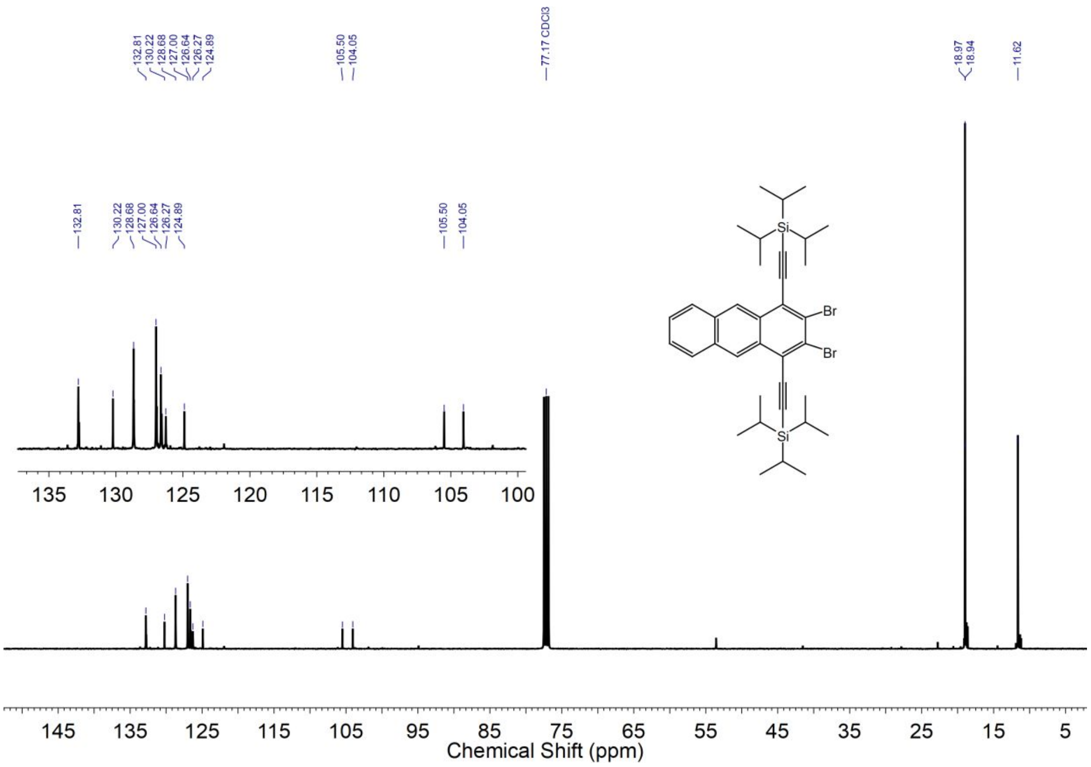

Figure S31. $^{13}C\{^{1}H\}$ NMR spectrum (126 MHz, 295 K) of **3a** in $CDCl_3$.

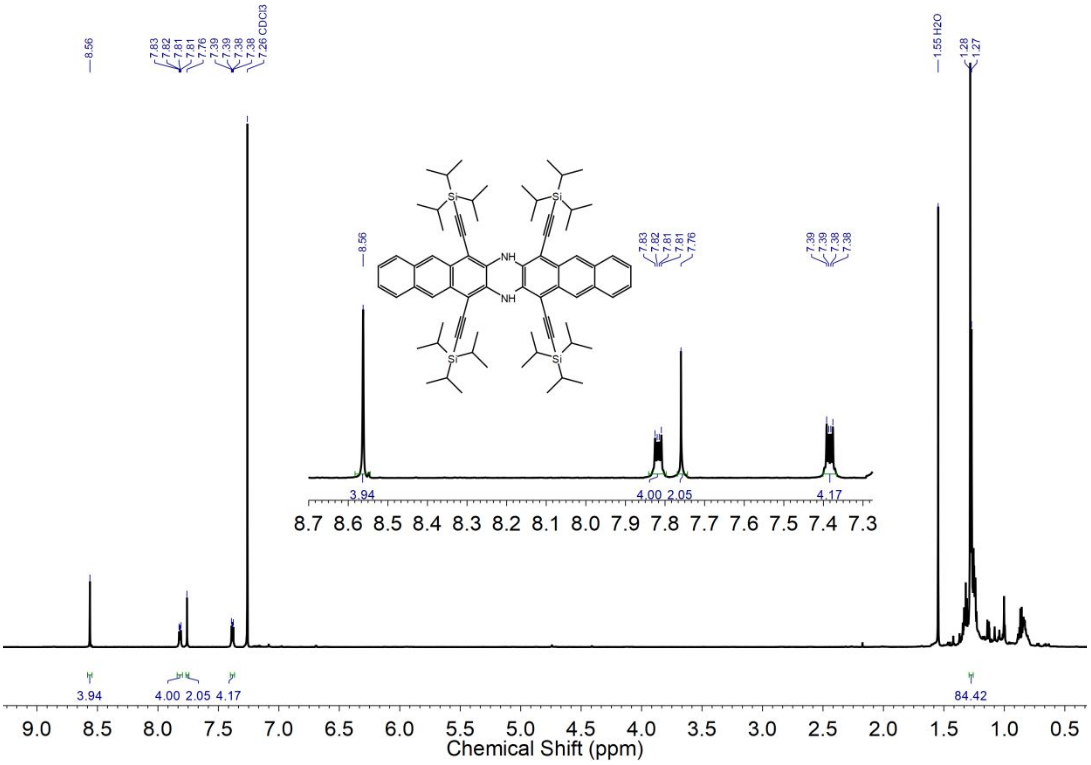

Figure S32. ^{1}H NMR spectrum (600 MHz, 295 K) of **DAH1-H$_2$** in $CDCl_3$.

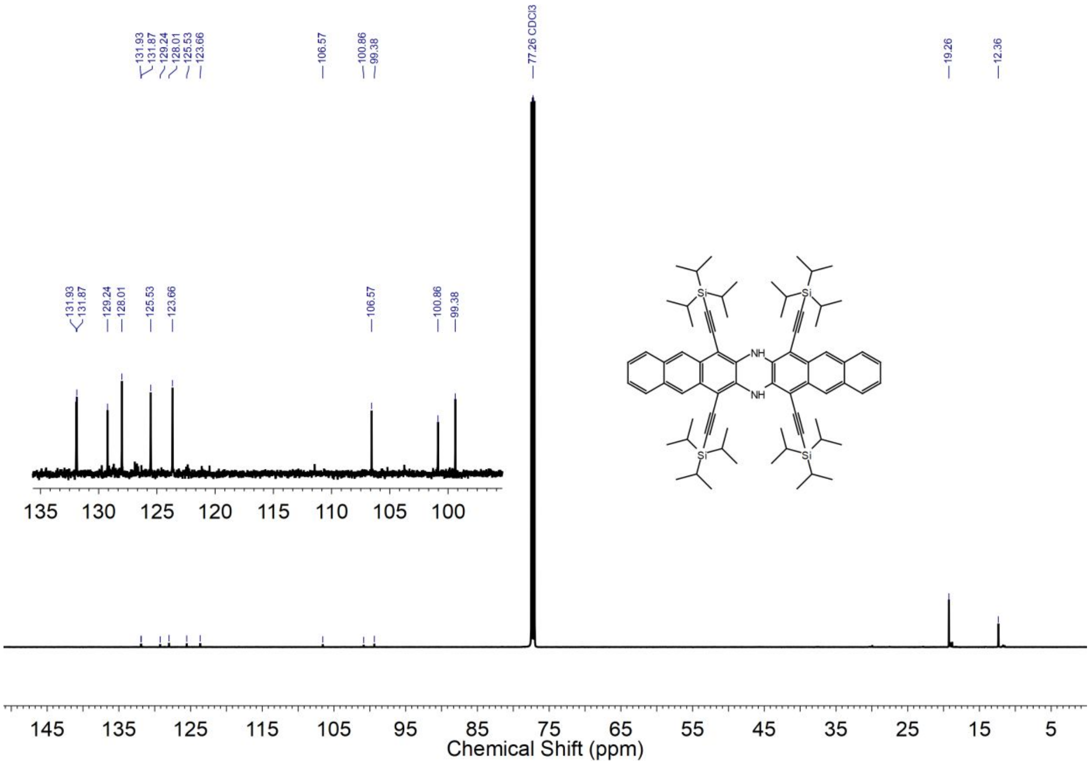

Figure S33. ^{13}C{^{1}H} NMR spectrum (151 MHz, 295 K) of **DAH1-H$_2$** in $CDCl_3$.

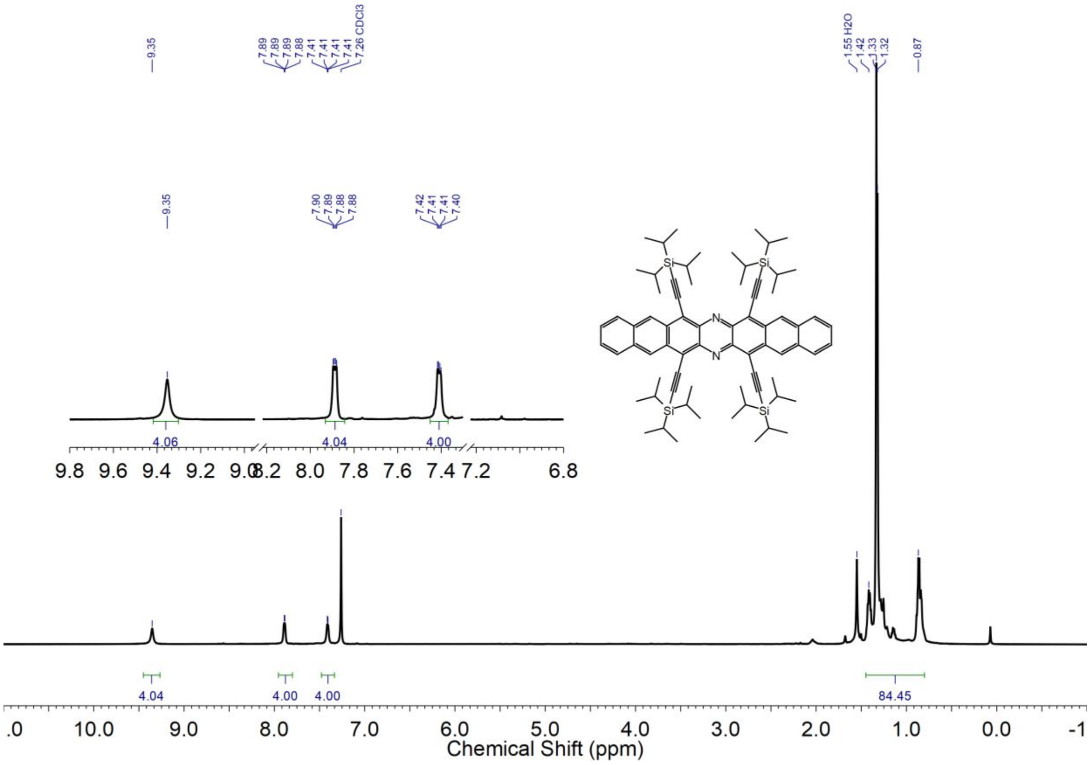

Figure S34. ^{1}H NMR spectrum (600 MHz, 295 K) of **DAH1** in $CDCl_3$.

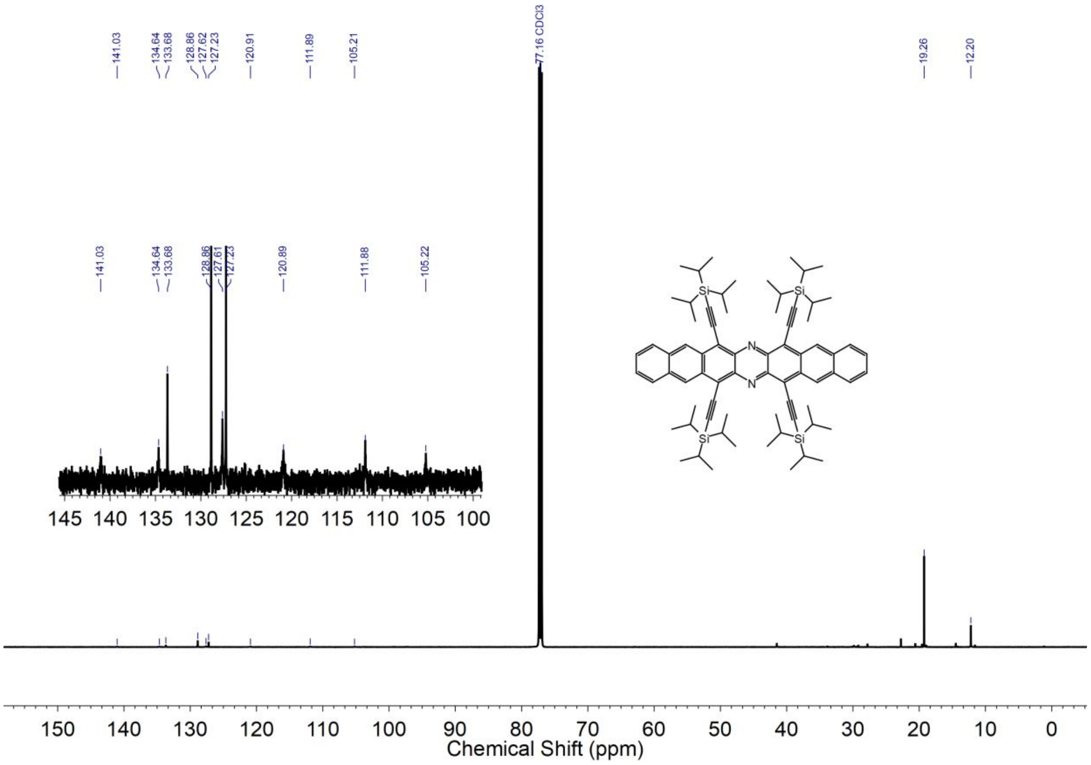

Figure S35. $^{13}C\{^{1}H\}$ NMR spectrum (151 MHz, 295 K) of **DAH1** in $CDCl_3$.

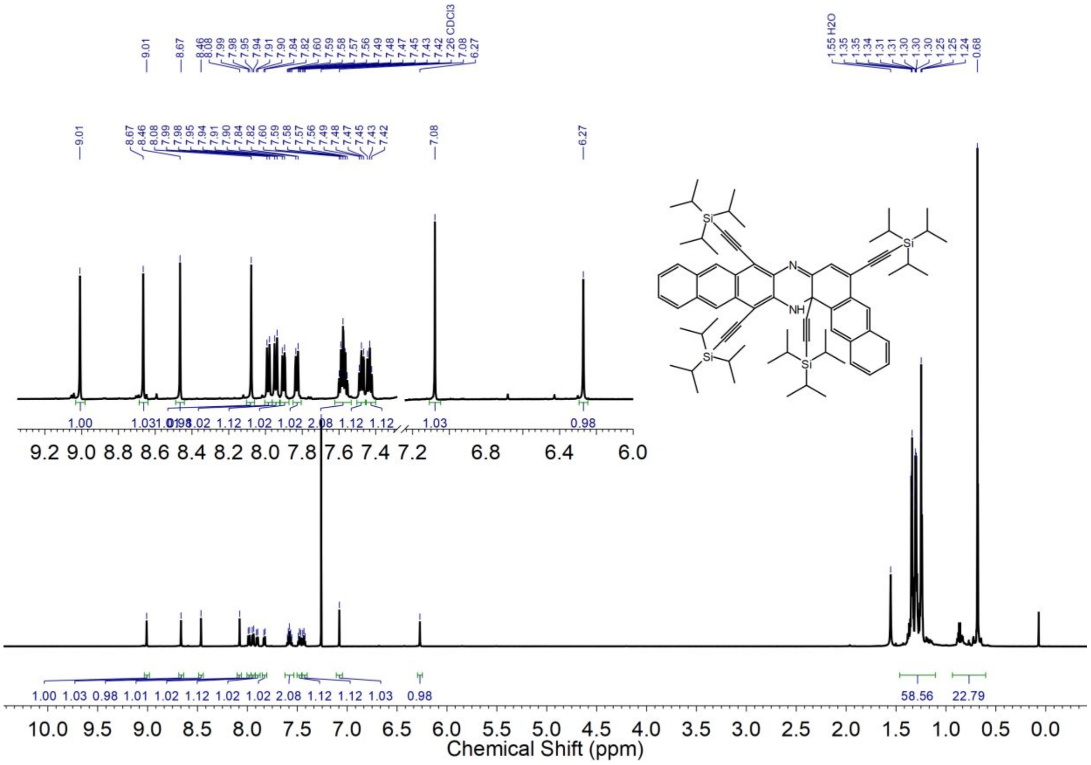

Figure S36. ^{1}H NMR spectrum (600 MHz, 295 K) of **S1** in $CDCl_3$.

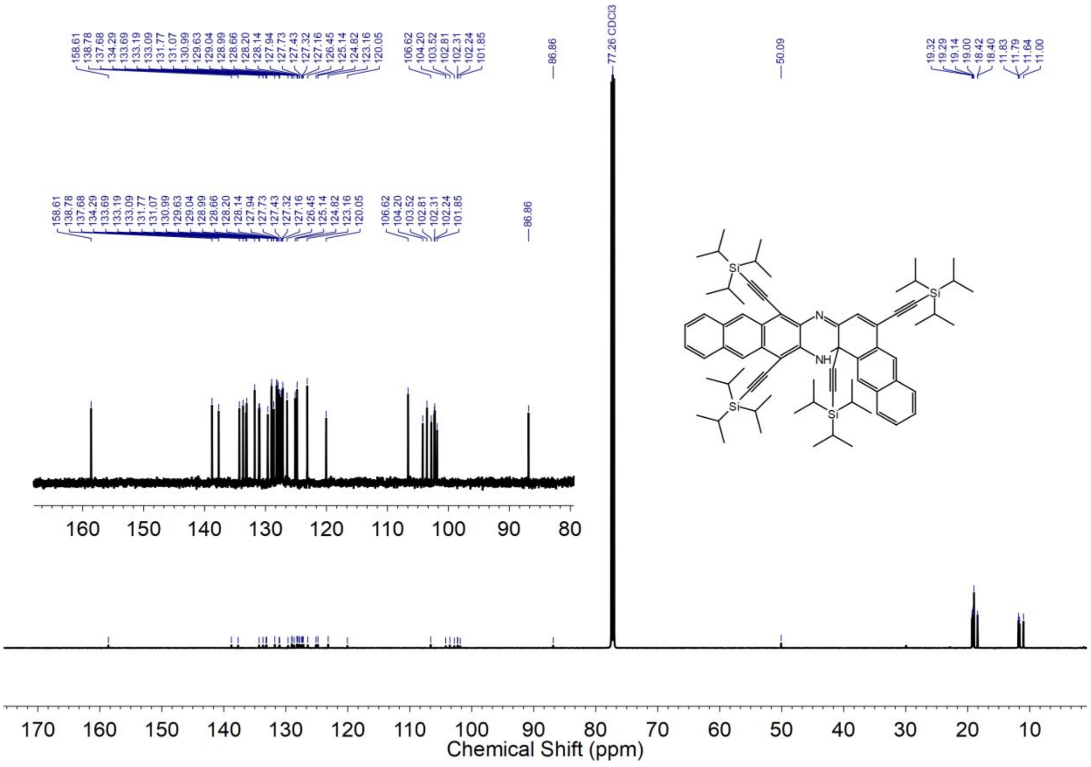

Figure S37. $^{13}C\{^{1}H\}$ NMR spectrum (151 MHz, 295 K) of **S1** in $CDCl_3$.

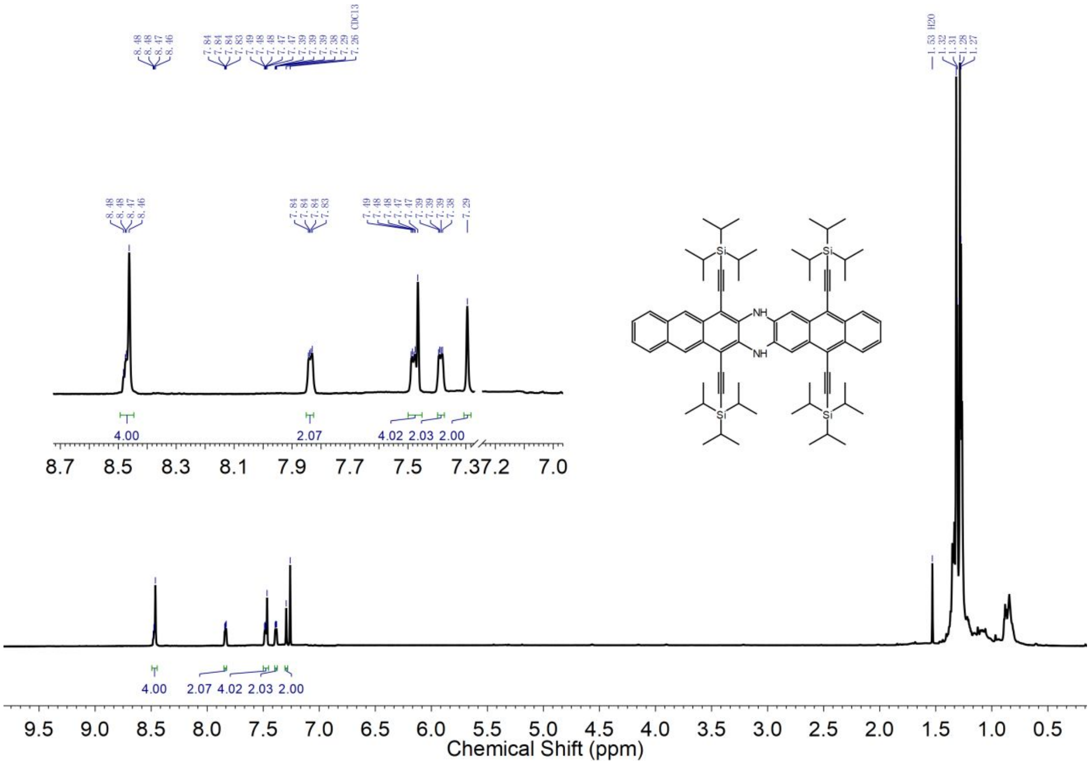

Figure S38. ^{1}H NMR spectrum (600 MHz, 295 K) of **DAH2-H$_2$** in $CDCl_3$.

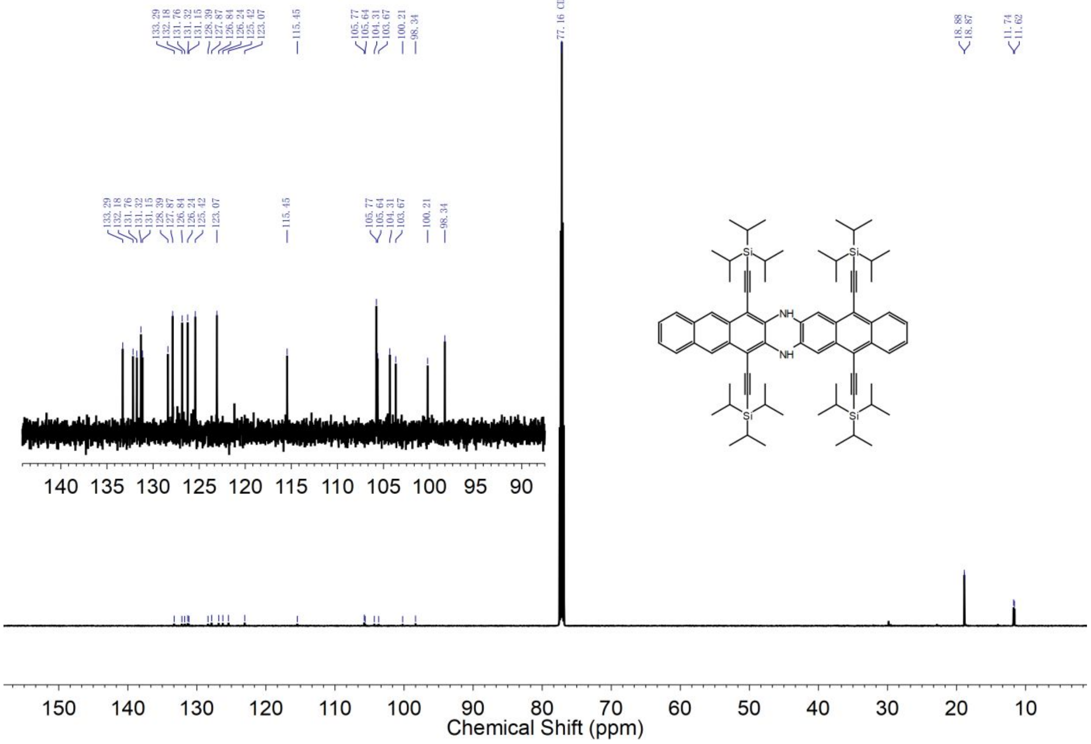

Figure S39. $^{13}C\{^1H\}$ NMR spectrum (151 MHz, 295 K) of **DAH2-H$_2$** in $CDCl_3$.

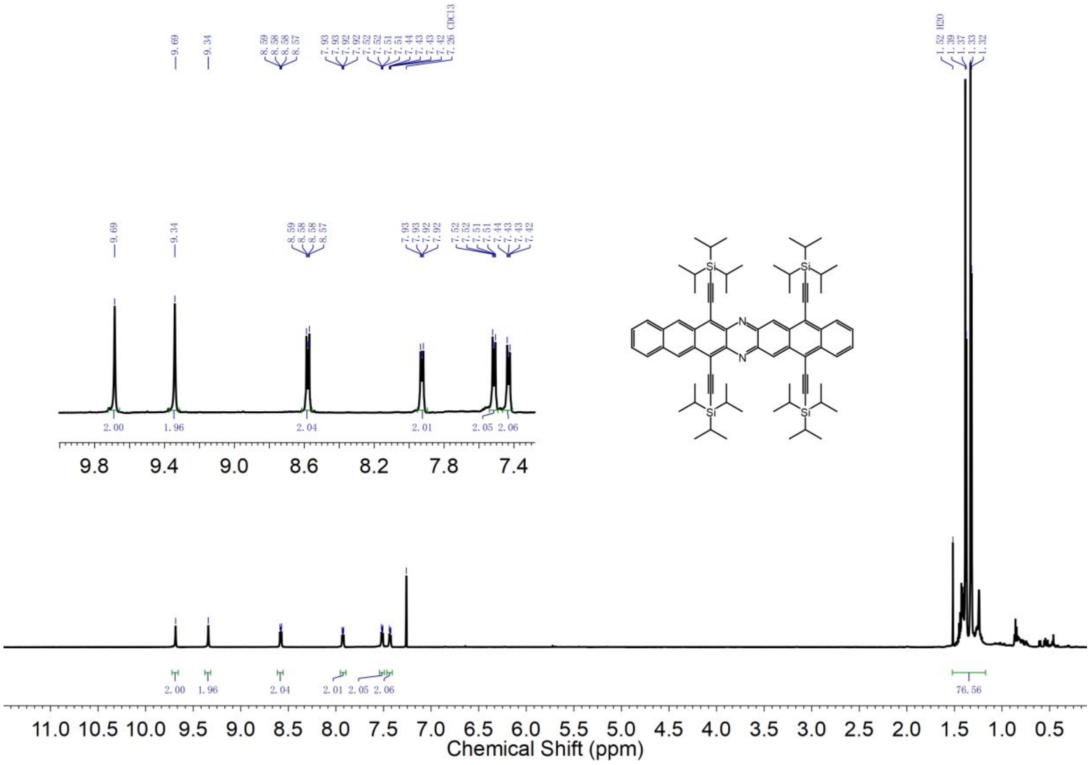

Figure S40. ^{1}H NMR spectrum (600 MHz, 295 K) of **DAH2** in $CDCl_3$.

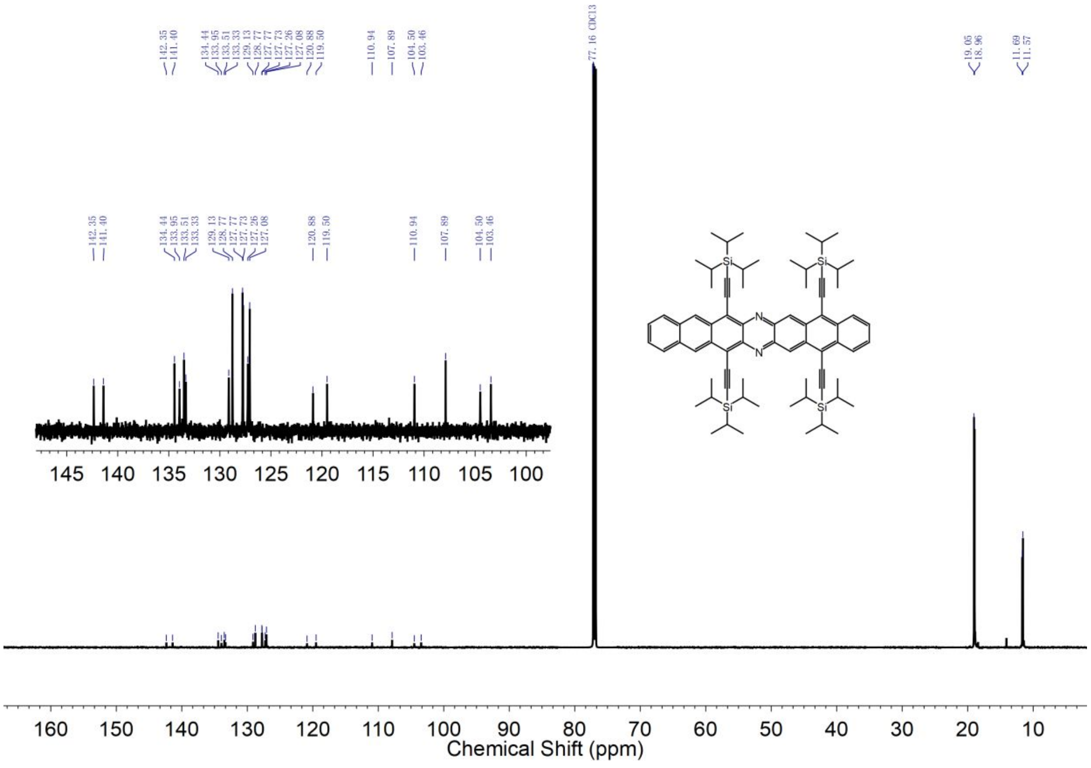

Figure S41. $^{13}C\{^{1}H\}$ NMR spectrum (151 MHz, 295 K) of **DAH2** in $CDCl_3$.

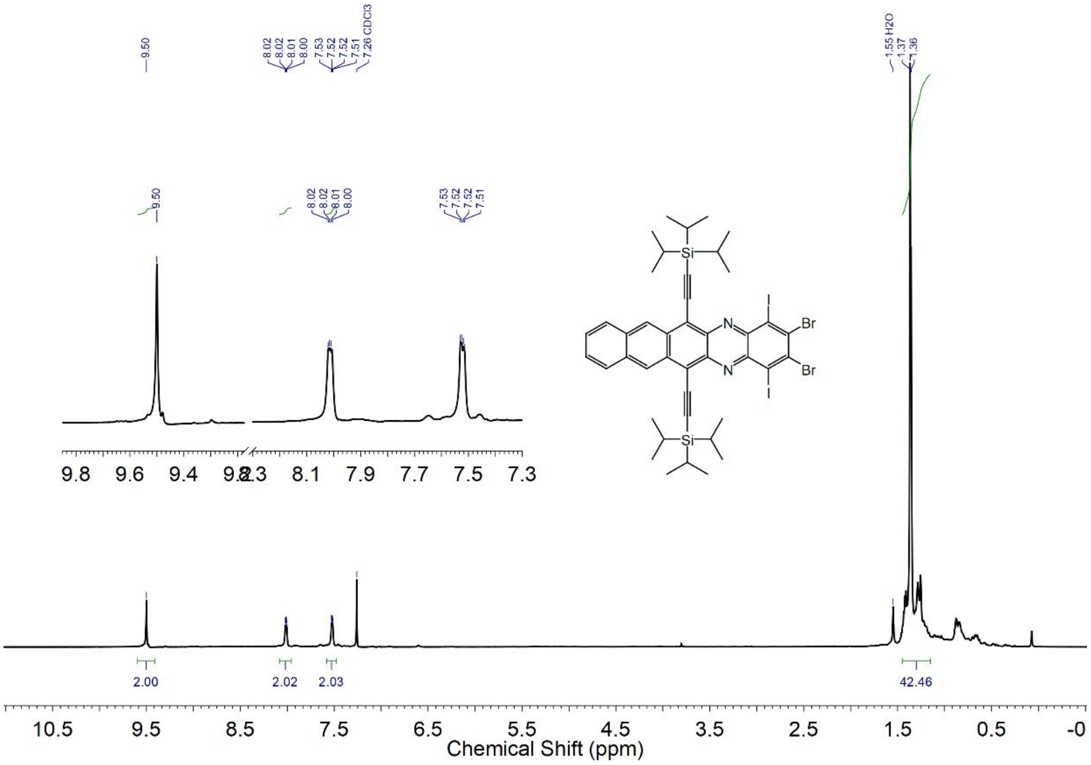

Figure S42. ^{1}H NMR spectrum (600 MHz, 295 K) of **5a** in $CDCl_3$.

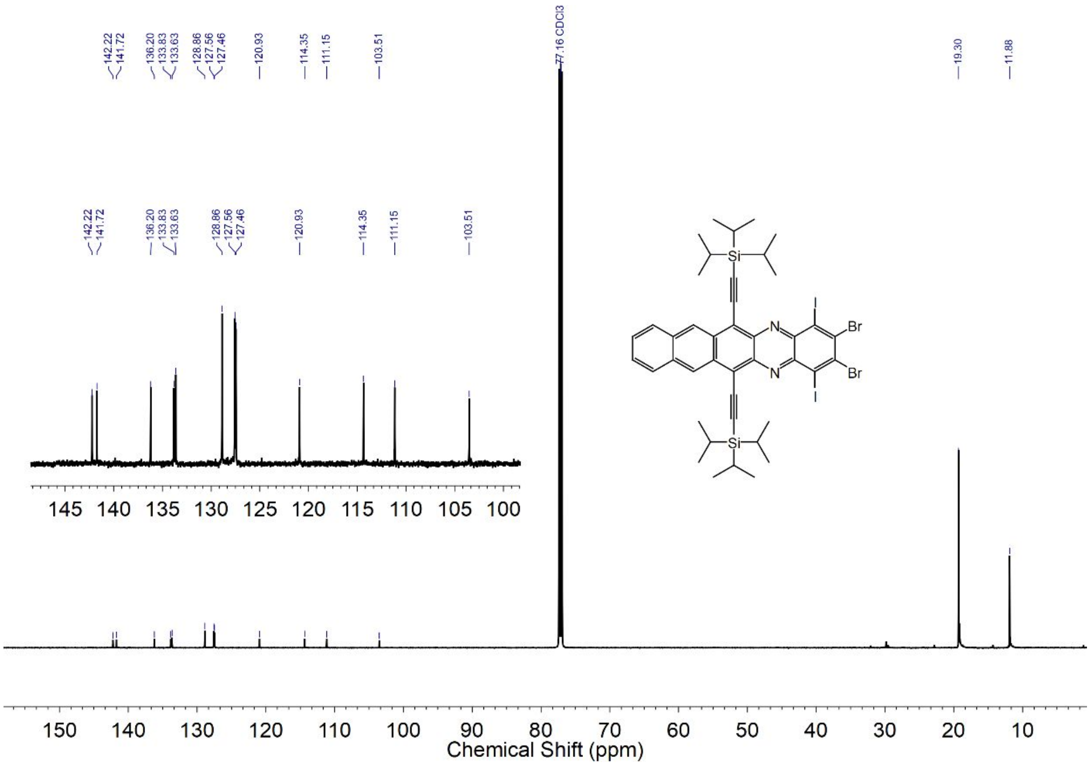

Figure S43. $^{13}C\{^{1}H\}$ NMR spectrum (151 MHz, 295 K) of **5a** in $CDCl_3$.

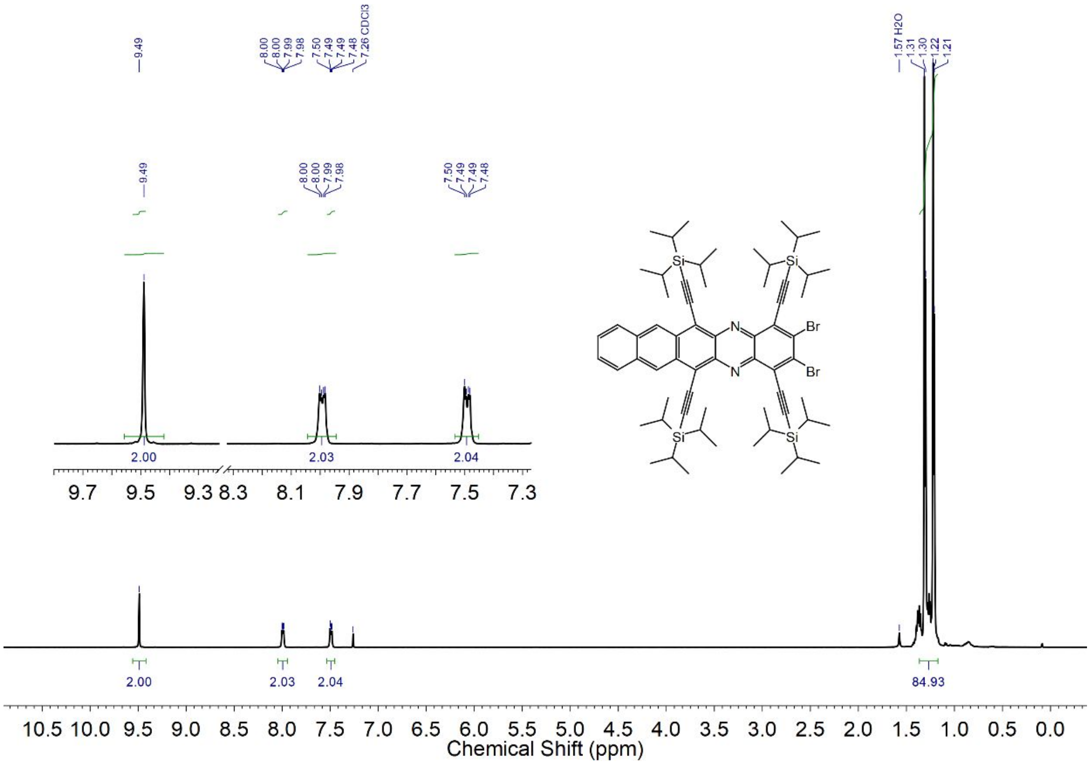

Figure S44. ^{1}H NMR spectrum (500 MHz, 295 K) of **6a** in $CDCl_3$.

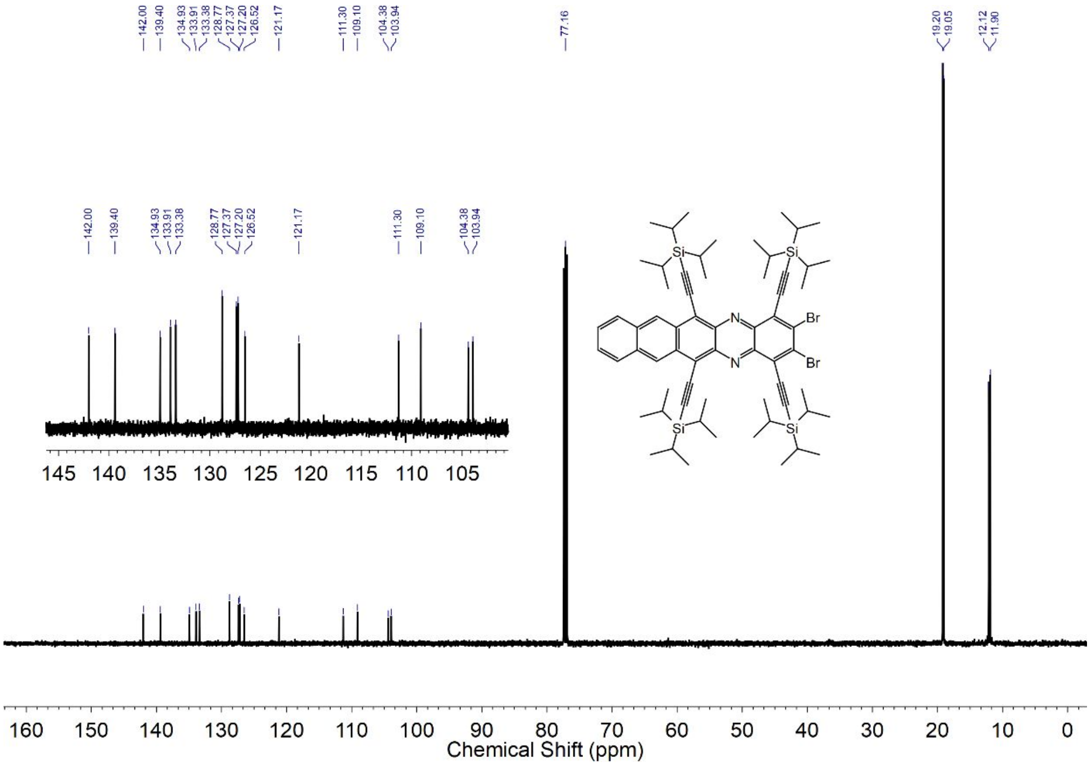

Figure S45. $^{13}C\{^{1}H\}$ NMR spectrum (126 MHz, 295 K) of **6a** in $CDCl_3$.

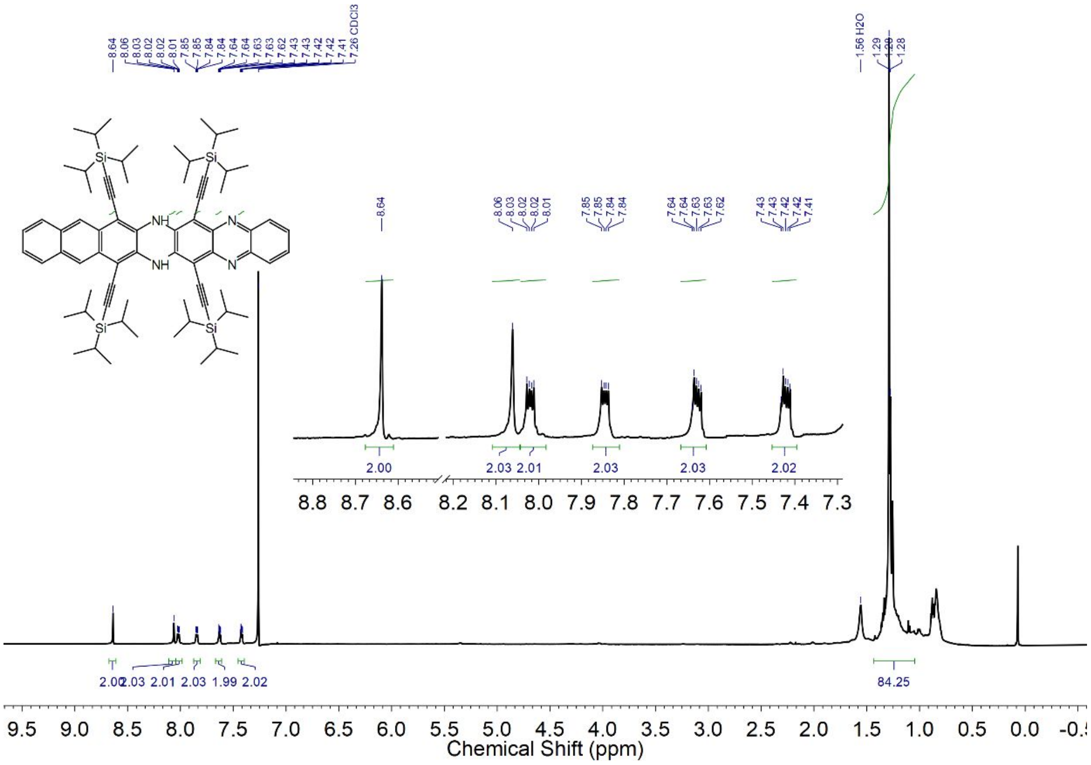

Figure S46. ^{1}H NMR spectrum (600 MHz, 295 K) of **TAH-H$_2$** in $CDCl_3$.

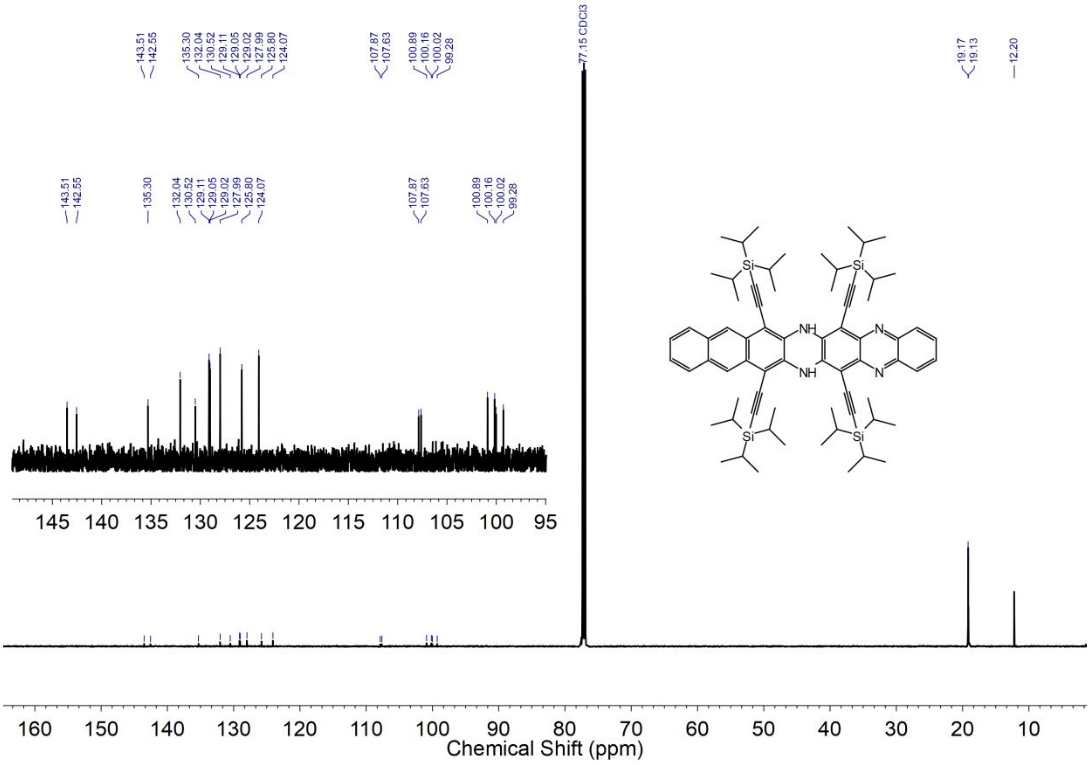

Figure S47. $^{13}C\{^{1}H\}$ NMR spectrum (151 MHz, 295 K) of **TAH-H$_2$** in $CDCl_3$.

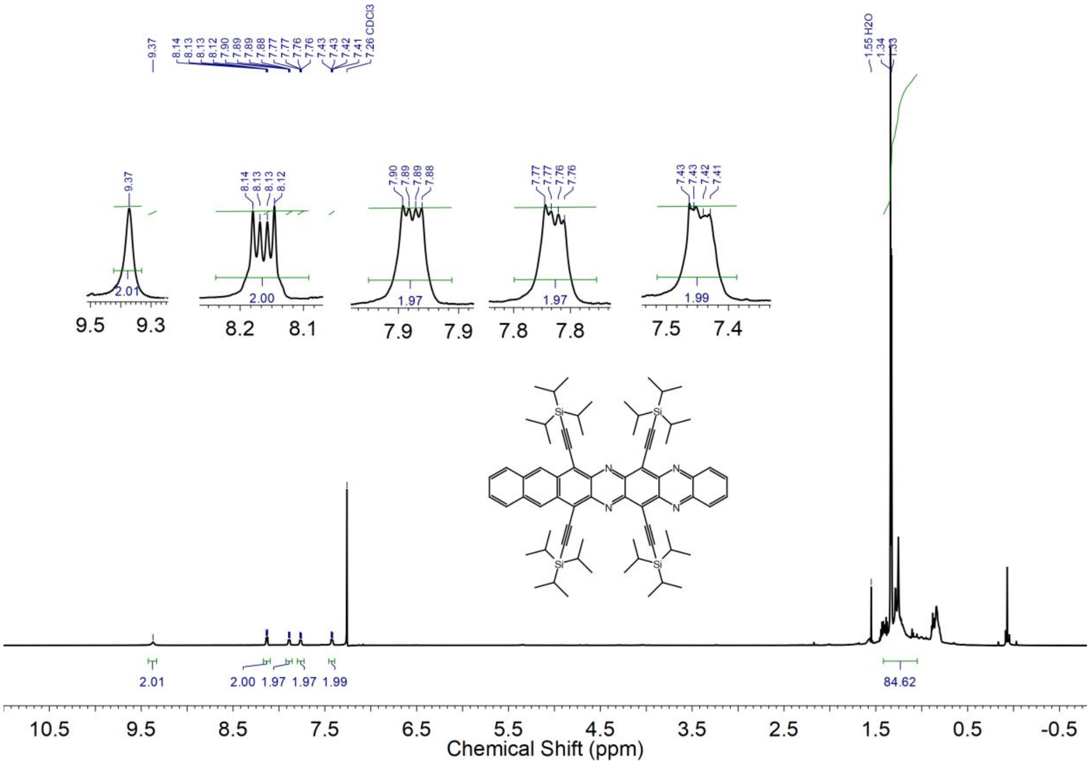

Figure S48. ^{1}H NMR spectrum (600 MHz, 295 K) of **TAH** in $CDCl_3$.

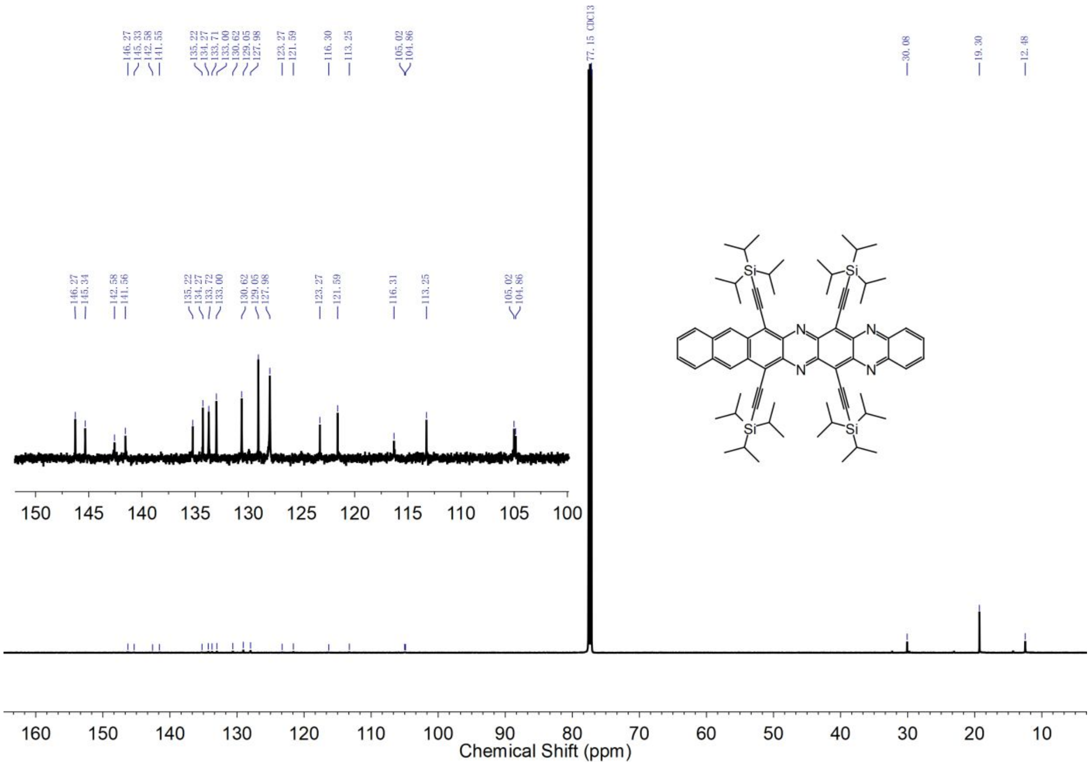

Figure S49. $^{13}C\{^{1}H\}$ NMR spectrum (151 MHz, 295 K) of **TAH** in $CDCl_3$.

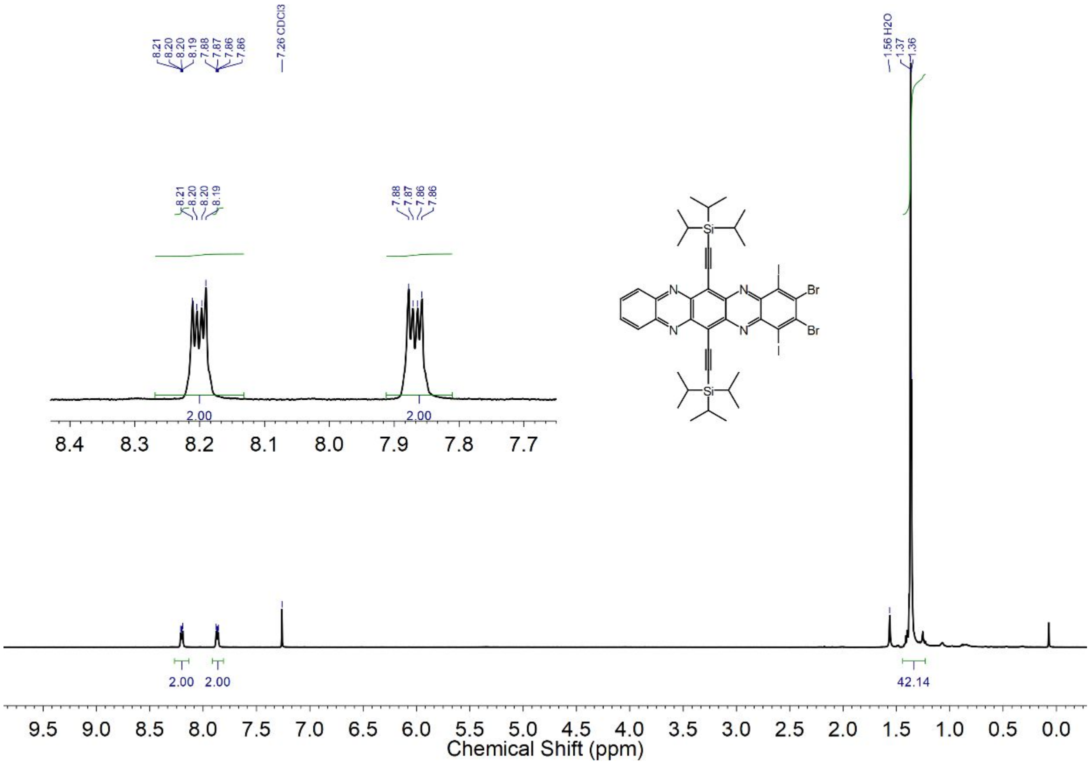

Figure S50. ^{1}H NMR spectrum (500 MHz, 295 K) of **5b** in $CDCl_3$.

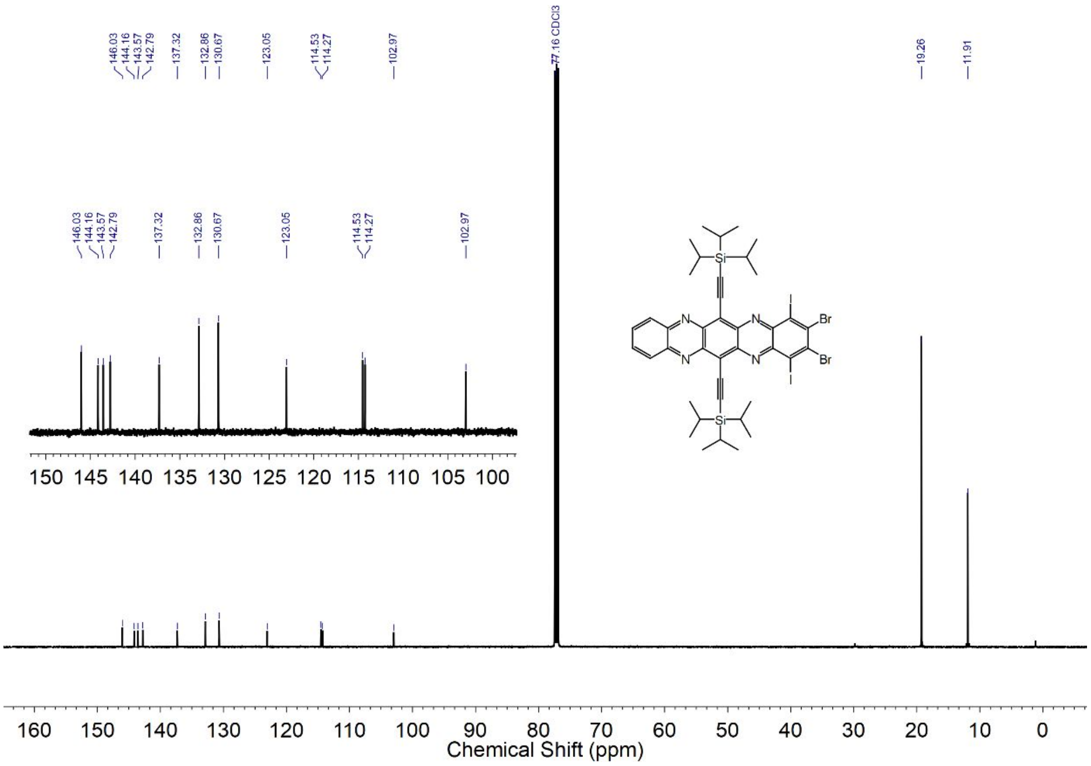

Figure S51. $^{13}C\{^1H\}$ NMR spectrum (126 MHz, 295 K) of **5b** in $CDCl_3$.

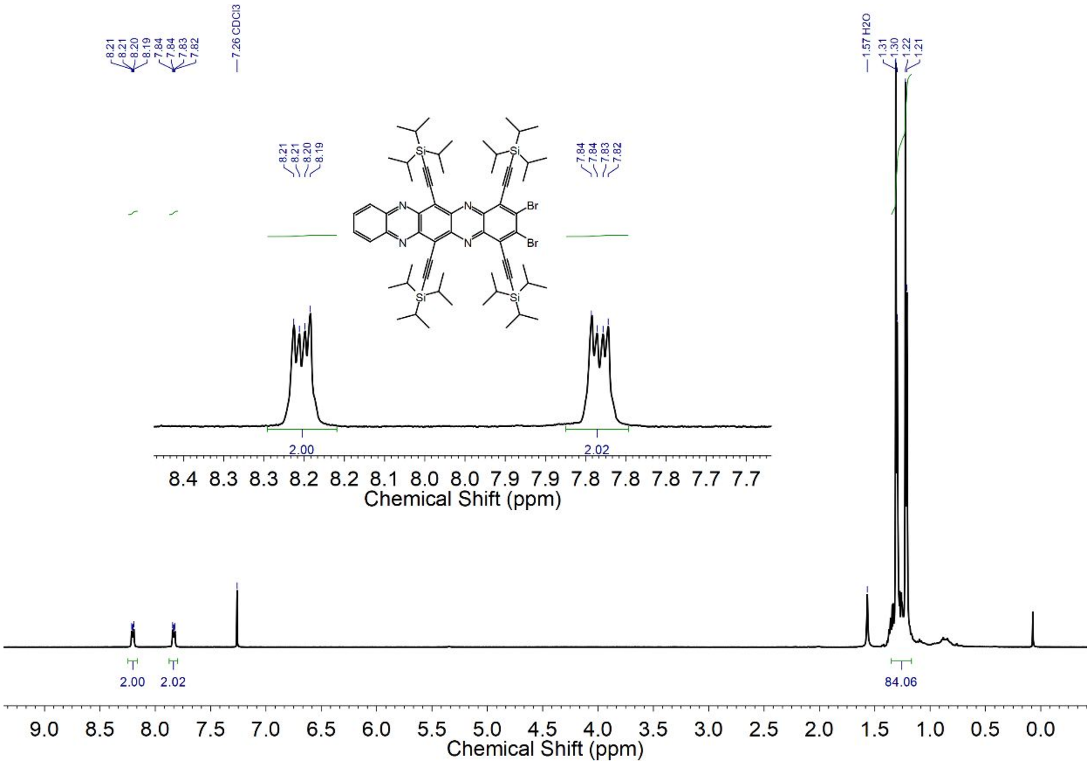

Figure S52. ^{1}H NMR spectrum (500 MHz, 295 K) of **6b** in $CDCl_3$.

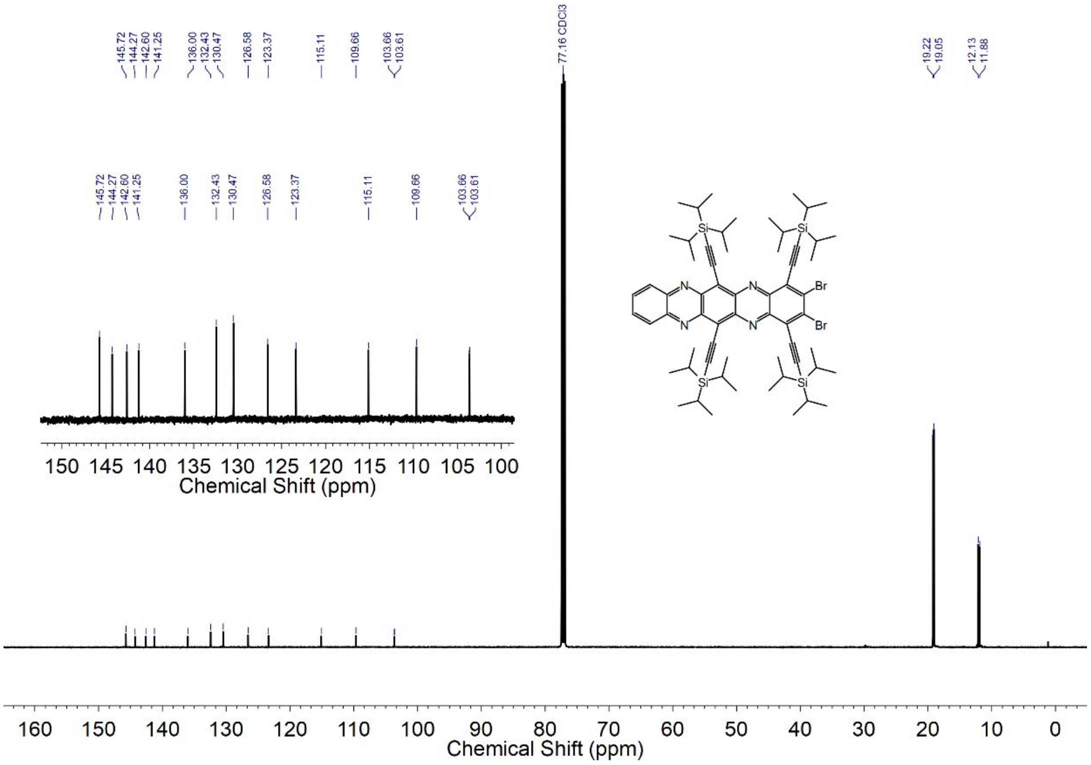

Figure S53. $^{13}C\{^{1}H\}$ NMR spectrum (126 MHz, 295 K) of **6b** in $CDCl_3$.

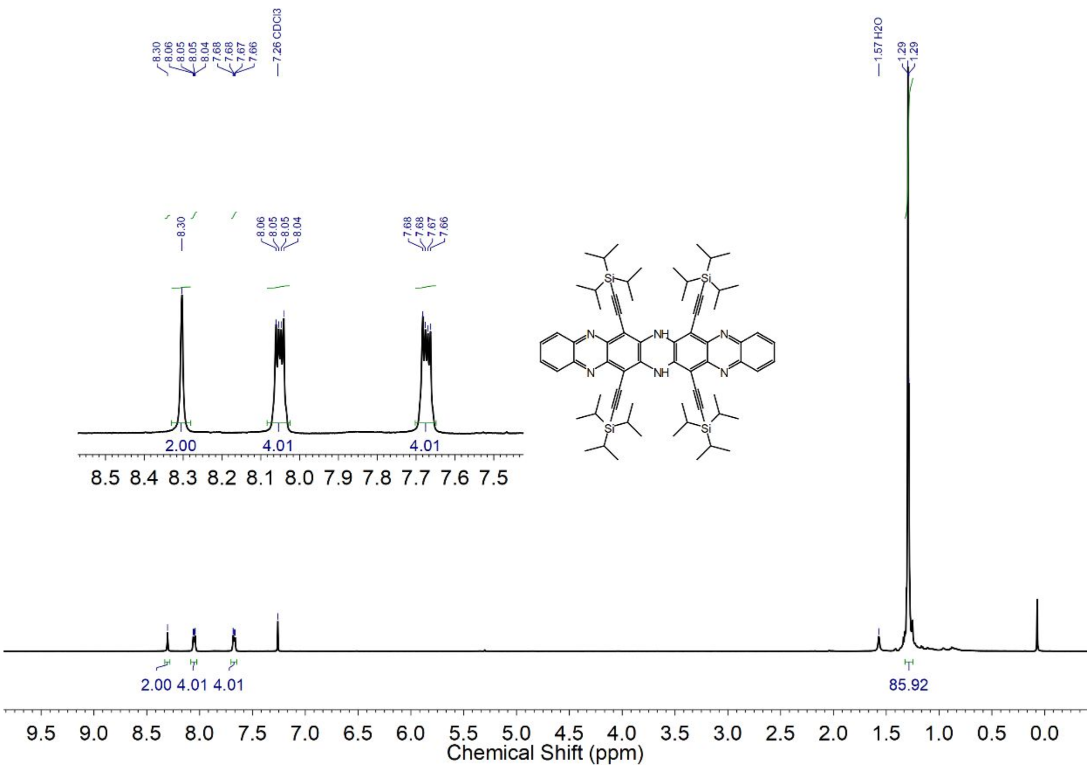

Figure S54. ^{1}H NMR spectrum (500 MHz, 295 K) of **HAH-H$_2$** in $CDCl_3$.

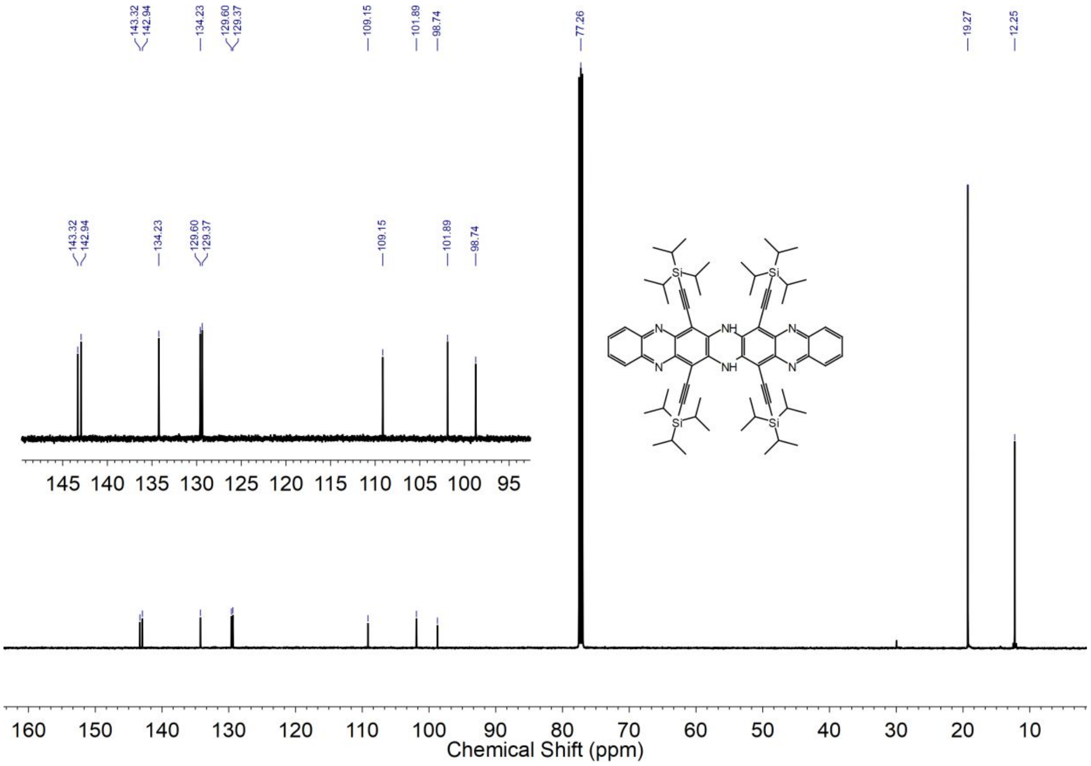

Figure S55. $^{13}C\{^{1}H\}$ NMR spectrum (126 MHz, 295 K) of **HAH-H$_2$** in $CDCl_3$.

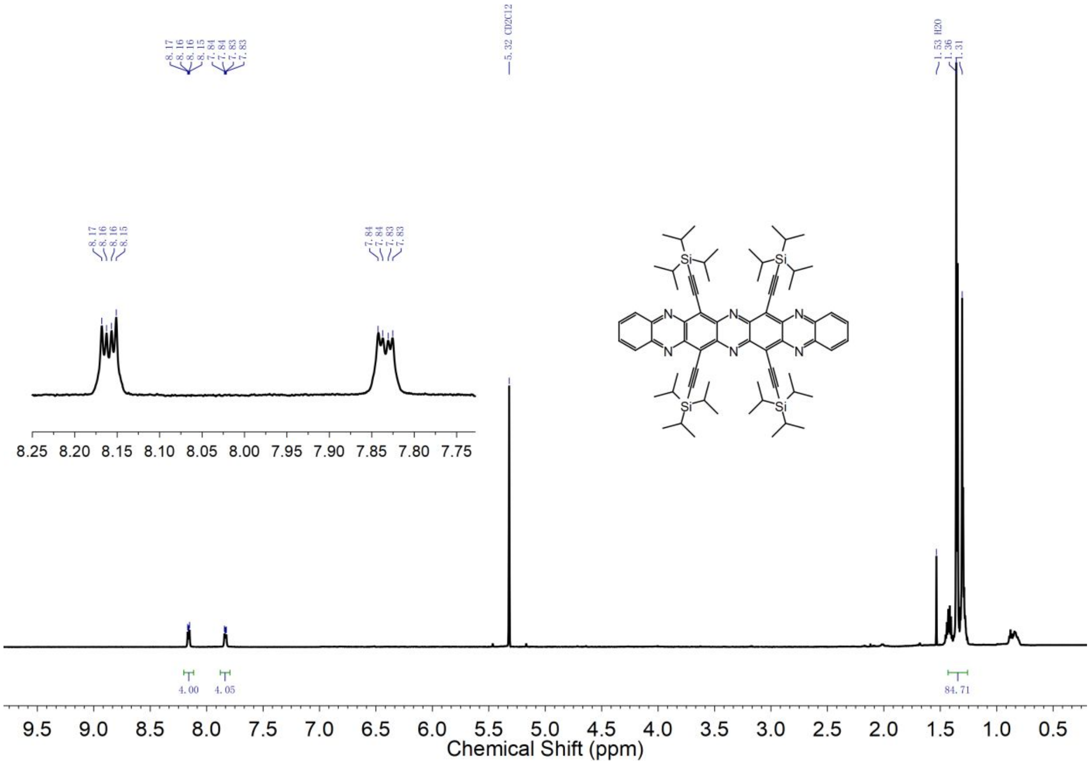

Figure S56. ^{1}H NMR spectrum (600 MHz, 295 K) of **HAH** in CD_2Cl_2 with PbO_2 under an inert atmosphere.

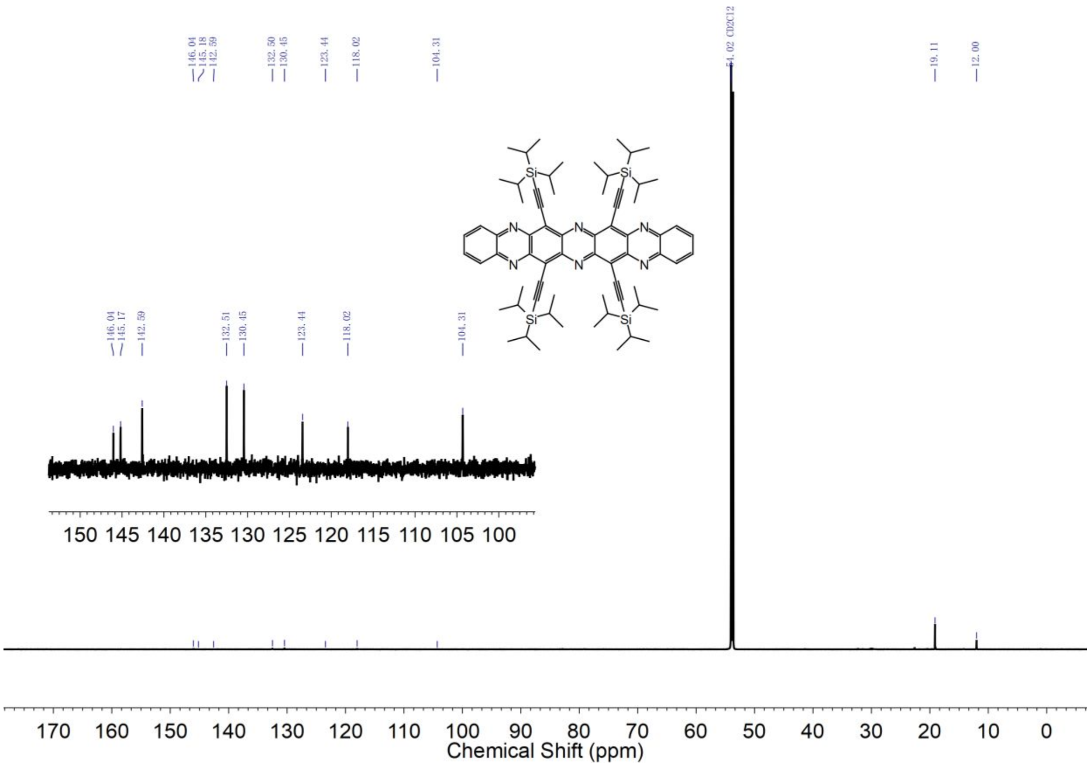

Figure S57. $^{13}C\{^1H\}$ NMR spectrum (151 MHz, 295 K) of **HAH** in CD_2Cl_2 with PbO_2 under an inert atmosphere.

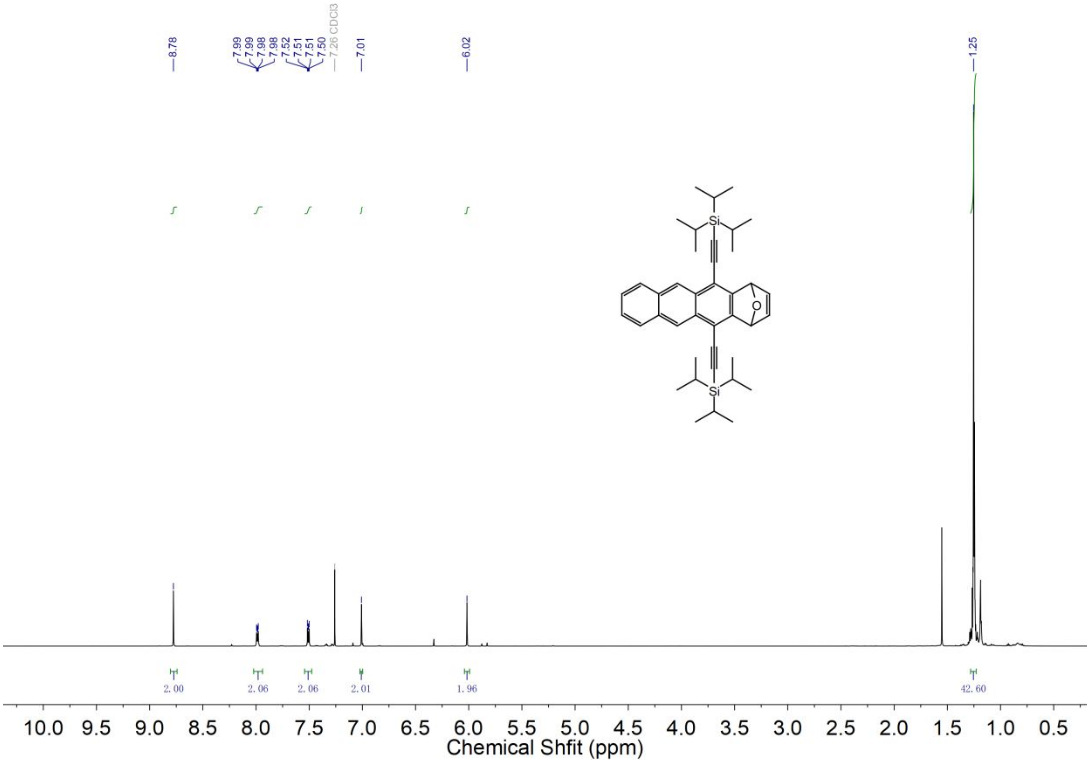

Figure S58. ^{1}H NMR spectrum (600 MHz, 295 K) of **S2** in $CDCl_3$.

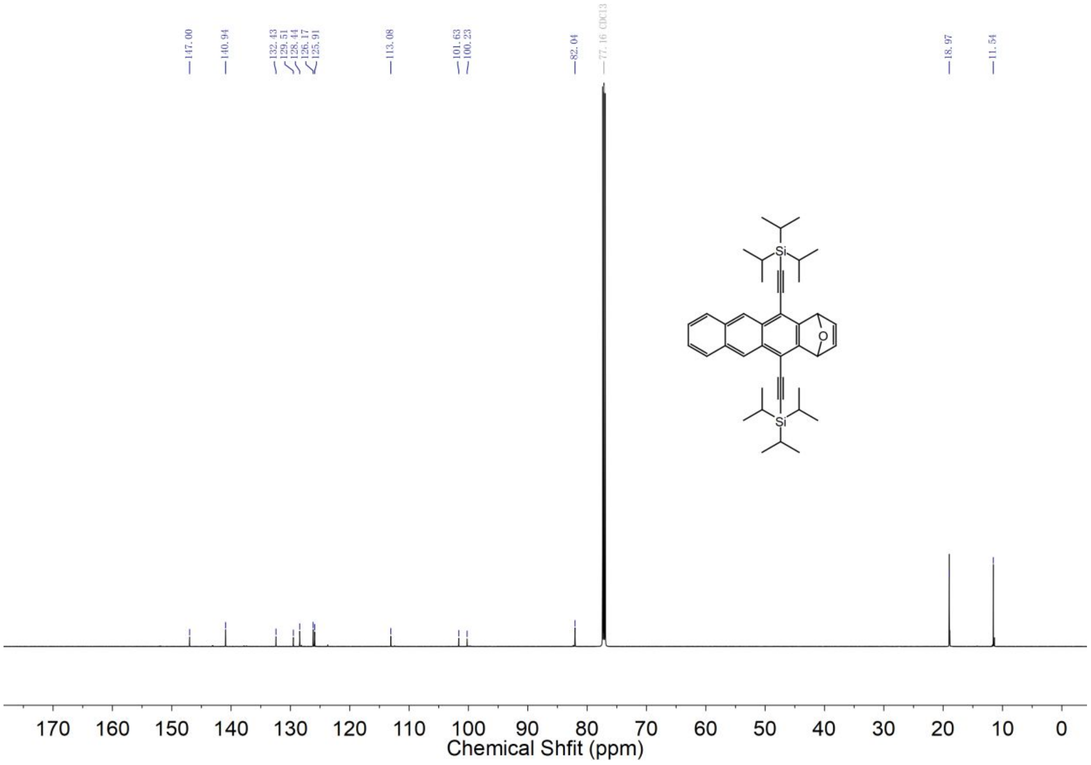

Figure S59. $^{13}C\{^{1}H\}$ NMR spectrum (151 MHz, 295 K) of **S2** in $CDCl_3$.

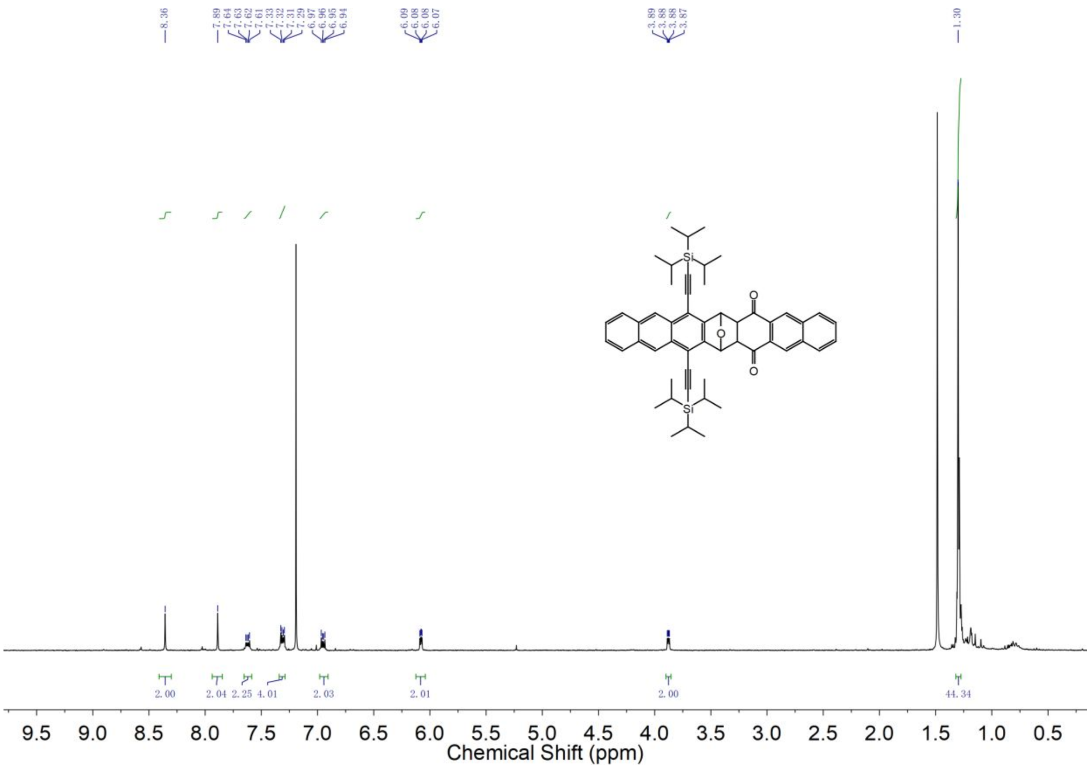

Figure S60. ^{1}H NMR spectrum (300 MHz, 295 K) of **S3** in $CDCl_3$.

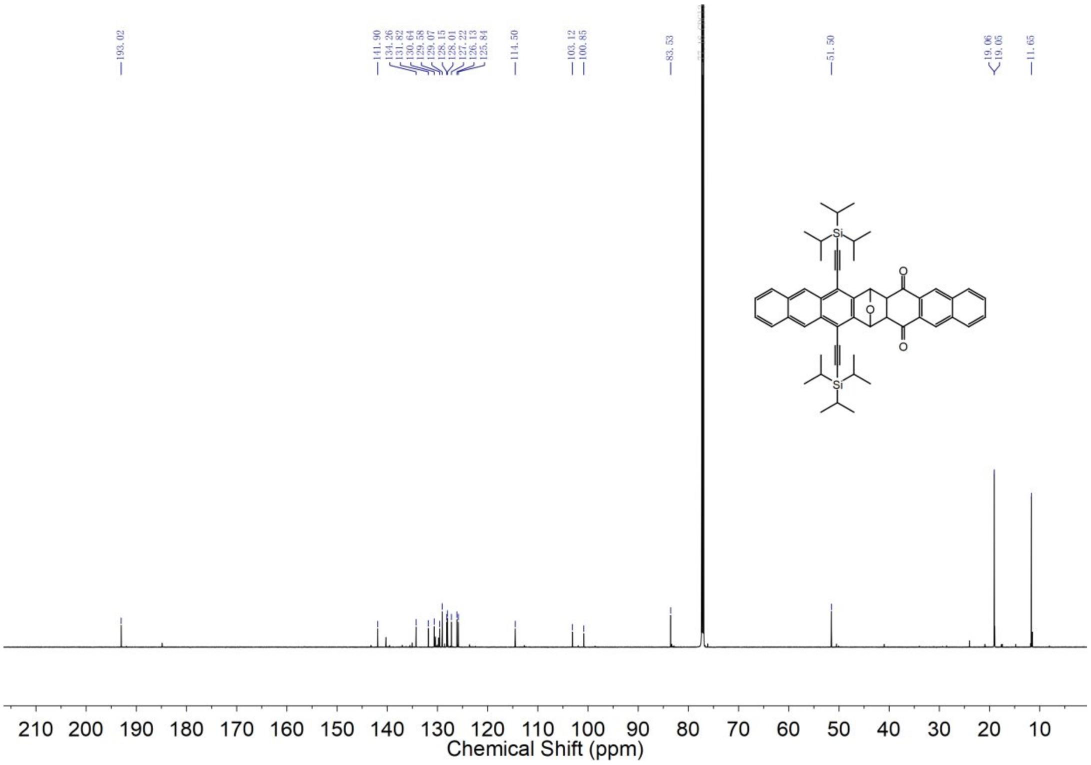

Figure S61. $^{13}C\{^{1}H\}$ NMR spectrum (176 MHz, 295 K) of **S3** in $CDCl_3$.

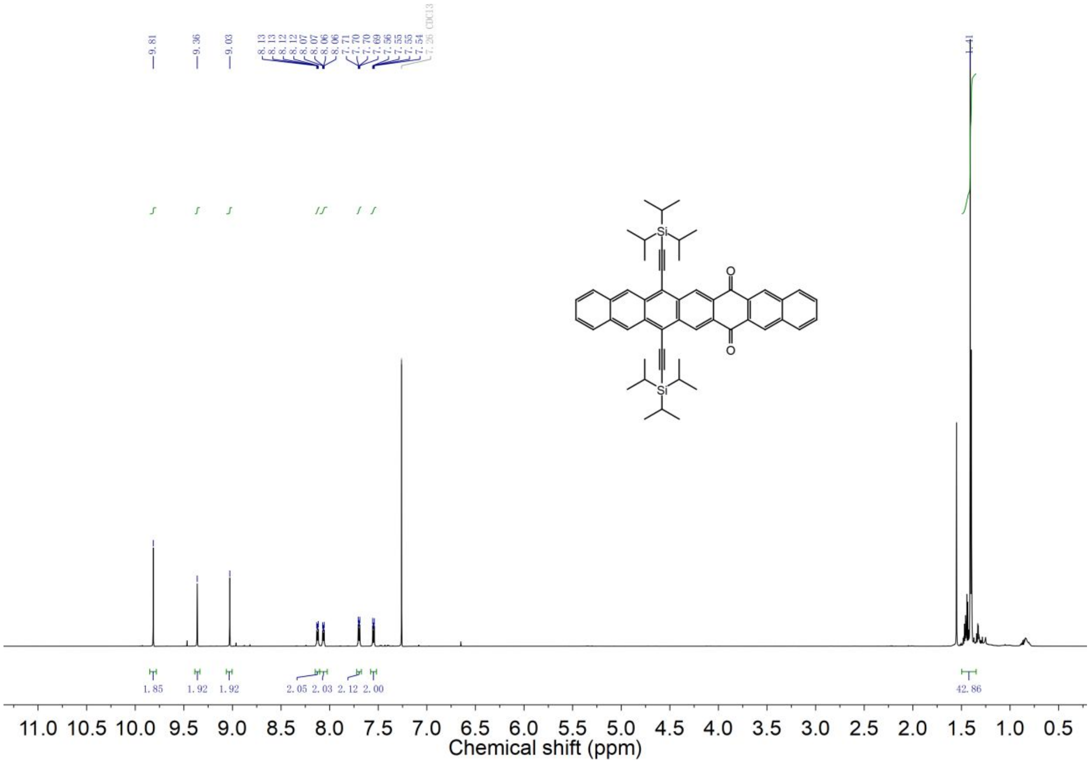

Figure S62. ^{1}H NMR spectrum (600 MHz, 295 K) of **S4** in $CDCl_3$.

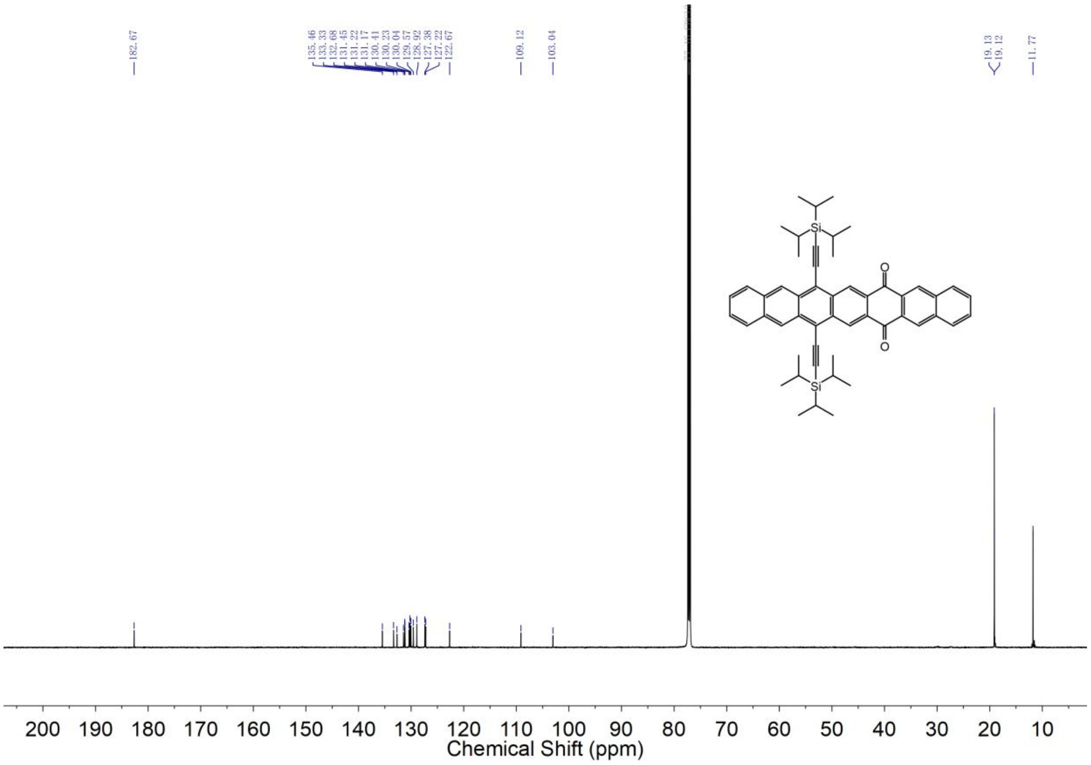

Figure S63. $^{13}C\{^{1}H\}$ NMR spectrum (151 MHz, 295 K) of **S4** in $CDCl_3$.

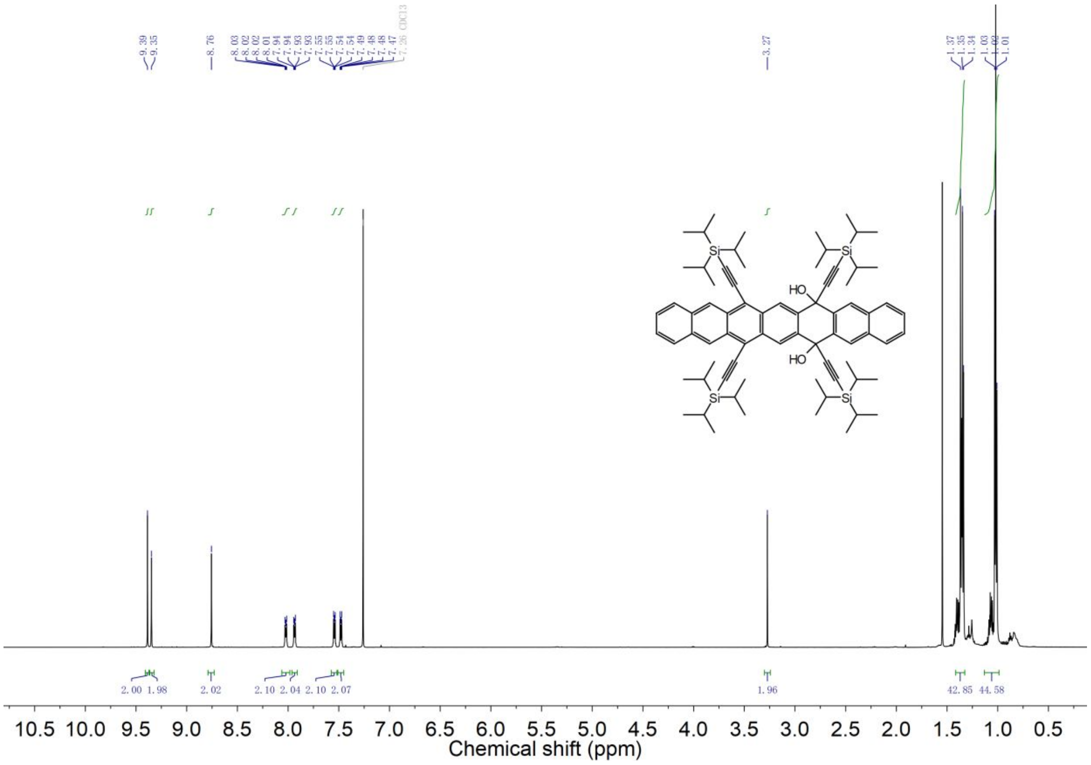

Figure S64. ^{1}H NMR spectrum (600 MHz, 295 K) of **S5** in $CDCl_3$.

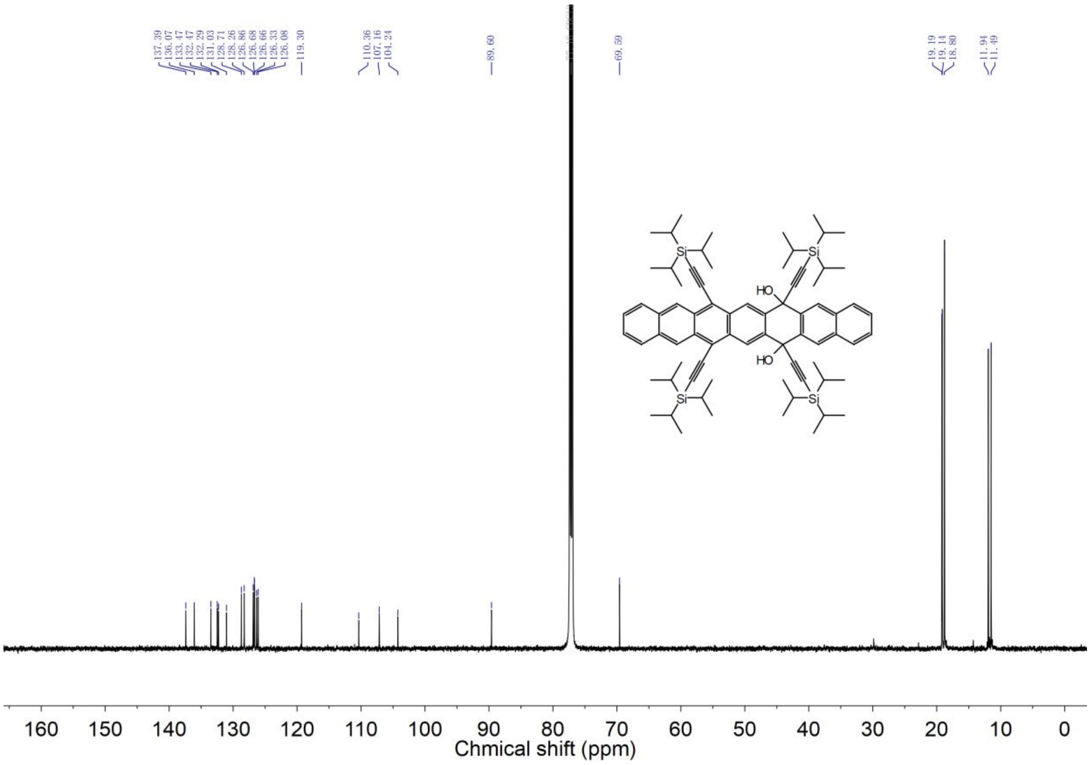

Figure S65. $^{13}C\{^1H\}$ NMR spectrum (151 MHz, 295 K) of **S5** in $CDCl_3$.

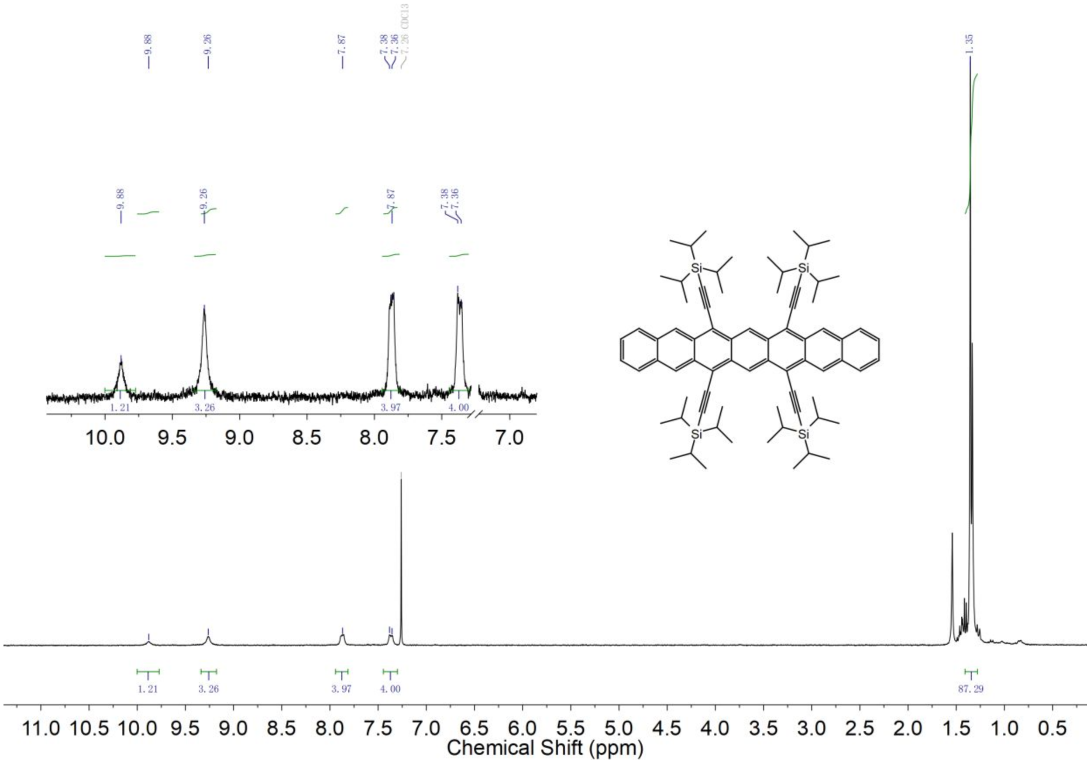

Figure S66. ^{1}H NMR spectrum (301 MHz, 295 K) of **Hep** in $CDCl_3$.

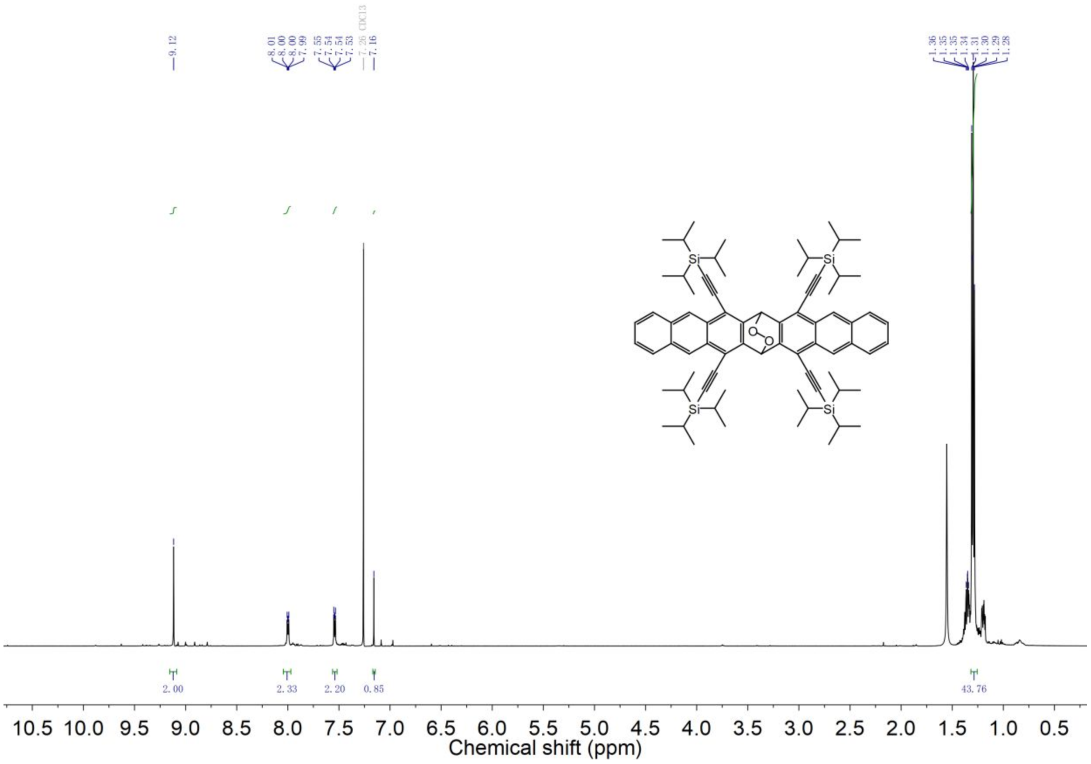

Figure S67. ^{1}H NMR spectrum (600 MHz, 295 K) of **S6** in $CDCl_3$.

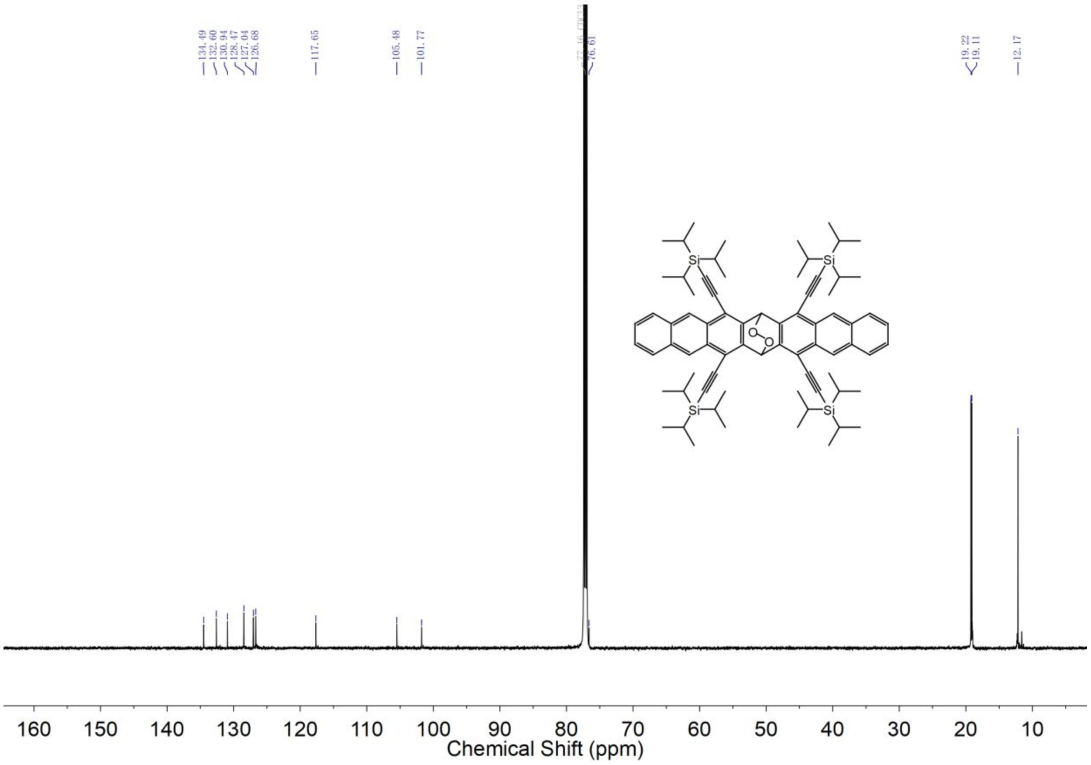

Figure S68. $^{13}C\{^1H\}$ NMR spectrum (151 MHz, 295 K) of **S6** in $CDCl_3$.

14. Crystallographic Data

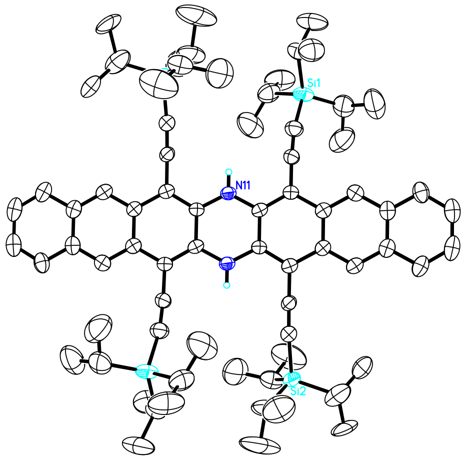

Table S8. Crystal data and structure refinement for **DAH1-H$_2$**.

CCDC number	2168603	
Empirical formula	$C_{72}H_{98}N_2Si_4$	
Formula weight	1103.88	
Temperature	200(2) K	
Wavelength	0.71073 Å	
Crystal system	monoclinic	
Space group	$P2_1/n$	
Z	2	
Unit cell dimensions	a = 9.3027(7) Å	α = 90 deg.
	b = 17.6983(13) Å	β = 95.7216(19) deg.
	c = 20.5902(16) Å	γ = 90 deg.
Volume	3373.1(4) Å^3	
Density (calculated)	1.09 g/cm^3	
Absorption coefficient	0.13 mm^{-1}	
Crystal shape	column	
Crystal size	0.278 x 0.055 x 0.033 mm^3	
Crystal colour	orange	
Theta range for data collection	1.5 to 23.9 deg.	
Index ranges	$-10\le h\le 10$, $-20\le k\le 19$, $-23\le l\le 23$	
Reflections collected	28485	
Independent reflections	5208 (R(int) = 0.0728)	
Observed reflections	3298 ($I > 2\sigma(I)$)	
Absorption correction	Semi-empirical from equivalents	
Max. and min. transmission	0.96 and 0.92	
Refinement method	Full-matrix least-squares on F^2	
Data/restraints/parameters	5208 / 731 / 419	
Goodness-of-fit on F^2	1.02	
Final R indices (I>2sigma(I))	R1 = 0.062, wR2 = 0.149	
Largest diff. peak and hole	0.38 and -0.26 eÅ^{-3}	

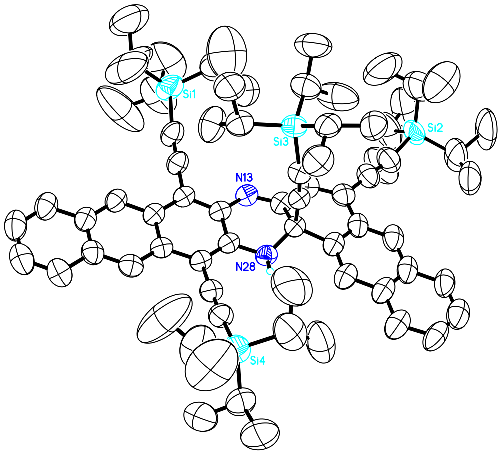

Table S9. Crystal data and structure refinement for **S1**.

CCDC number	2168604	
Empirical formula	$C_{72}H_{98}N_2Si_4$	
Formula weight	1103.88	
Temperature	200(2) K	
Wavelength	1.54178 Å	
Crystal system	triclinic	
Space group	P $\bar{1}$	
Z	2	
Unit cell dimensions	a = 13.797(2) Å	α =104.09(1) deg.
	b = 14.614(2) Å	β =105.877(11) deg.
	c = 18.584(2) Å	γ = 94.914(11) deg.
Volume	3448.9(9) $Å^3$	
Density (calculated)	1.06 g/cm^3	
Absorption coefficient	1.09 mm^{-1}	
Crystal shape	brick	
Crystal size	0.105 x 0.042 x 0.030 mm^3	
Crystal colour	dark orange	
Theta range for data collection	3.4 to 59.3 deg.	
Index ranges	$-14 \leq h \leq 15$, $-13 \leq k \leq 15$, $-18 \leq l \leq 20$	
Reflections collected	23298	
Independent reflections	9374 (R(int) = 0.0412)	
Observed reflections	5145 ($I > 2\sigma(I)$)	
Absorption correction	Semi-empirical from equivalents	
Max. and min. transmission	0.97 and 0.57	
Refinement method	Full-matrix least-squares on F^2	
Data/restraints/parameters	9374 / 2118 / 747	
Goodness-of-fit on F^2	1.03	
Final R indices (I>2sigma(I))	R1 = 0.089, wR2 = 0.235	
Largest diff. peak and hole	0.37 and -0.28 eÅ^{-3}	

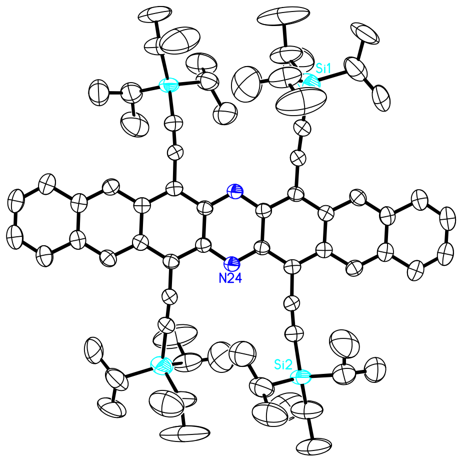

Table S10.Crystal data and structure refinement for **DAH1**.

CCDC number	2168605	
Empirical formula	$C_{72}H_{96}N_2Si_4$	
Formula weight	1101.86	
Temperature	200(2) K	
Wavelength	1.54178 Å	
Crystal system	monoclinic	
Space group	$P2_1/n$	
Z	2	
Unit cell dimensions	a = 9.1368(2) Å	α = 90 deg.
	b = 17.9587(5) Å	β = 95.607(2) deg.
	c = 20.6347(4) Å	γ = 90 deg.
Volume	3369.65(14) $Å^3$	
Density (calculated)	1.09 g/cm^3	
Absorption coefficient	1.11 mm^{-1}	
Crystal shape	column	
Crystal size	0.430 x 0.032 x 0.030 mm^3	
Crystal colour	dark green	
Theta range for data collection	3.3 to 71.6 deg.	
Index ranges	$-11 \le h \le 7$, $-20 \le k \le 21$, $-23 \le l \le 25$	
Reflections collected	23373	
Independent reflections	6348 (R(int) = 0.0438)	
Observed reflections	3881 ($I > 2\sigma(I)$)	
Absorption correction	Semi-empirical from equivalents	
Max. and min. transmission	0.96 and 0.87	
Refinement method	Full-matrix least-squares on F^2	
Data/restraints/parameters	6348 / 729 / 403	
Goodness-of-fit on F^2	1.03	
Final R indices (I>2sigma(I))	R1 = 0.062, wR2 = 0.160	
Largest diff. peak and hole	0.49 and -0.35 $eÅ^{-3}$	

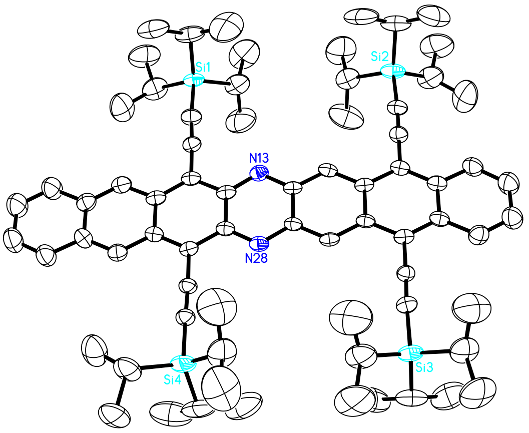

Table S11. Crystal data and structure refinement for **DAH2**.

CCDC number	2254467
Empirical formula	$C_{72.50}H_{97}ClN_2Si_4$
Formula weight	1144.33
Temperature	200(2) K
Wavelength	0.71073 Å
Crystal system	triclinic
Space group	P $\bar{1}$
Z	4
Unit cell dimensions	a = 15.0283(15) Å α = 79.290(3) deg.
	b = 15.0685(15) Å β = 79.201(3) deg.
	c = 32.471(3) Å γ = 89.738(3) deg.
Volume	7093.7(12) $Å^3$
Density (calculated)	1.07 g/cm^3
Absorption coefficient	0.16 mm^{-1}
Crystal shape	plate
Crystal size	0.195 x 0.168 x 0.022 mm^3
Crystal colour	green
Theta range for data collection	0.6 to 22.0 deg.
Index ranges	$-15 \le h \le 15$, $-15 \le k \le 15$, $-34 \le l \le 34$
Reflections collected	52446
Independent reflections	17341 (R(int) = 0.0945)
Observed reflections	8726 ($I > 2\sigma(I)$)
Absorption correction	Semi-empirical from equivalents
Max. and min. transmission	0.96 and 0.89
Refinement method	Full-matrix least-squares on F^2
Data/restraints/parameters	17341 / 8997 / 1705
Goodness-of-fit on F^2	1.03
Final R indices (I>2sigma(I))	R1 = 0.088, wR2 = 0.199
Largest diff. peak and hole	0.43 and -0.61 $eÅ^{-3}$

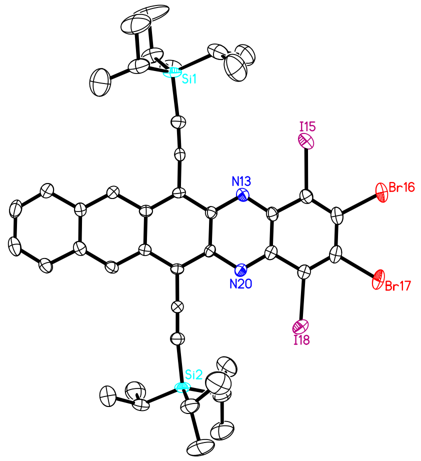

Table S12. Crystal data and structure refinement for **5a**.

CCDC number	2081160	
Empirical formula	$C_{42}H_{48}Br_2I_2N_2Si_2$	
Formula weight	1050.62	
Temperature	200(2) K	
Wavelength	0.71073 Å	
Crystal system	monoclinic	
Space group	$P2_1/c$	
Z	4	
Unit cell dimensions	a = 8.6096(4) Å	α = 90 deg.
	b = 35.6295(17) Å	β = 101.6028(12) deg.
	c = 14.4632(8) Å	γ = 90 deg.
Volume	4346.0(4) $Å^3$	
Density (calculated)	1.61 g/cm^3	
Absorption coefficient	3.37 mm^{-1}	
Crystal shape	plate	
Crystal size	0.232 x 0.093 x 0.016 mm^3	
Crystal colour	green	
Theta range for data collection	1.5 to 28.4 deg.	
Index ranges	$-11 \leq h \leq 11$, $-45 \leq k \leq 47$, $-18 \leq l \leq 18$	
Reflections collected	47770	
Independent reflections	10074 (R(int) = 0.0610)	
Observed reflections	6476 ($I > 2\sigma(I)$)	
Absorption correction	Semi-empirical from equivalents	
Max. and min. transmission	0.96 and 0.85	
Refinement method	Full-matrix least-squares on F^2	
Data/restraints/parameters	10074 / 0 / 480	
Goodness-of-fit on F^2	1.02	
Final R indices (I>2sigma(I))	R1 = 0.044, wR2 = 0.087	
Largest diff. peak and hole	0.78 and -0.72 $eÅ^{-3}$	

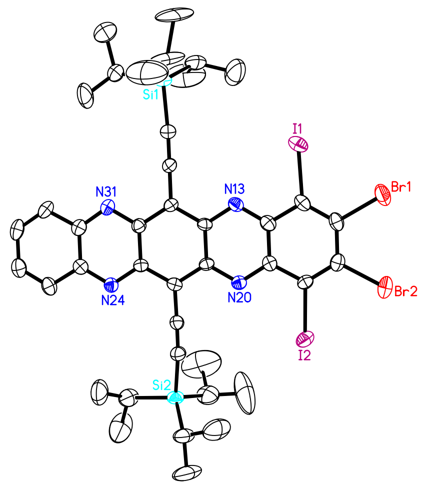

Table S13. Crystal data and structure refinement for **5b**.

CCDC number	2081161	
Empirical formula	$C_{40}H_{46}Br_2I_2N_4Si_2$	
Formula weight	1052.61	
Temperature	200(2) K	
Wavelength	0.71073 Å	
Crystal system	monoclinic	
Space group	$P2_1/c$	
Z	4	
Unit cell dimensions	a = 19.1981(13) Å	α = 90 deg.
	b = 13.9124(10) Å	β = 111.1688(15) deg.
	c = 17.1871(12) Å	γ = 90 deg.
Volume	4280.8(5) $Å^3$	
Density (calculated)	1.63 g/cm^3	
Absorption coefficient	3.42 mm^{-1}	
Crystal shape	plate	
Crystal size	0.242 x 0.050 x 0.016 mm^3	
Crystal colour	green	
Theta range for data collection	1.9 to 28.6 deg.	
Index ranges	$-25 \le h \le 25$, $-18 \le k \le 18$, $-22 \le l \le 22$	
Reflections collected	49039	
Independent reflections	10609 (R(int) = 0.0690)	
Observed reflections	6249 ($I > 2\sigma(I)$)	
Absorption correction	Semi-empirical from equivalents	
Max. and min. transmission	0.96 and 0.83	
Refinement method	Full-matrix least-squares on F^2	
Data/restraints/parameters	10609 / 627 / 523	
Goodness-of-fit on F^2	1.00	
Final R indices (I>2sigma(I))	R1 = 0.044, wR2 = 0.077	
Largest diff. peak and hole	0.71 and -0.96 $eÅ^{-3}$	

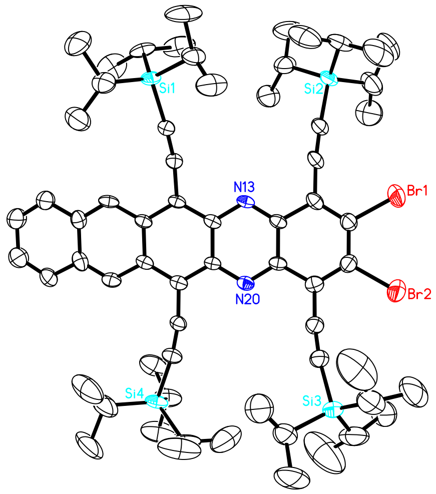

Table S14. Crystal data and structure refinement for **6a**.

CCDC number	2081162	
Empirical formula	$C_{64}H_{90}Br_2N_2Si_4$	
Formula weight	1159.55	
Temperature	200(2) K	
Wavelength	0.71073 Å	
Crystal system	monoclinic	
Space group	$P2_1/c$	
Z	4	
Unit cell dimensions	a = 28.443(2) Å	α = 90 deg.
	b = 15.1439(11) Å	β = 102.959(2) deg.
	c = 15.3047(12) Å	γ = 90 deg.
Volume	6424.4(8) $Å^3$	
Density (calculated)	1.20 g/cm^3	
Absorption coefficient	1.37 mm^{-1}	
Crystal shape	plate	
Crystal size	0.113 x 0.106 x 0.012 mm^3	
Crystal colour	green	
Theta range for data collection	0.7 to 21.5 deg.	
Index ranges	$-29 \leq h \leq 29$, $-15 \leq k \leq 15$, $-15 \leq l \leq 15$	
Reflections collected	44850	
Independent reflections	7326 (R(int) = 0.1067)	
Observed reflections	4387 ($I > 2\sigma(I)$)	
Absorption correction	Semi-empirical from equivalents	
Max. and min. transmission	0.97 and 0.88	
Refinement method	Full-matrix least-squares on F^2	
Data/restraints/parameters	7326 / 1887 / 722	
Goodness-of-fit on F^2	1.03	
Final R indices (I>2sigma(I))	R1 = 0.060, wR2 = 0.137	
Largest diff. peak and hole	0.83 and -0.83 $eÅ^{-3}$	

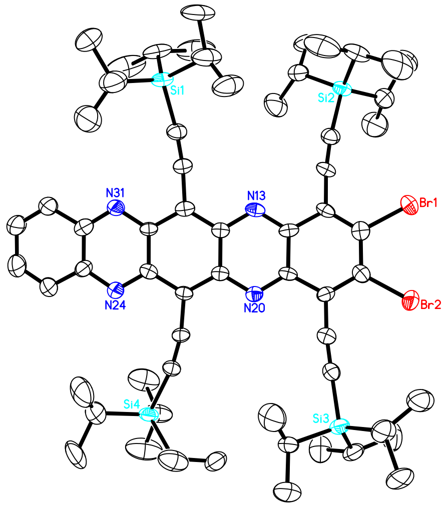

Table S15. Crystal data and structure refinement for **6b**.

CCDC number	2081163	
Empirical formula	$C_{62}H_{88}Br_2N_4Si_4$	
Formula weight	1161.54	
Temperature	200(2) K	
Wavelength	1.54178 Å	
Crystal system	monoclinic	
Space group	$P2_1/c$	
Z	4	
Unit cell dimensions	a = 29.2059(8) Å	α = 90 deg.
	b = 14.9414(4) Å	β =104.853(2) deg.
	c = 15.1021(4) Å	γ = 90 deg.
Volume	6370.0(3) $Å^3$	
Density (calculated)	1.21 g/cm^3	
Absorption coefficient	2.62 mm^{-1}	
Crystal shape	plate	
Crystal size	0.085 x 0.053 x 0.015 mm^3	
Crystal colour	green	
Theta range for data collection	4.2 to 60.0 deg.	
Index ranges	$-32\le h\le 31$, $-16\le k\le 11$, $-16\le l\le 12$	
Reflections collected	36257	
Independent reflections	9187 (R(int) = 0.1250)	
Observed reflections	5570 ($I > 2\sigma(I)$)	
Absorption correction	Semi-empirical from equivalents	
Max. and min. transmission	0.96 and 0.59	
Refinement method	Full-matrix least-squares on F^2	
Data/restraints/parameters	9187 / 0 / 682	
Goodness-of-fit on F^2	1.11	
Final R indices (I>2sigma(I))	R1 = 0.077, wR2 = 0.121	
Largest diff. peak and hole	0.42 and -0.37 $eÅ^{-3}$	

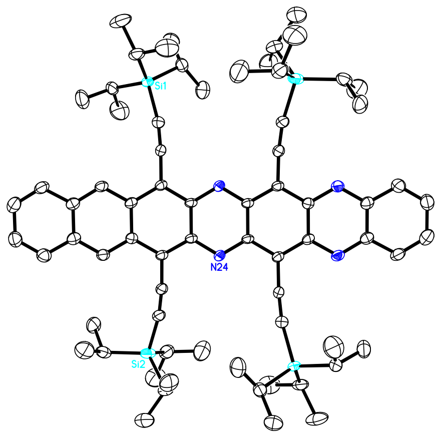

Table S16. Crystal data and structure refinement for **TAH**.

CCDC number	2081164	
Empirical formula	$C_{70}H_{94}N_4Si_4$	
Formula weight	1103.85	
Temperature	200(2) K	
Wavelength	0.71073 Å	
Crystal system	monoclinic	
Space group	$P2_1/c$	
Z	2	
Unit cell dimensions	a = 9.4940(10) Å	α = 90 deg.
	b = 20.153(2) Å	β = 91.266(3) deg.
	c = 17.1397(19) Å	γ = 90 deg.
Volume	3278.7(6) $Å^3$	
Density (calculated)	1.12 g/cm^3	
Absorption coefficient	0.13 mm^{-1}	
Crystal shape	column	
Crystal size	0.163 x 0.031 x 0.021 mm^3	
Crystal colour	green	
Theta range for data collection	1.6 to 25.1 deg.	
Index ranges	$-11 \leq h \leq 11$, $-24 \leq k \leq 24$, $-20 \leq l \leq 20$	
Reflections collected	30908	
Independent reflections	5837 (R(int) = 0.1134)	
Observed reflections	3399 ($I > 2\sigma(I)$)	
Absorption correction	Semi-empirical from equivalents	
Max. and min. transmission	0.96 and 0.75	
Refinement method	Full-matrix least-squares on F^2	
Data/restraints/parameters	5837 / 608 / 394	
Goodness-of-fit on F^2	1.00	
Final R indices (I>2sigma(I))	R1 = 0.060, wR2 = 0.115	
Largest diff. peak and hole	0.28 and -0.26 $eÅ^{-3}$	

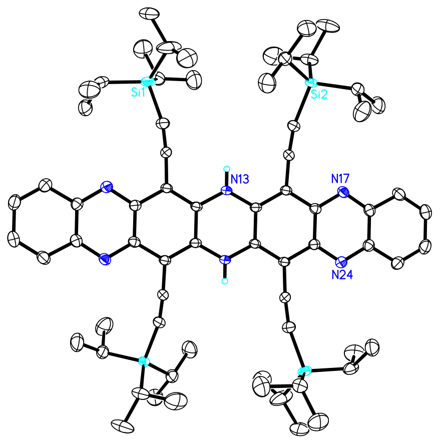

Table S17. Crystal data and structure refinement for **HAH**.

CCDC number	2081165	
Empirical formula	$C_{68}H_{93.12}N_6Si_4$	
Formula weight	1106.96	
Temperature	200(2) K	
Wavelength	0.71073 Å	
Crystal system	monoclinic	
Space group	$P2_1/c$	
Z	2	
Unit cell dimensions	a = 9.4977(3) Å	α = 90 deg.
	b = 20.0687(7) Å	β = 90.9157(8) deg.
	c = 17.0470(6) Å	γ = 90 deg.
Volume	3248.85(19) $Å^3$	
Density (calculated)	1.13 g/cm^3	
Absorption coefficient	0.14 mm^{-1}	
Crystal shape	column	
Crystal size	0.188 x 0.058 x 0.044 mm^3	
Crystal colour	red	
Theta range for data collection	1.6 to 29.9 deg.	
Index ranges	$-13 \leq h \leq 13$, $-26 \leq k \leq 26$, $-23 \leq l \leq 23$	
Reflections collected	39075	
Independent reflections	8611 (R(int) = 0.0484)	
Observed reflections	5943 ($I > 2\sigma(I)$)	
Absorption correction	Semi-empirical from equivalents	
Max. and min. transmission	0.96 and 0.92	
Refinement method	Full-matrix least-squares on F^2	
Data/restraints/parameters	8611 / 261 / 362	
Goodness-of-fit on F^2	1.03	
Final R indices (I>2sigma(I))	R1 = 0.053, wR2 = 0.122	
Largest diff. peak and hole	0.69 and -0.67 $eÅ^{-3}$	

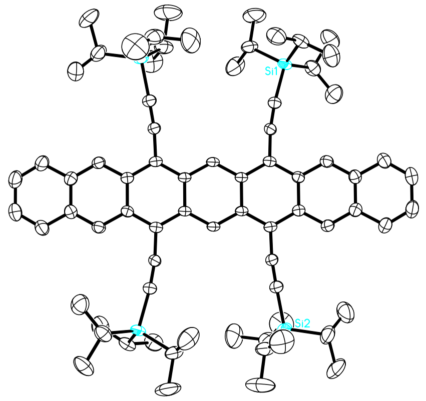

Table S18.Crystal data and structure refinement for **Hep** (polymorph I).

CCDC number	2254465	
Empirical formula	$C_{74}H_{98}Si_4$	
Formula weight	1099.88	
Temperature	200(2) K	
Wavelength	0.71073 Å	
Crystal system	monoclinic	
Space group	$P2_1/c$	
Z	2	
Unit cell dimensions	a = 15.9917(13) Å	α = 90 deg.
	b = 15.3854(12) Å	β = 113.1214(12) deg.
	c = 15.0415(12) Å	γ = 90 deg.
Volume	3403.5(5) Å^3	
Density (calculated)	1.07 g/cm^3	
Absorption coefficient	0.13 mm^{-1}	
Crystal shape	lanceolate	
Crystal size	0.222 x 0.082 x 0.060 mm^3	
Crystal colour	green	
Theta range for data collection	1.4 to 29.8 deg.	
Index ranges	$-22 \le h \le 22$, $-20 \le k \le 21$, $-20 \le l \le 20$	
Reflections collected	41467	
Independent reflections	9364 (R(int) = 0.0602)	
Observed reflections	5981 (I > 2σ(I))	
Absorption correction	Semi-empirical from equivalents	
Max. and min. transmission	0.96 and 0.91	
Refinement method	Full-matrix least-squares on F^2	
Data/restraints/parameters	9364 / 0 / 364	
Goodness-of-fit on F^2	1.02	
Final R indices (I>2sigma(I))	R1 = 0.055, wR2 = 0.128	
Largest diff. peak and hole	0.32 and -0.26 eÅ^{-3}	

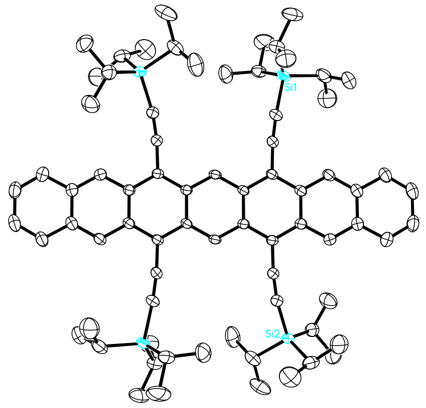

Table S19.Crystal data and structure refinement for **Hep** (polymorph **II**).

CCDC number	2254466	
Empirical formula	$C_{86}H_{110}Si_4$	
Formula weight	1256.09	
Temperature	200(2) K	
Wavelength	0.71073 Å	
Crystal system	triclinic	
Space group	$P\bar{1}$	
Z	1	
Unit cell dimensions	a = 9.7872(5) Å	α = 67.7713(13) deg.
	b = 13.6946(8) Å	β = 86.5928(13) deg.
	c = 15.7997(9) Å	γ = 78.0734(14) deg.
Volume	1917.55(19) Å^3	
Density (calculated)	1.09 g/cm^3	
Absorption coefficient	0.12 mm^{-1}	
Crystal shape	plate	
Crystal size	0.287 x 0.080 x 0.034 mm^3	
Crystal colour	green	
Theta range for data collection	1.4 to 28.6 deg.	
Index ranges	$-13 \leq h \leq 13$, $-18 \leq k \leq 18$, $-20 \leq l \leq 21$	
Reflections collected	37448	
Independent reflections	9462 (R(int) = 0.0614)	
Observed reflections	5813 ($I > 2\sigma(I)$)	
Absorption correction	Semi-empirical from equivalents	
Max. and min. transmission	0.96 and 0.89	
Refinement method	Full-matrix least-squares on F^2	
Data/restraints/parameters	9462 / 300 / 433	
Goodness-of-fit on F^2	1.03	
Final R indices (I>2sigma(I))	R1 = 0.057, wR2 = 0.143	
Largest diff. peak and hole	0.47 and -0.38 eÅ^{-3}	

15. Calculated Charge Carrier Mobilities

The theoretical charge carrier mobilities were calculated as described by Miao *et al.*[11] from the Einstein-Smoluchowski[12] equation at 25 °C:

$$\mu = \frac{eD}{k_B T}$$

where e is the elementary charge, D the diffusion coefficient, k_B the Boltzmann constant and T the temperature. The diffusion coefficient D[13] was approximated by

$$D = \frac{1}{2n} \sum_i r_i^2 W_i P_i$$

$$P_i = \frac{W_i}{\sum_i W_i}$$

where n is the spatial dimensionality and i represents a specific hopping pathway with the hopping distance r (intermolecular center to center distance of different dimers), the hopping rate W and the hopping probability P. The hopping rate W was evaluated from the Marcus-Hush equation [13]

$$W = \frac{V^2}{\hbar} \left(\frac{\pi}{\lambda k_B T} \right)^{1/2} \exp\left(-\frac{\lambda}{4 k_B T} \right)$$

where V is the transfer integral, which was calculated using ADF[14] (ADF2021.106, PW91/TZP), and λ is the reorganization energy, which was estimated through the four point method.[15] For this purpose, the molecules were optimized in the gas phase for the neutral and the anionic species at the B3LYP/def2-SVP level of theory. Using the optimized geometries energies were calculated with the def2-TZVP basis set.

$$\lambda = \lambda_1 + \lambda_2$$

$$\lambda_1 = E_{(M^-)} - E_{(M)}$$

$$\lambda_2 = E_{(\overline{M})} - E_{(\overline{M}^-)}$$

$E_{(M)}$ and $E_{(\overline{M}^-)}$ are the energies of the optimized neutral and anionic structures while $E_{(M^-)}$ represents the neutral energy of the optimized anionic structure and $E_{(\overline{M})}$ represents the anionic energy of the optimized neutral structure.

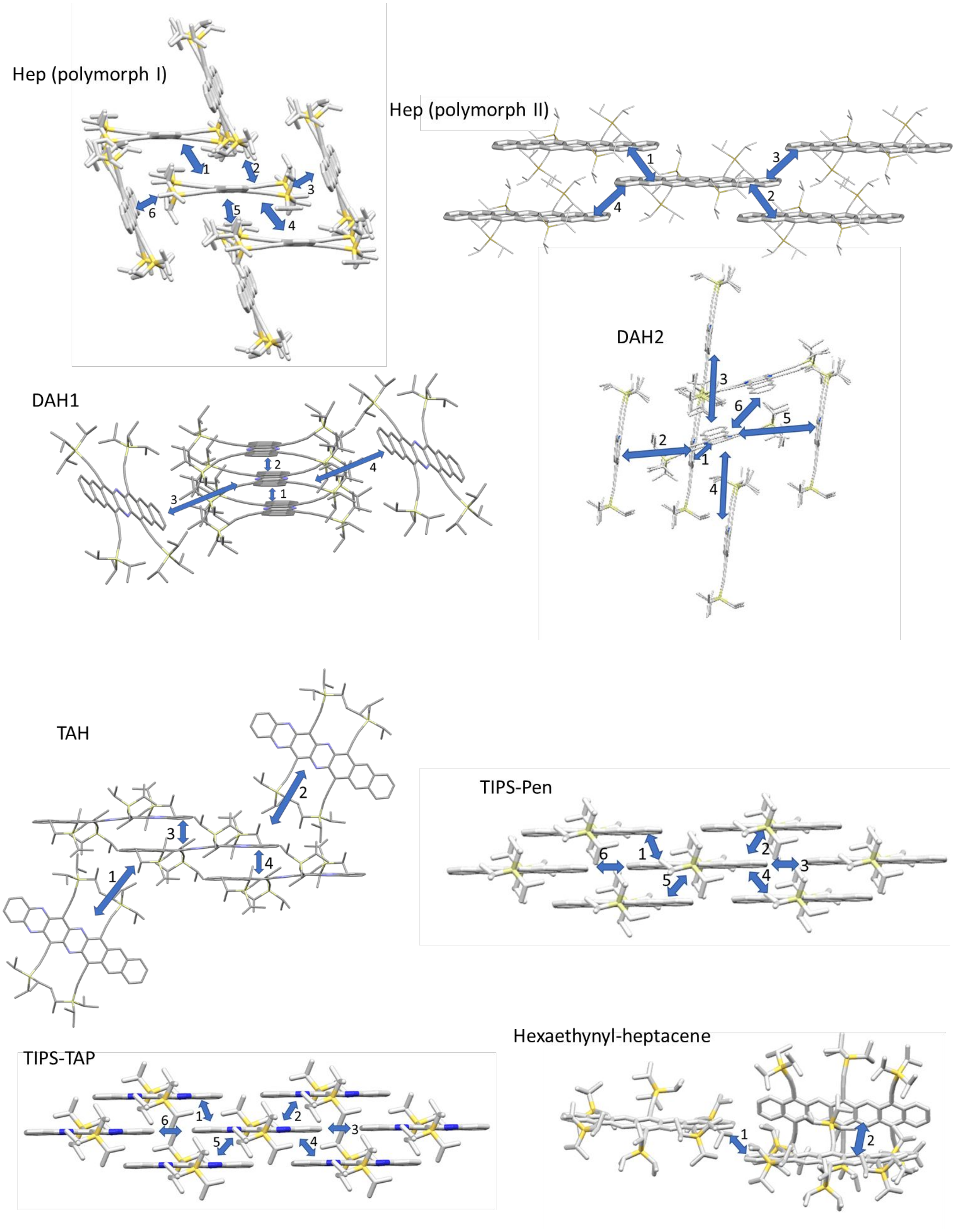

Figure S69. Dimer pairs of (aza)heptacenes, **TIPS-Pen** and **TIPS-TAP** used for the calculation of transfer integrals.

16. OFET Fabrication and Measurement

A sliced, highly p-doped silicon wafer (R < 0.005 Ω) with 100 nm thick thermally grown SiO_2 was successively cleaned via ultra-sonication in acetone, isopropanol and ethanol, each for 10 min. It was washed with water and dried under a stream of nitrogen. The wafer was placed in freshly produced Caro´s acid (H_2SO_4/H_2O_2 3:1) and was heated to 100°C for 20 min. After cleaning with water and drying, a 150 mM solution of $Al(NO_3)_3$x9 H_2O in ethanol was spin-coated (5000 rpm; 40 s) onto the substrate. Right after that the wafer was heated to 300 °C for 30 min. For the formation of the self-assembled monolayer, the substrate was placed in a 15.0 mM solution of 12-cyclohexyldodecylphosphonic acid (CDPA)[16] in isopropanol at room temperature for 16 h. Then the substrate was cleaned via ultra-sonication in isopropanol for 10 min, rinsed with water and dried in a stream of nitrogen.

The capacitance of the dielectric layer amounted to 26 nF cm^{-2}.

Drop-cast thin-films were prepared by dropping the prepared DCM solution (0.5 – 1.0 mg/mL) onto the substrate covering the wafer. Electrode formation was achieved by depositing a 40 nm thick layer of gold was deposited through a shadow mask onto the organic layer in a vacuum evaporator at a pressure below 2 x 10^{-6} bar. Transistor characteristics were measured with a semiconductor characterization system (Keithley 4200-SCS) in a nitrogen filled glove box. The field effect mobilities were determined in the saturated regime using the equation I_{DS}=$(W/2L)C_i\mu(V_G-V_{th})^2$, where I_{DS} is the source-drain current, W is the channel width, L the channel length, C_i is the capacitance per unit area of the gate dielectric layer, μ is the field effect mobility, and V_{th} is the threshold voltage.

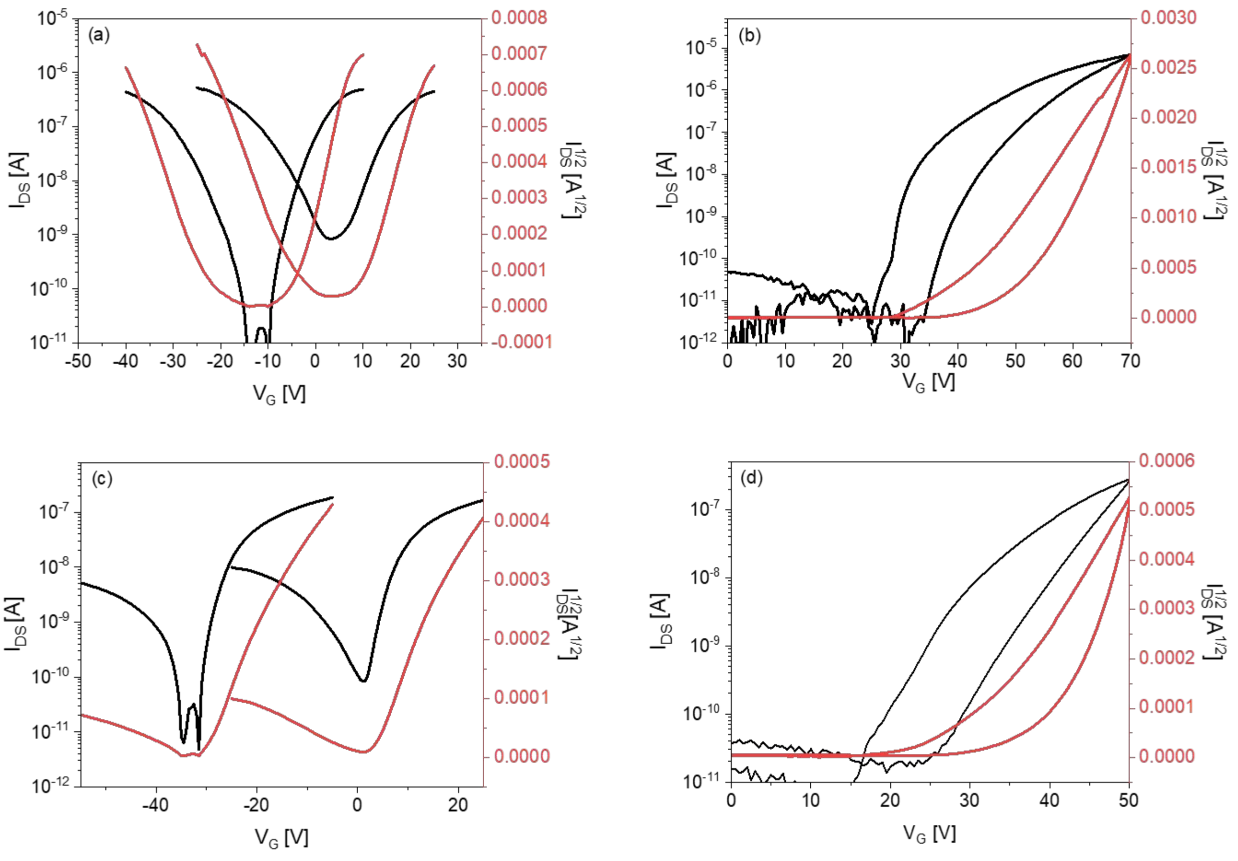

Figure S70. Transfer characteristics of (a) **Hep** (b) **DAH1**, (c) **DAH2** and (d) **TAH**.

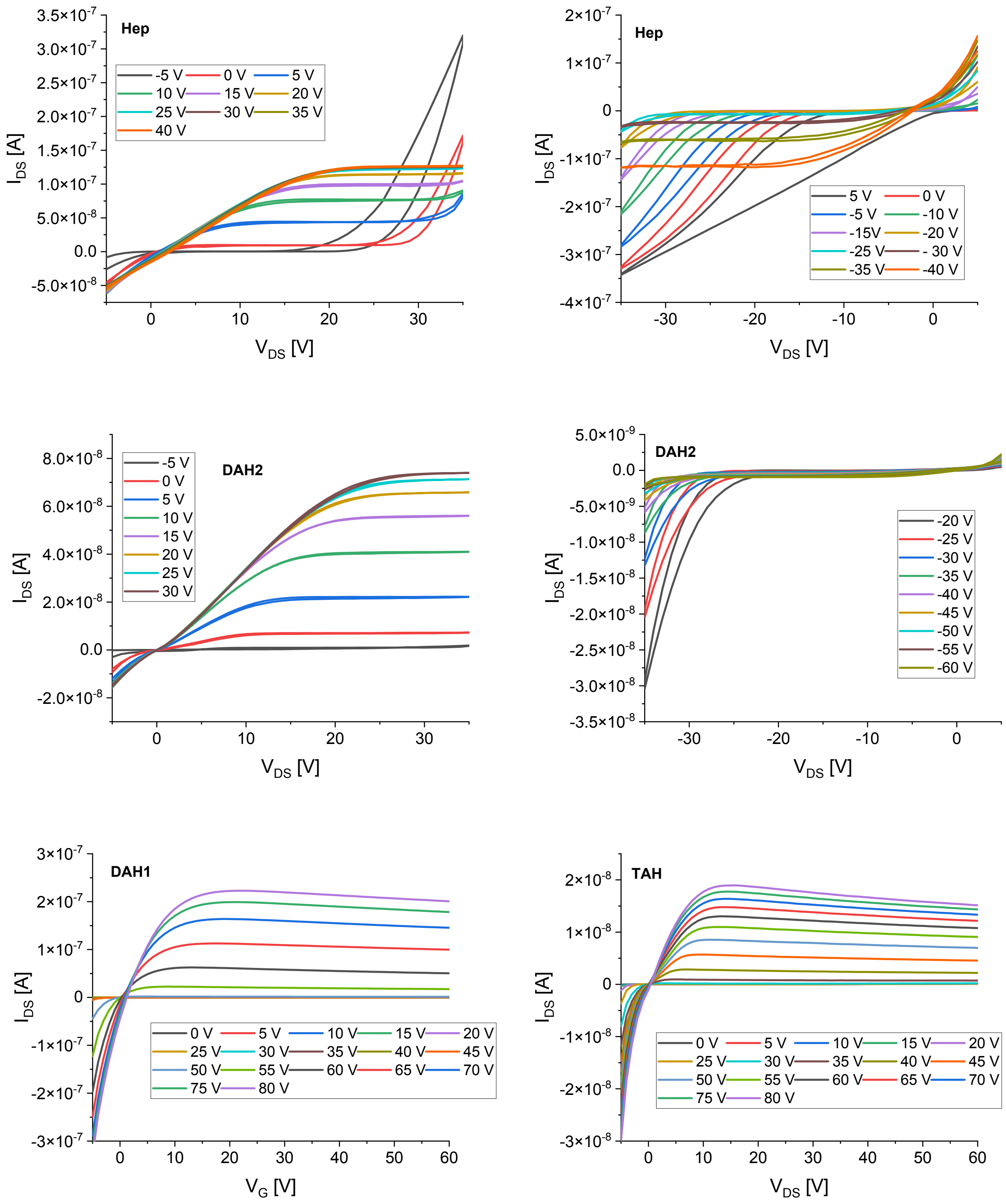

Figure S71. Output characteristics of **Hep, DAH1, DAH2** and **TAH**.

17.Film Morphology

XRD characterization of thin-films of the heptacenes on the CDPA modified substrates were carried out on a Rigaku Smartlab X-Ray Diffractometer with assignment to the (hkl) values. The data shows:

DAH1: X-ray diffraction peaks of the film does not match with the powder pattern simulated from the single crystal structure. (d_{film} = 16.58 Å).

DAH2: X-ray diffraction peaks of the film correspond to the (001) diffraction derived from the single crystal with interlayer spacing $d_{crystall}$ (001) = 15.64 Å. (d_{film} (00l) = 15.66 Å).

TAH: X-ray diffraction peaks of the film does not match with the powder pattern simulated from the single crystal structure. (d_{film} = 16.58 Å).

HEP: X-ray diffraction peaks of the film correspond to the (h00) diffraction derived from the single crystal with interlayer spacing $d_{crystall}$ (100) = 14.71 Å. (d_{film} (h00) = 14.58 Å).

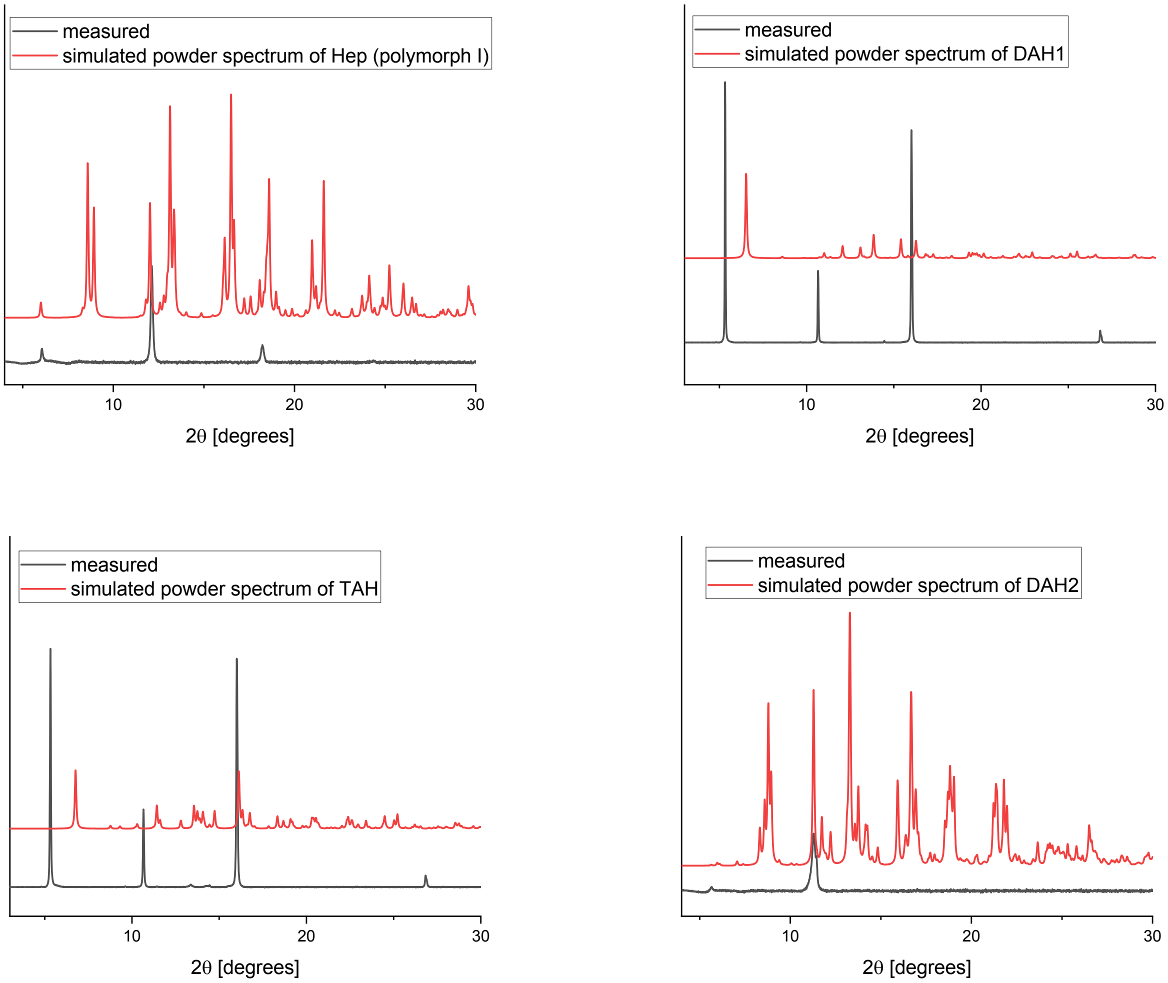

Figure S72. XRD characterization of thin films of (aza)heptacenes.

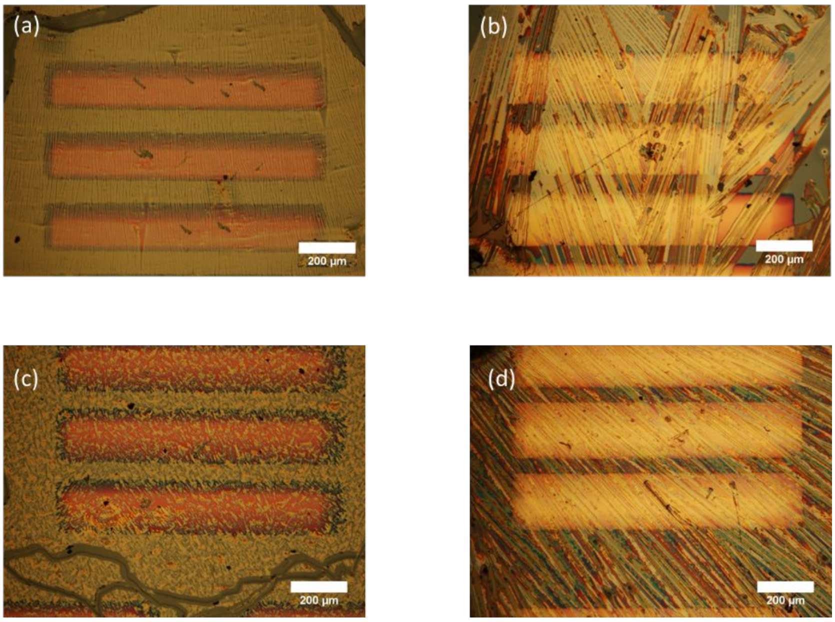

Figure S73. Polarized micrographs of exemplary measured channels of (a) **Hep**, (b) **DAH1**, (c) **DAH2** and (d) **TAH**.

18. References

[1] G. R. Fulmer, A. J. M. Miller, N. H. Sherden, H. E. Gottlieb, A. Nudelman, B. M. Stoltz, J. E. Bercaw, K. I. Goldberg, *Organometallics* **2010**, 29, 2176-2179.

[2] Gaussian 16, Revision C.01, M. J. Frisch, G. W. Trucks, H. B. Schlegel, G. E. Scuseria, M. A. Robb, J. R. Cheeseman, G. Scalmani, V. Barone, G. A. Petersson, H. Nakatsuji, X. Li, M. Caricato, A. V. Marenich, J. Bloino, B. G. Janesko, R. Gomperts, B. Mennucci, H. P. Hratchian, J. V. Ortiz, A. F. Izmaylov, J. L. Sonnenberg, D. Williams-Young, F. Ding, F. Lipparini, F. Egidi, J. Goings, B. Peng, A. Petrone, T. Henderson, D. Ranasinghe, V. G. Zakrzewski, J. Gao, N. Rega, G. Zheng, W. Liang, M. Hada, M. Ehara, K. Toyota, R. Fukuda, J. Hasegawa, M. Ishida, T. Nakajima, Y. Honda, O. Kitao, H. Nakai, T. Vreven, K. Throssell, J. A. Montgomery, Jr., J. E. Peralta, F. Ogliaro, M. J. Bearpark, J. J. Heyd, E. N. Brothers, K. N. Kudin, V. N. Staroverov, T. A. Keith, R. Kobayashi, J. Normand, K. Raghavachari, A. P. Rendell, J. C. Burant, S. S. Iyengar, J. Tomasi, M. Cossi, J. M. Millam, M. Klene, C. Adamo, R. Cammi, J. W. Ochterski, R. L. Martin, K. Morokuma, O. Farkas, J. B. Foresman, and D. J. Fox, Gaussian, Inc., Wallingford CT, **2016**.

[3] B. D. Lindner, J. U. Engelhart, O. Tverskoy, A. L. Appleton, F. Rominger, A. Peters, H. J. Himmel, U. H. F. Bunz, *Angew. Chem. Int. Ed.* **2011**, 50, 8588-8591.

[4] A. L. Appleton, S. Miao, S. M. Brombosz, N. J. Berger, S. Barlow, S. R. Marder, B. M. Lawrence, K. I. Hardcastle, U. H. F. Bunz, *Org. Lett*. **2009**, *11*, 5222-5225.

[5] J. U. Engelhart, O. Tverskoy, U. H. F. Bunz, *J. Am. Chem. Soc.* **2014,** *136*, 15166-15169.

[6] W. Zong, N. Hippchen, B. Dittmar, M. Elter, P. Ludwig, F. Rominger, J. Freudenberg, U. H. F. Bunz, *Asian J. Org. Chem.* **2023**, *12*, e202300462.

[7] N. Zeitter, N. Hippchen, S. Maier, F. Rominger, A. Dreuw, J. Freudenberg, U. H. F. Bunz, *Angew. Chem. Int. Ed.* **2022**, *61*, e202200918.

[8] S. K. Park, T. N. Jackson, J. E. Anthony, D. A. Mourey, *Appl. Phys. Lett.* **2007,** *91*, 063514.

[9] X. Xu, Y. Yao, B. Shan, X. Gu, D. Liu, J. Liu, J. Xu, N. Zhao, W. Hu, Q. Miao, *Adv. Mater.* **2016**, *28*, 5276-5283.

[10] N. Zeitter, N. Hippchen, P. Baur, T. Unterreiner, F. Rominger, J. Freudenberg, U. H. F. Bunz. *Org. Mater.* **2023**, DOI: 10.1055/a-2241-0243.

[11] M. Chu, J. X. Fan, S. J. Yang, D. Liu, C. F. Ng, H. L. Dong, A. M. Ren, Q. Miao, *Adv. Mater.* **2018**, 30, e1803467.

[12] V. Coropceanu, J. Cornil, D. A. da Silva Filho, Y. Olivier, R. Silbey, J. L. Bredas, *Chem. Rev.* **2007**, 107, 926-952.

[13] S. H. Wen, A. Li, J. Song, W. Q. Deng, K. L. Han, W. A. Goddard, *J. Phys. Chem. B* **2009**, 113, 8813-8819.

[14] ADF 2022.1, SCM, Theoretical Chemistry, Vrije Universiteit, Amsterdam, The Netherlands.

[15] J. L. Bredas, D. Beljonne, V. Coropceanu, J. Cornil, *Chem. Rev.* **2004**, 104, 4971-5004.

[16] D. Liu, Z. He, Y. Su, Y. Diao, S. C. B. Mannsfeld, Z. Bao, J. Xu, Q. Miao, *Adv. Mater.* **2014**, 26, 7190-7196.
